# Synthesis and Reduction of Super‐Bulky Bis(anilido)acridanide Aluminium Complexes

**DOI:** 10.1002/chem.71614

**Published:** 2026-08-21

**Authors:** Matthew J. Evans, Joseph M. Parr, Cameron Jones

**Affiliations:** ^1^ School of Chemistry Monash University Clayton Victoria Australia; ^2^ College of Science and Engineering Flinders University Adelaide South Australia Australia

**Keywords:** aluminium chemistry, bis(anilido)acridanide complexes, main‐group chemistry

## Abstract

Super‐bulky bis(anilido)acridanide pro‐ligands (NNN^Ar^)H_3_ (NNN^Ar^ = 4,5‐bis(anilido)‐2,7,9,9‐tetramethylacridanide, anilido = NAr, Ar = DCHP, 2,6‐dicyclohexylphenyl; TCHP, 2,4,6‐tricyclohexylphenyl) have been prepared in high yields. The reaction of (NNN^TCHP^)H_3_ with trimethylaluminium affords bimetallic complex [Al_2_Me_3_(NNN^TCHP^)] which reacts with iodine to form the corresponding monometallic complex [Al(NNN^TCHP^)(I)_2_]. The reduction of [Al(NNN^TCHP^)(I)_2_] with sodium and potassium containing reducing agents was explored. From potassium reductions, only complexes K[Al(NNN^TCHP^)(I)] (from K/KI) and [Al(NNN^TCHP^)(TMEDA)] (from KC_8_/TMEDA, TMEDA = *N,N,N’,N’*‐tetramethyl ethylenediamine) could be isolated. In contrast, aluminium(III) hydride [Na(C_6_H_6_)][Al(NNN^TCHP^)(H)] was formed from reduction of [Al(NNN^TCHP^)(I)_2_] in benzene with excess sodium metal (mirror). This complex is unstable and readily decomposes in the presence of ethers, such as 2.2.2‐cryptand, to give the aluminium(III) complex [Al(NNN^TCHP^)(2.2.2‐cryptand)].

## Graphical abstract

A new generation of super‐bulky bis(anilido)acridanide ligands has been developed and used in the preparation of aluminium(III) complexes.

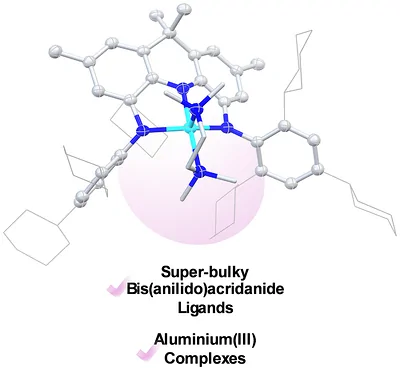

## 1 Introduction

Aluminium, the most abundant metal in Earth's crust, has received considerable attention in recent years for its versatile roles across synthetic chemistry [1, 2, 3]. Complementary to its abundance, the cost of aluminium reagents position them as attractive alternatives to transition metal‐based systems traditionally used in chemical synthesis [4, 5]. Despite intensive investigations into the chemistry of aluminium, it was only recently that the first redox active aluminium catalyst **I** was reported (Figure 1) [6, 7]. This compound utilized a 1,8‐diarylcarbazol‐9‐yl ligand set to stabilize aluminium across the reversible +1 and +3 oxidation states, highlighting the importance of ligand design to enable functional chemistry for the main‐group elements.

**FIGURE 1:**
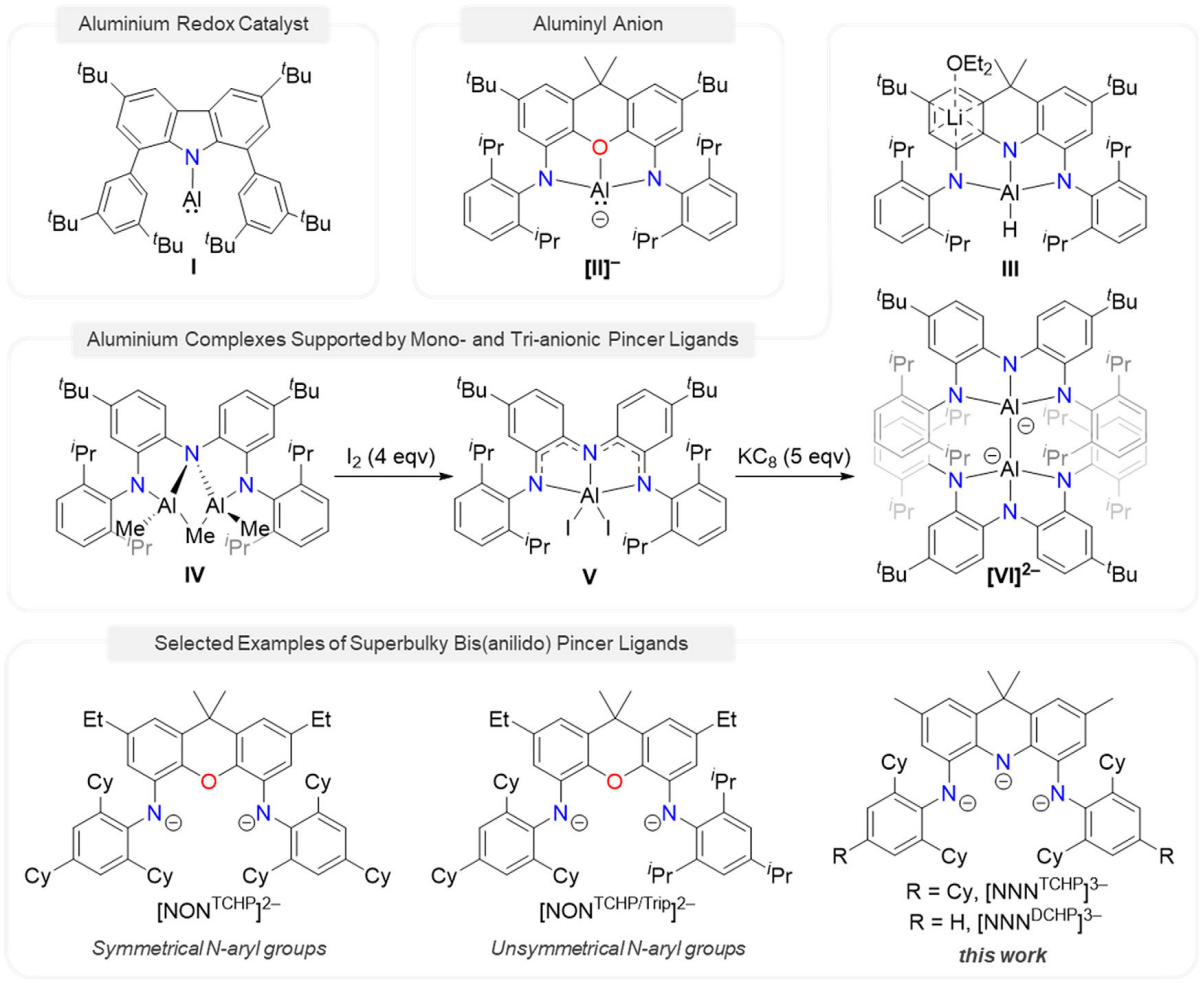
Selected examples of previously reported aluminium complexes stabilized by bulky ligands, and super‐bulky bis(anilido) pincer ligands relevant to the current study.

Bis(anilido)xanthene pincer ligands, first introduced by Emslie and coworkers in 2007 [8], have become prevalent across synthetic organometallic chemistry due to their ability to stabilize metal centers in variable oxidation states [9, 10, 11, 12]. In 2018, Aldridge and Goicoechea reported the first aluminyl anion [**II**]^–^ (an aluminium(I) complex) featuring an example of Emslie's ligands, *viz*. the bulky NON^Dipp^ ligand system (NON^Dipp^ = 4,5‐bis(2,6‐diisopropylanilido)‐2,7‐di‐*tert*‐butyl‐9,9‐dimethylxanthene) [13]. Since the report on [**II**]^–^, a range of metal‐aluminyl complexes have since been prepared, and their use as reducing agents, and as precursors to heterobimetallic aluminium complexes, is now well‐established [14, 15, 16]. In contrast, an extension of these ligands to their trianionic bis(anilido)acridanide counterparts has only recently gained traction [17, 18, 19]. Earlier this year, Wang and co‐workers reported the preparation of bis(anilido)acridanide aluminium(III) complexes (e.g. **III**) stabilized by the NNN^Dipp^ ligand system (NNN^Dipp^ = 4,5‐bis(2,6‐diisopropylanilido)‐2,7‐di‐*tert*‐butyl‐9,9‐dimethylacridanide) [20]. A similar, flexible trianionic pincer ligand, ^F^NNN^Dipp^ (^F^NNN^Dipp^ = bis(2‐(2,6‐diisopropylanilido)‐4‐(*tert*‐butyl)phenyl)amide), was reported by the same research group and used to stabilize aluminium in the +3 and +2 oxidation states [21]. The reaction of (^F^NNN^Dipp^)H_3_ with trimethylaluminium yielded dinuclear complex **IV**, which was converted into monomeric aluminium(III) complex **V** by treatment with iodine (4 equivalents). Importantly, reduction of **V** afforded the corresponding dianionic dialane(II) complex [**VI**]^2–^, presumably through homocoupling of transient aluminium(II) radicals.

Within our research group, we have been working on the development of ligand systems comprising sterically demanding N‐aryl groups. In this arena, we have developed super‐bulky xanthene derivatives, both symmetrically (*e.g*. NON^TCHP^: 4,5‐bis(2,4,6‐tricyclohexylanilido)‐2,7‐diethyl‐9,9‐dimethyl‐xanthene) [22] and unsymmetrically (*e.g*. NON^TCHP/Trip^: (4‐(2,4,6‐tricyclohexylanilido)‐5‐(2,4,6‐triisopropylanilido)‐2,7‐diethyl‐9,9‐dimethylxanthene) [23] substituted at their N‐centers, in order to promote unprecedented reactivity at coordinated main‐group element centers. So far, we have used complexes derived from these ligands to facilitate, for example, dinitrogen reduction at magnesium and calcium through reduction of [**M**(NON^TCHP^)] (**M** = Mg or Ca) in aliphatic solvents [24, 25]. As part of our ongoing work in this space, we wanted to translate this design strategy to related super‐bulky bis(anilido)acridanide ligands and examine their use in molecular aluminium chemistry.

## 2 Results and Discussion

Bis(anilido)acridanide pro‐ligands, (NNN^TCHP^)H_3_ and (NNN^DCHP^)H_3_, were prepared in modest or good yields by the palladium‐catalyzed cross‐coupling reaction of the appropriate aniline with 1,8‐dibromo‐3,6,9,9‐tetramethyl‐9,10‐dihydroacridine (Scheme 1). This procedure mirrors that used to prepare the similar, less bulky pro‐ligand (NNN^Dipp^)H_3_ reported by Wang and coworkers [17]. Single crystals of (NNN^TCHP^)H_3_ and (NNN^DCHP^)H_3_ could be obtained from saturated hexane solutions stored at –30 °C. Their molecular structures were determined unambiguously by single crystal X‐ray diffraction measurements (see Supporting Information for further details). Both pro‐ligands require storage under an inert atmosphere to prevent decomposition in air, as noted for other pro‐ligands of this type [26].

**SCHEME 1:**
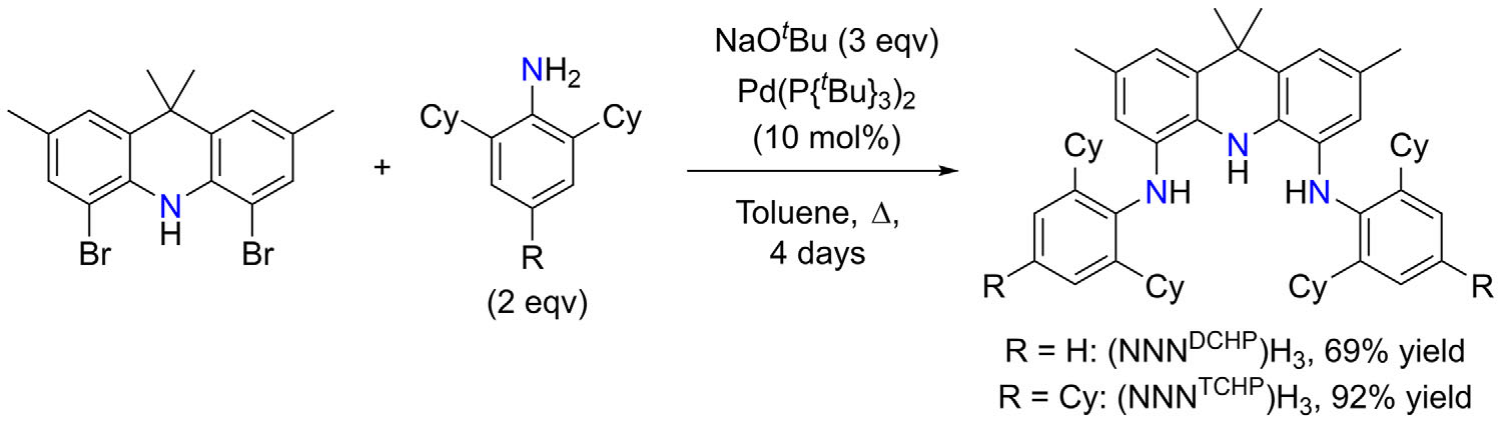
Preparation of super‐bulky bis(anilido)acridanide pro‐ligands (NNN^DCHP^)H_3_ and (NNN^TCHP^)H_3_.

We selected the highest yielding super‐bulky pro‐ligand, (NNN^TCHP^)H_3_, as the model system for this study. The reaction of (NNN^TCHP^)H_3_ and trimethylaluminium takes place at elevated temperatures to form bimetallic complex **1** in moderate yield (46%) after work‐up (Scheme 2). Attempts to form a monometallic complex of type “Al(NNN^TCHP^)” by varying the amount of trimethylaluminium were unsuccessful, returning complex **1** and/or (NNN^TCHP^)H_3_ as the only isolated products. The ^1^H NMR spectrum of **1** suggests an unsymmetrical coordination environment for the aluminium‐bound methyl groups with singlets located at *δ*_H_ ‐0.28 ppm (6H) and *δ*_H_ 0.54 ppm (3H), consistent with terminal and bridging binding modes, respectively. Treatment of **1** with iodine drives the formation of monometallic complex [Al(NNN^TCHP^)(I)_2_] (**2**) accompanied by oxidation of the acridanide framework across the NNN unit in the NNN^TCHP^ ligand, though the exact mechanism of this process is not known. With that said, the process requires 4 equivalents of iodine and results in the formal loss of aluminium triiodide and 3 equivalents of methyl iodide as per the transformation of **IV** to **V**, which feature related, but flexible, redox non‐innocent ligands [21].

**SCHEME 2:**
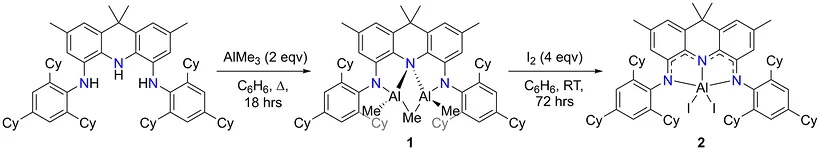
Preparation of super‐bulky bis(anilido)acridanide aluminium(III) complexes **1** and **2**.

This result can be alternatively achieved in a two‐step, one‐pot reaction from (NNN^TCHP^)H_3_ to give **2** in up to 79% yield after work‐up. The ^1^H NMR spectrum of **2** is indicative of a symmetrical coordination environment for the NNN^TCHP^ backbone protons, but is otherwise uninformative.

Crystals of **1** were obtained from a saturated hexane/benzene (10:1) mixture stored at room temperature (Figure 2a). In the solid‐state, **1** comprises a dinuclear Al_2_Me_3_ core with each aluminium center bound by a terminal (Al–C: 1.9262(17)–1.9278(18) Å) and bridging (Al–C: 2.1075(17)–2.1145(17) Å) methyl group. The coordination sphere at each aluminium center is completed by a κ^2^‐*N* binding mode to the NNN^TCHP^ ligand resulting in a distorted tetrahedral geometry. For **2**, the solid‐state structure reveals the presence of two aluminium‐bound iodide ligands (Figure 2b, Al–I: 2.5384(10) Å and 2.5936(10) Å). This is consistent with oxidation of the NNN^TCHP^ framework to give a tridentate, monoanionic κ^3^‐*N*‐chelating ligand resulting in a distorted trigonal bipyramidal geometry at the aluminium center. Oxidation of related bis(amido)acridanide‐type pincer ligands has previously been documented, highlighting their propensity to engage in redox processes [17, 18, 19, 20, 26, 27, 28].

**FIGURE 2:**
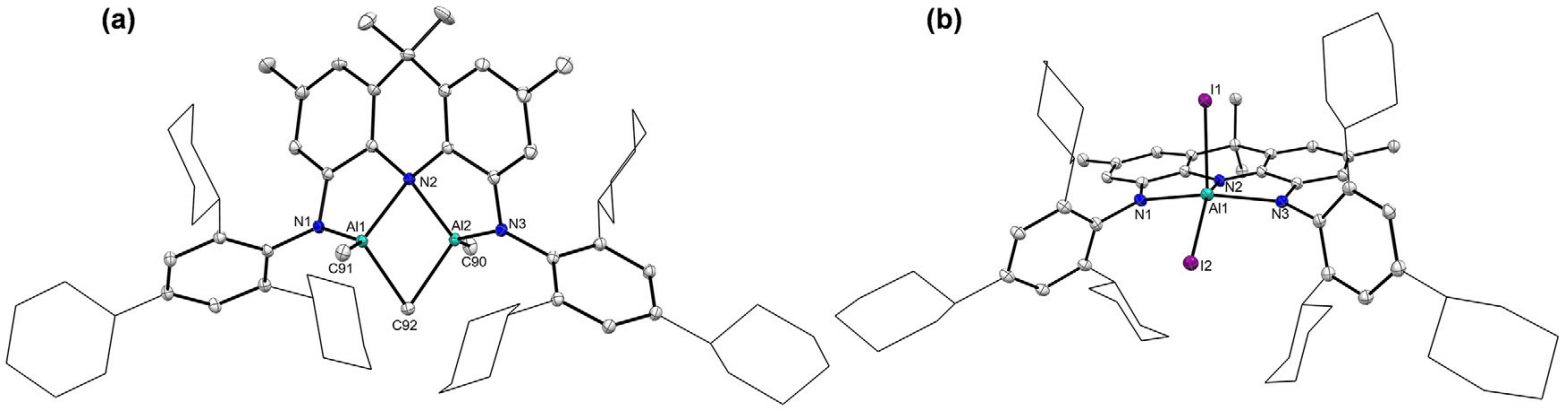
Molecular structure of (a) **1** and (b) **2**. Ellipsoids shown at 30% probability, hydrogen atoms omitted and cyclohexyl groups shown as wireframe for clarity.

Our initial attempts to reduce **2** using potassium‐based reducing agents (5 wt% K/KI [29], KC_8_, K metal) in methylcyclohexane resulted in the formation of red oily residues from which no crystalline products could be isolated. We then turned to the reaction of **2** with an excess of 5 wt% K/KI in benzene (Scheme 3). Analysis of the crude reaction mixture by ^1^H NMR spectroscopy indicated the formation of a complex mixture of products. In one instance, a low yield of colorless crystals of **3** were obtained after work‐up (Scheme 3).

**SCHEME 3:**
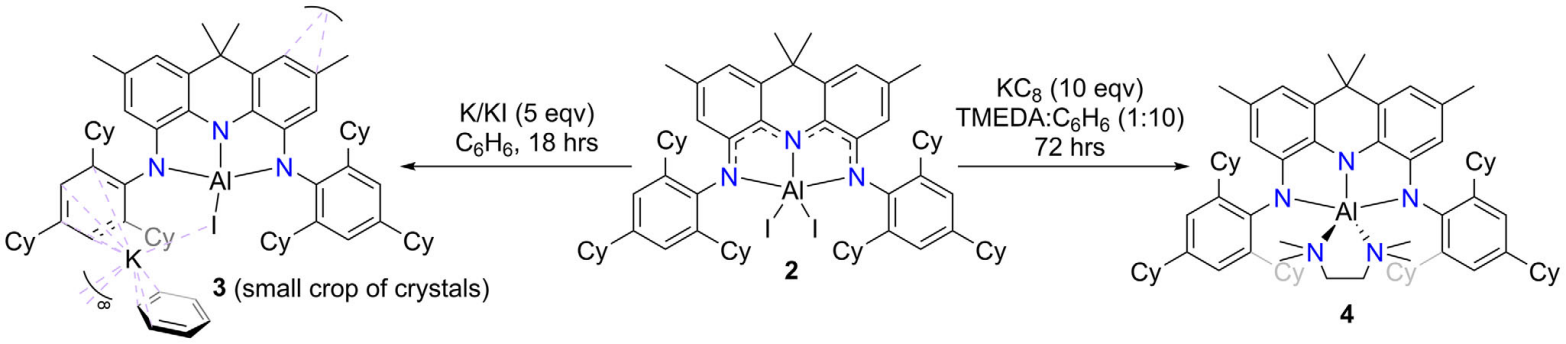
Reduction of **2** using potassium reducing agents K/KI (5 wt%) and KC_8_.

Structurally, the NNN^TCHP^ ligand backbone in **3** has been reduced to the trianion while the aluminium center remains in the +3 oxidation state (Figure 3a). This complex can be viewed as a potassium salt of [Al(NNN^TCHP^)(I)]^–^, where the iodide ligand (Al–I: 2.5619(11) Å) is bound to the aluminium center resulting in a distorted square planar geometry (*τ*_4_ = ∼0.37) [30]. The potassium cation in **3** serves as a charge‐balance to the [Al(NNN^TCHP^)(I)]^–^ component, forming a long‐range 1D polymer through K∙∙∙π(arene) interactions between these molecular units and benzene molecules in the solid‐state. Examples of planarization at a tetracoordinate aluminium center are rare [20, 31, 32, 33, 34, 35] and it is likely that the rigid NNN^TCHP^ backbone, paired with K∙∙∙I and K∙∙∙π(arene) interactions, enforce the observed distorted square planar geometry at the metal center. Due to the low yield of **3**, we were not able to obtain spectroscopic data on the compound.

**FIGURE 3:**
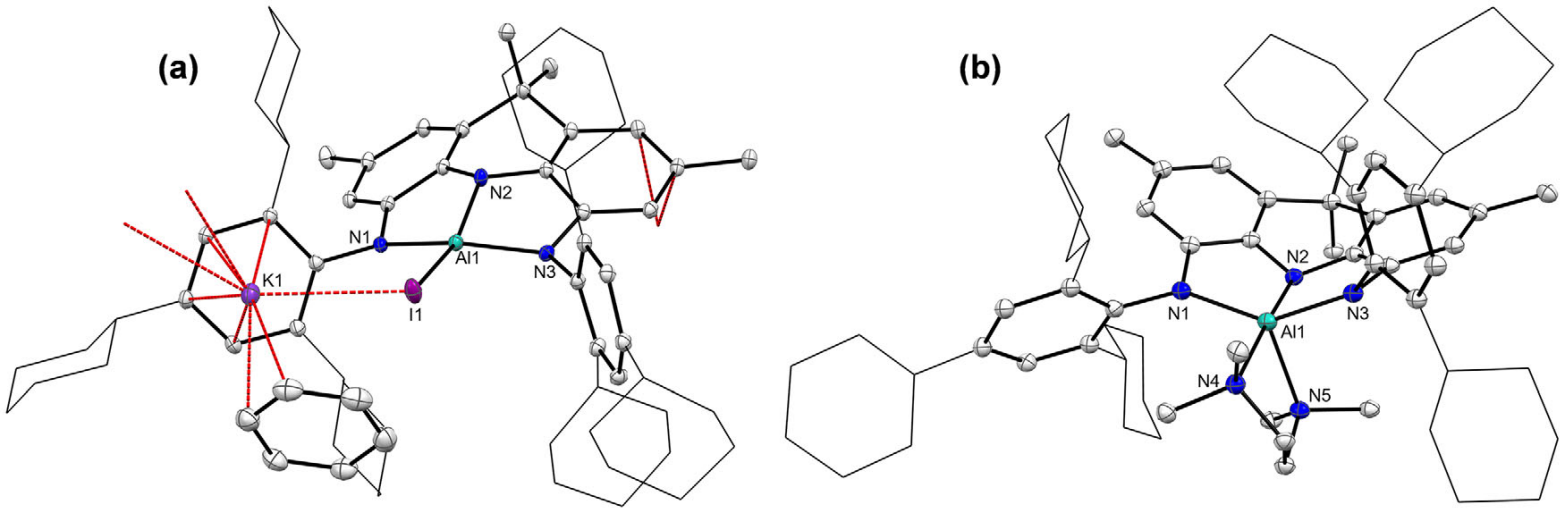
Molecular structure of (a) **3** and (b) **4**. Ellipsoids shown at 30% probability, hydrogen atoms omitted and cyclohexyl groups shown as wireframe for clarity.

In contrast to the formation of **3**, the reduction of **2** with KC_8_ in a benzene/TMEDA (1:10) mixture afforded the TMEDA adduct, [Al(NNN^TCHP^)(TMEDA)] (**4**), as colorless crystals in moderate yields (up to 64%) after work‐up. The ^1^H NMR spectrum of **4** shows two key signals for the TCHP aryl groups at *δ*_H_ 7.20 ppm (s, 2H) and 7.25 ppm (s, 2H), which suggests a relatively unsymmetrical coordination environment with respect to the NNN^TCHP^ ligand in solution. Additional resonances for TMEDA were located at *δ*_H_ 1.56 ppm (br s, 12H) and 2.37 ppm (br s, 4H), consistent with its complexation. The solid‐state structure of **4** confirms that the aluminium center remains in the +3 oxidation state and that reduction of the NNN^TCHP^ ligand to the trianion has taken place. The aluminium center in **4** adopts a geometry intermediate between square pyramidal and trigonal bipyramidal (*τ*_5_ = ∼0.53) [36] with Al–N bonds involving the NNN^TCHP^ and TMEDA ligands ranging in length from 1.8452(18) Å to 2.126(2) Å.

Given that our attempts to form low oxidation state aluminum complexes via potassium reduction of **2** were not successful, we alternatively explored the reduction of **2** with sodium metal (mirror, excess) in benzene (Scheme 4). Again, this generated a complex mixture of products and did not lead to a low oxidation state aluminum compound. However, we were able to isolate aluminium(III) hydride complex **5** in low but reproducible yields (up to 31%).

**SCHEME 4:**
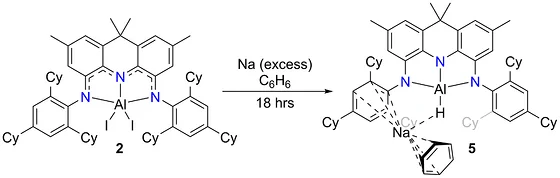
Reduction of **2** using sodium to give compound **5**.

Complex **5** decomposes rapidly upon exposure to air and moisture and is poorly soluble in aliphatic and arene solvents once crystallized. Satisfactory NMR spectroscopic data could only be obtained in the presence of ethereal solvents which result in the decomposition of **5** (*vide infra*). The single crystal x‐ray structure of **5** revealed significant disorder of the [Na(C_6_H_6_)]^+^ component over a symmetry element (Figure 4). However, the x‐ray diffraction data confirm the formation of bis(anilido)acridanide aluminium(III) hydride complex [Na(η^4^‐C_6_H_6_)][Al(NNN^TCHP^)(H)] (**5**). This is closely related to the structure of [Li(OEt_2_)][Al(NNN^Dipp^)(H)] (**III**), where the cation is instead situated above the acridanide backbone [20]. More evidence for the structure of **5** is provided by the Na∙∙∙π(arene) contacts (∼2.82–3.11 Å) which are in a range expected for such interactions [37, 38, 39, 40]. Compound **5** was further interrogated by computational methods based on DFT, employing the same level of theory as used by Su and Wang and coworkers for analysis of compound **III** (see Supporting Information for details) [20]. The geometry optimized structure of **5** shows good agreement with the solid‐state structure. This is borne out by the Al–N1 (XRD: 1.899(2) Å; DFT: 1.913 Å) and Al–N2 (XRD: 1.823(4) Å; DFT: 1.823 Å) bond lengths, and further, in the N1–Al–N2 (XRD: 85.10(9)°; DFT: 84.98°) and N2–Al–H (XRD: 144.32(10)°; DFT: 144.17°) bond angles. The Al–H stretching frequency was computed at *ν* = 1841.0 cm^−1^, when using the appropriate scaling factor. However, the highly air‐ and moisture sensitive nature of **5** precluded successful experimental FT‐IR analysis of compound **5**. A bonding analysis on compound **5**, including NBO and AIM calculations, can be found in the Supporting Information.

**FIGURE 4:**
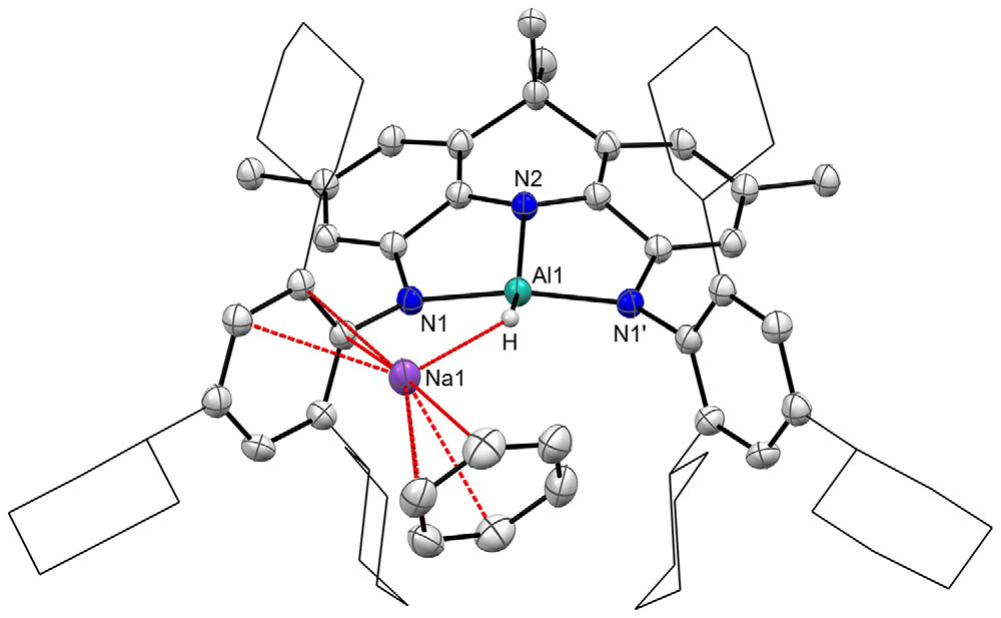
Molecular structure of **5**. Ellipsoids shown at 30% probability, hydrogen atoms omitted, and cyclohexyl groups shown as wireframe for clarity.

It is, perhaps, not surprising that the sodium and potassium reductions of **2** did not give a mononuclear aluminium(II) product, as examples of such complexes are exceedingly rare [41]. It is likely that the steric profile of the NNN^TCHP^ ligand impedes the dimerization of any generated aluminium(II) intermediates from the reduction of **2**, contrasting the reduction of Wang's related aluminium complex **V** with KC_8_ which affords the homocoupled aluminium(II) product [K(C_6_H_6_)]_2_[**VI**] [21]. Moreover, reactions of transient aluminium(II) complexes with benzene have been reported to form Birch‐type reduction products ([C_6_H_6_]^2–^) [42]. Similarly, recent studies have shown that (transient) aluminium(II) complexes can also facilitate the activation of various small molecules through diverse reaction pathways [43, 44, 45, 46]. In addition, the C─H and C─C bond activation of benzene by aluminyl complexes is now well documented [13, 39, 47, 48, 49, 50, 51, 52, 53, 54, 55]. Accordingly, it seems plausible that **5** may form via C─H activation of benzene by a transient aluminium(II) radical intermediate. If this is the case, we could not determine the fate of the phenyl fragment arising from that C─H activation.

Attempts to better define the coordination environment of the sodium cation in **5**, through the addition of 2.2.2‐cryptand, instead resulted in the formation of the neutral adduct **6** (Scheme 5). The molecular structure of **6** reveals a geometry intermediate between square pyramidal and trigonal bipyramidal (*τ*_5_ = ∼0.54) [36] at the aluminium center, which is κ^2^‐*O,O*‐chelated by the 2.2.2‐cryptand ligand (see Figure S37). The formation of **6** presumably arises from abstraction of NaH from **5**.

**SCHEME 5:**
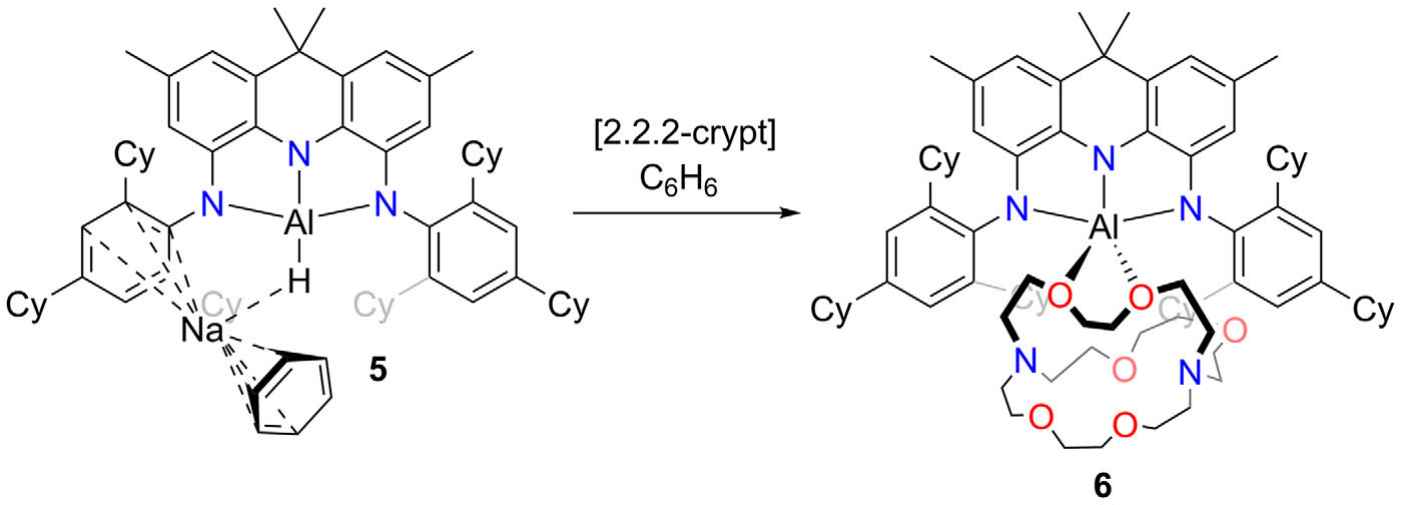
Reaction of **5** with 2.2.2‐cryptand to form adduct complex **6**.

## 3 Conclusion

In summary, we have reported the synthesis of super‐bulky bis(anilido)acridanide pro‐ligands (NNN^DCHP^)H_3_ and (NNN^TCHP^)H_3_. The dinuclear aluminium(III) methyl complex **1** was formed by the deprotonation of (NNN^TCHP^)H_3_ with two equivalents of trimethylaluminium. Transformation of **1** into the mononuclear aluminium(III) diiodide complex **2**, by reaction of four equivalents iodine, results in the oxidation of the NNN^TCHP^ ligand from being trianionic to monoanionic. Attempts to isolate products from the reduction of **2** in methylcyclohexane using potassium‐based reducing agents were unsuccessful. Instead, the reduction of **2** in benzene using K/KI or KC_8_/TMEDA afforded complexes **3** and **4**, respectively. Attention then turned to the reduction of **2** using sodium metal in benzene, which yielded the bimetallic sodium/aluminium hydride **5**, which is proposed to proceed *via* a transient aluminium(II) intermediate. The reaction of **5** with 2.2.2‐cryptand results in decomposition to adduct complex **6**. We are currently investigating the use of these super‐bulky bis(anilido)acridanide ligands in the preparation of other main‐group compounds.

## Conflicts of Interest

The authors declare no conflicts of interest.

## Supporting information



The authors have cited additional references within the Supporting Information [20, 56, 57, 58, 59, 60, 61, 62, 63, 64, 65, 66, 67, 68, 69, 70, 71, 72].
**Supporting File 1**: chem71614‐sup‐0001‐SuppMat.pdf

## Data Availability

The data that support the findings of this study are available in the supplementary material of this article. Deposition Numbers 2549934–2549941 contain the supplementary crystallographic data for this paper and are accessible free of charge by the joint Cambridge Crystallographic Data Centre and Fachinformationszentrum Karlsruhe Access Structures service www.ccdc.cam.ac.uk/structures.
