# Supplementary material for: Synthesis and Reduction of Super‐Bulky Bis(anilido)acridanide Aluminium Complexes
Source: Chemistry. 2026 Aug 21;32(42):e71614. doi: 10.1002/chem.71614 (PMC13755174; doi:10.1002/chem.71614)
Supplement: Supplementary file 1 [file CHEM-32-e71614-s001.pdf]

Electronic Supplementary Information (48 pages)

Contents

1. Syntheses and Spectra S2
2. Crystallographic Details S25
3. Computational Details S37
4. References S47

1. Syntheses and Spectra

General Considerations

All manipulations were carried out using standard Schlenk and glove box techniques under an atmosphere of high purity dinitrogen. Pentane and diethyl ether were distilled over Na/K alloy (50:50), while hexane, methylcyclohexane, benzene, toluene and THF were distilled over molten potassium. TMEDA was distilled over calcium hydride. ^{1}H and ^{13}C{^{1}H} NMR spectra were recorded on either Bruker Avance III 600 or Bruker Avance III 400 spectrometers at room temperature (unless otherwise stated) and were referenced to the resonances of the solvent used. FTIR spectra were collected for solid samples or Nujol mulls on an Agilent Cary 630 attenuated total reflectance (ATR) spectrometer. Microanalyses were carried out at by the Elemental Analysis Service at London Metropolitan University. Melting points were determined in sealed glass capillaries under dinitrogen and are uncorrected. 1,8-Dibromo-3,6,9,9-tetramethyl-9,10-dihydroacridine,[S1] 2,6-dicyclohexylaniline[S2] and 2,4,6-tricyclohexylaniline[S3] were prepared according to their reported literature procedures. Unless otherwise stated, chemicals were purchased from Sigma-Aldrich and used as received.

Preparation of 1,8-bis(2,4,6-tricyclohexylanilino)-3,6,9,9-tetramethyl-9,10-dihydroacridine, (NNN^{TCHP})H$_3$

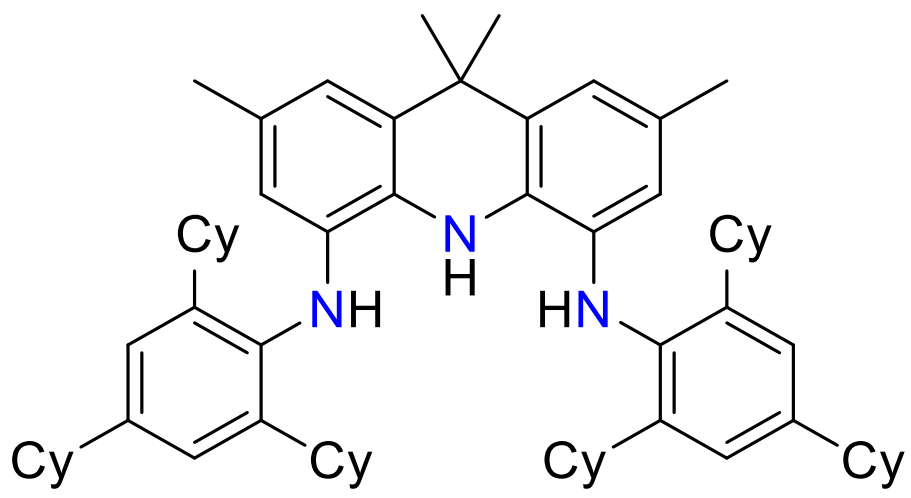

1,8-Dibromo-3,6,9,9-tetramethyl-9,10-dihydroacridine (2.00 g, 5.1 mmol), 2,4,6-tricyclohexylaniline (3.44 g, 10.1 mmol), sodium *tert*-butoxide (1.46 g, 15.2 mmol) and bis(tri-*tert*-butylphosphine) palladium(0) (260 mg, 0.51 mmol, 10 mol%) were charged to a flame dried J Young fitted Schlenk flask. Toluene (75 mL) was added to the combined solid masses and the flask heated to 120 °C for 4 days (under an atmosphere of N_2). The mixture was quenched with degassed water, extracted with toluene (2 x 50 mL) and dried over anhydrous magnesium sulphate. The volatiles were removed *in vacuo* and to the residue was added dry acetonitrile (50 mL). The mixture was sonicated for 1 hour and the product obtained by filtration as a pale green solid that was determined to be of high purity by multinuclear NMR spectroscopic measurements. **Yield**: 4.26 g, 92 %.

m.p.: 128 – 130 °C (dec).

^{1}H NMR (600 MHz, C_6D_6): δ 1.25 – 1.68 (m, 36H, Cy–$\underline{H}$), 1.71 (s, 6H, C(C$\underline{H}_3$)$_2$), 1.74 – 1.84 (m, 12H, Cy–$\underline{H}$), 1.89 – 1.97 (m, 8H, Cy–$\underline{H}$), 1.99 (s, 6H, C$\underline{H}_3$), 2.00 – 2.04 (m, 4H, Cy–$\underline{H}$), 2.46 – 2.65 (m, 2H, Cy–$\underline{H}$), 2.99 (m, 4H, Cy–$\underline{H}$), 4.84 (s, 2H, $_{\text{TCHP}}$N$\underline{H}$), 6.61 (d, J = 1.8 Hz, 2H, $_{\text{ACR}}$Ar–$\underline{H}$), 6.95 (d, J = 1.8 Hz, 2H, $_{\text{ACR}}$Ar–$\underline{H}$), 7.24 (s, 4H, $_{\text{TCHP}}$Ar–$\underline{H}$), 7.63 (s, 1H, $_{\text{ACR}}$N$\underline{H}$).

^{13}C{^{1}H} NMR (151 MHz, C_6D_6): δ 21.2 ($\underline{C}$H$_3$), 26.6, 26.7, 27.4, 27.4, 27.5, 27.8 (Cy–$\underline{C}$), 32.5 (C($\underline{C}$H$_3$)$_2$), 34.1, 35.1, 35.3 (Cy–$\underline{C}$), 35.6 ($\underline{C}$(CH$_3$)$_2$), 37.0, 39.7, 45.3 (Cy–$\underline{C}$), 118.6, 120.9, 123.4, 129.0, 129.8, 129.9, 134.1, 137.3, 142.6, 144.8 ($_{\text{ACR}}$Ar–$\underline{C}$, $_{\text{TCHP}}$Ar–$\underline{C}$).

I.R. (solid, cm^{-1}): 3383 (w), 2920 (s), 2850 (s), 1605 (m), 1493 (m), 1459 (m), 1446 (w), 1396 (w), 1357 (w), 1325 (w), 1288 (m), 1258 (w), 1239 (w), 1210 (w), 1176 (w), 1131 (w), 1027 (m), 997 (m), 950 (w), 889 (w), 861 (m), 841 (m), 807 (w), 759 (w), 727 (w), 710 (w), 647 (w).

Note: Darkens under aerobic conditions and is thus stored under an inert atmosphere in a glovebox.

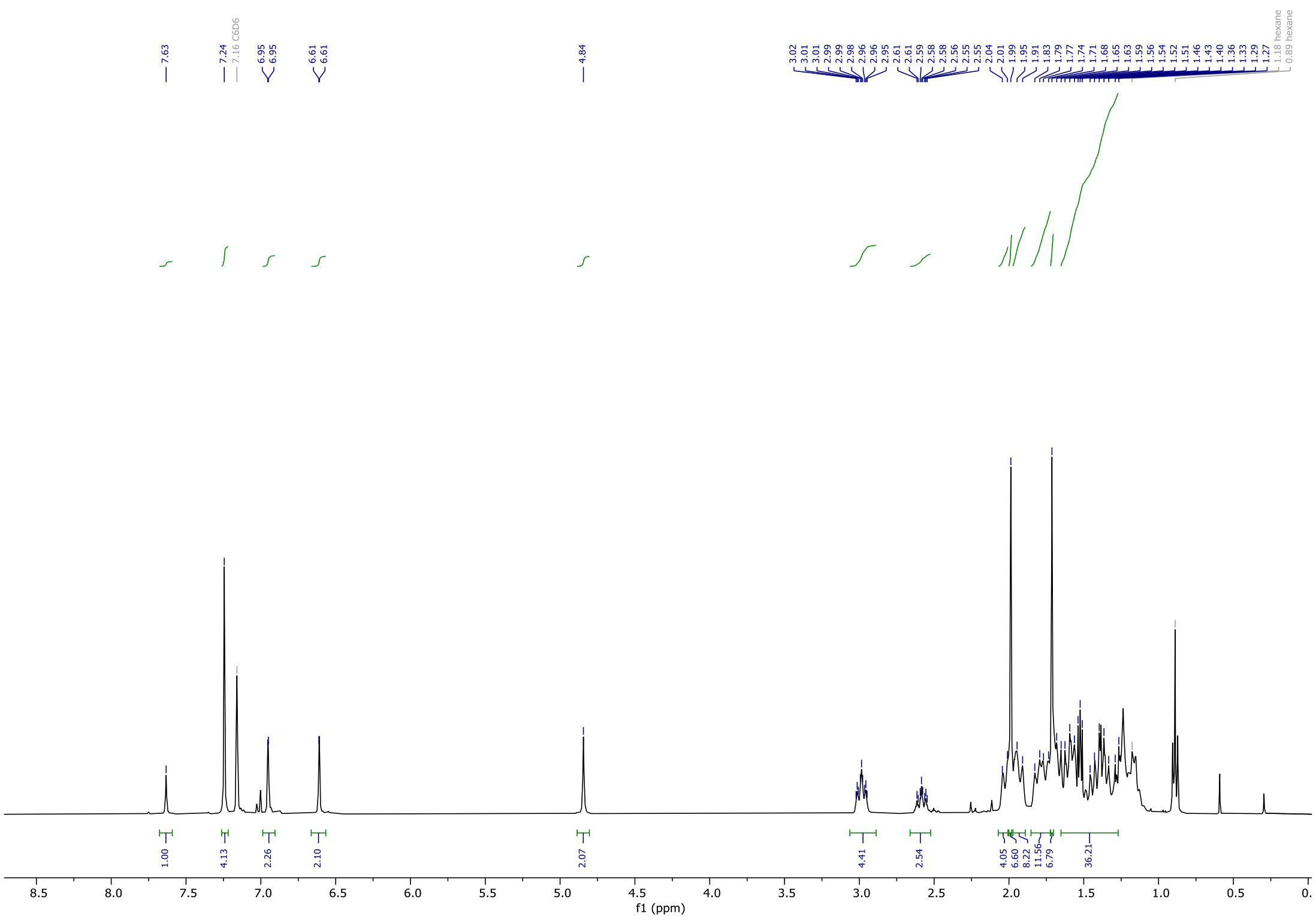

Figure S1: ^{1}H NMR spectrum (600 MHz, C_6D_6) of (NNN^{TCHP})H$_3$.

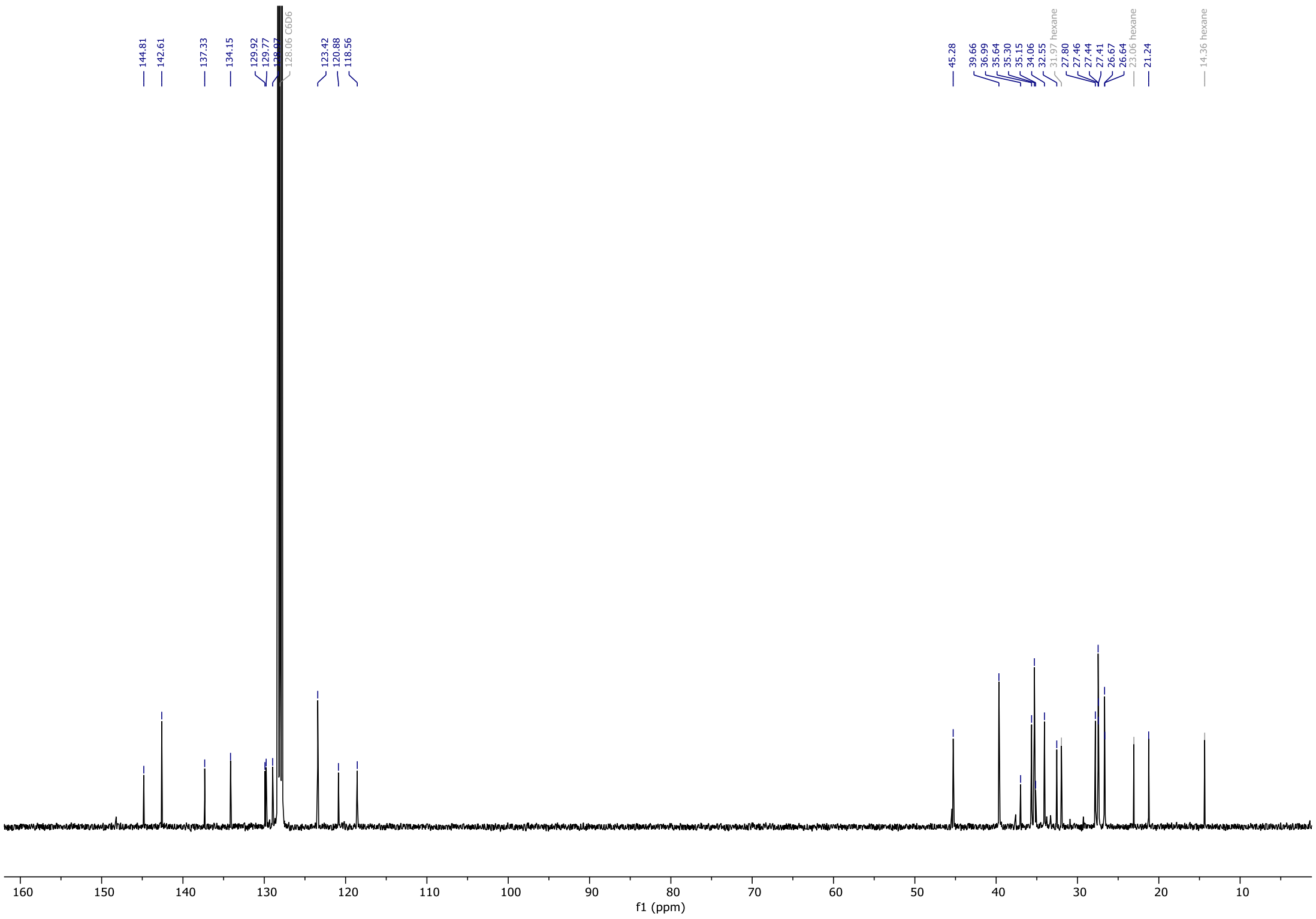

Figure S2: $^{13}C\{^{1}H\}$ NMR spectrum (151 MHz, C_6D_6) of $(NNN^{TCHP})H_3$.

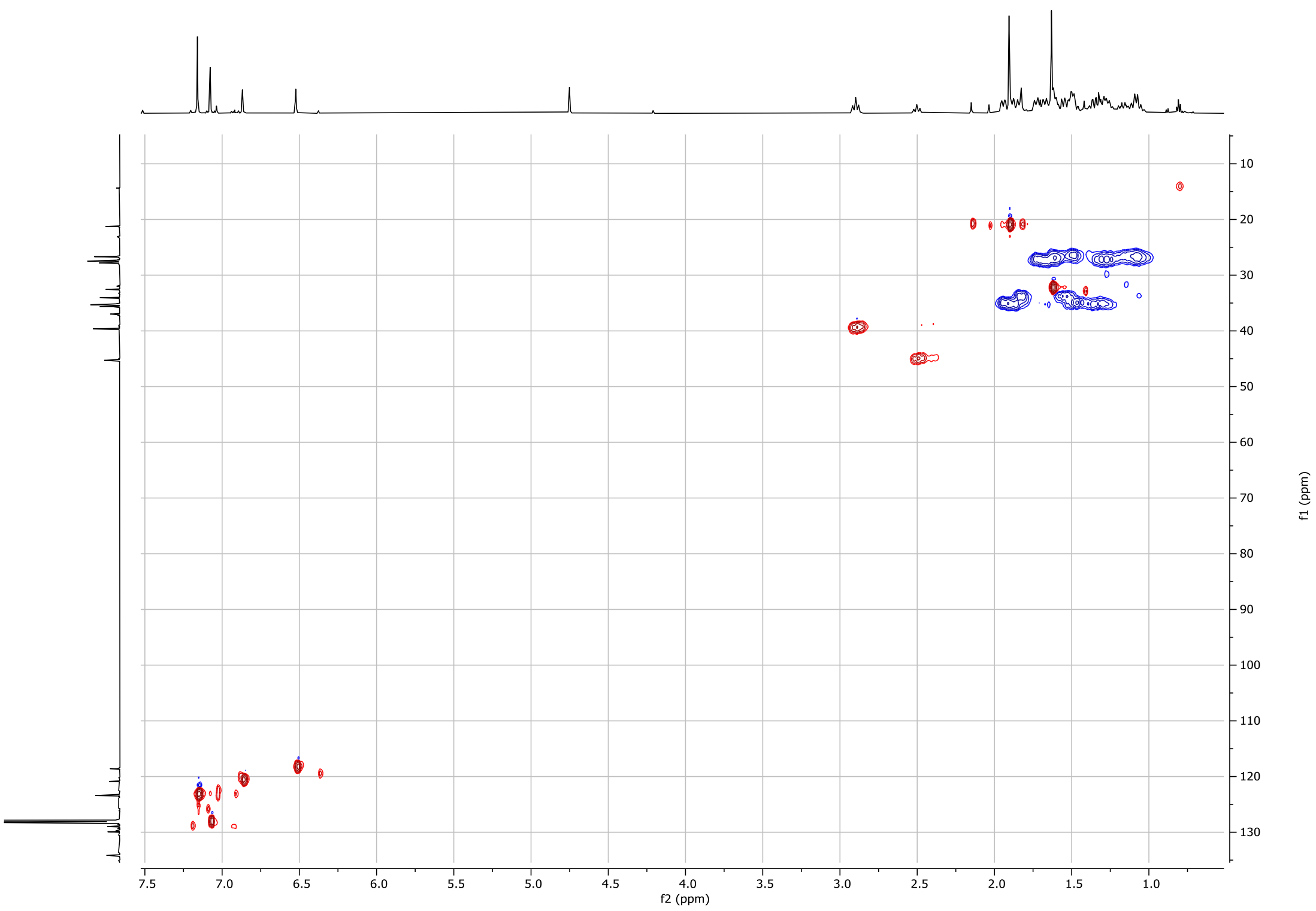

Figure S3: HSQC NMR spectrum (C_6D_6) of $(NNN^{TCHP})H_3$.

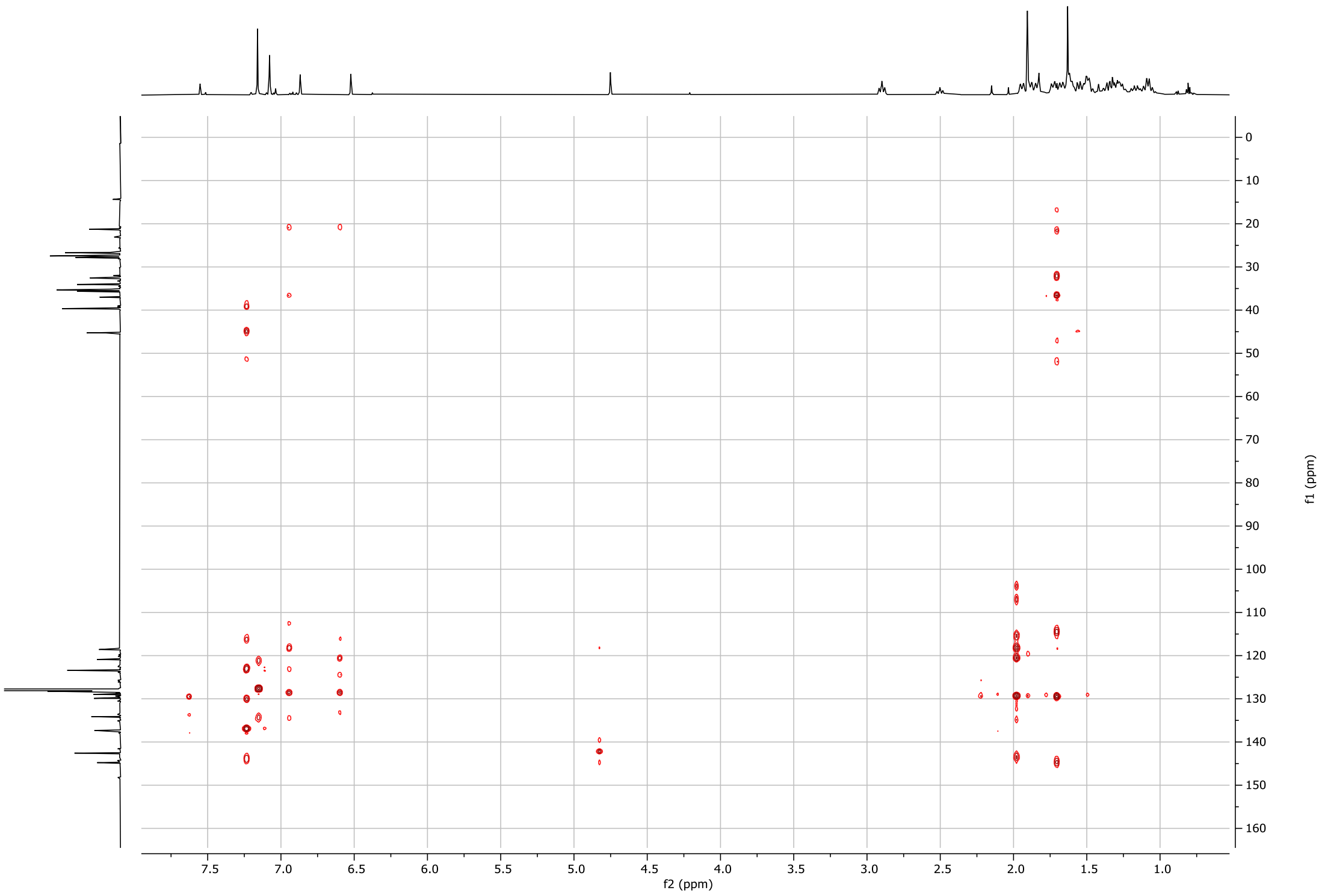

Figure S4: HMBC NMR spectrum (C_6D_6) of $(NNN^{TCHP})H_3$.

Preparation of 1,8-bis(2,6-dicyclohexylanilino)-3,6,9,9-tetramethyl-9,10-dihydroacridine, $(NNN^{DCHP})H_3$

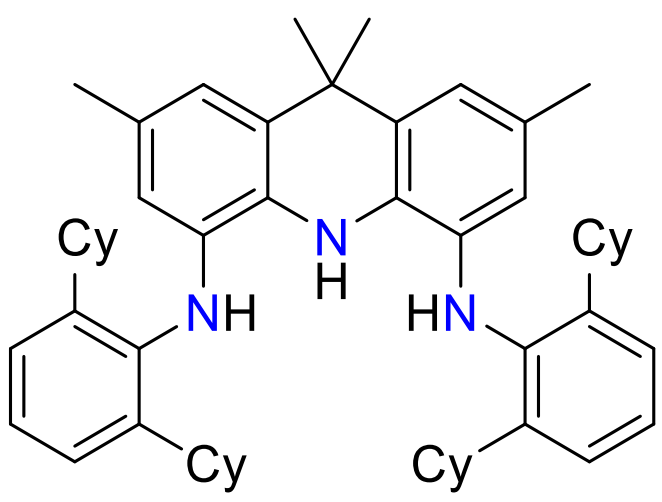

1,8-Dibromo-3,6,9,9-tetramethyl-9,10-dihydroacridine (897 mg, 2.3 mmol), 2,6-dicyclohexylaniline (1.17 g, 4.6 mmol), sodium *tert*-butoxide (652 mg, 15.2 mmol) and bis(tri-*tert*-butylphosphine) palladium(0) (20 mg, 0.04 mmol, 2 mol%) were charged to a flame dried J Young fitted Schlenk flask. Toluene (50 mL) was added to the combined solid masses and the flask heated to 120 °C for 4 days (under an atmosphere of N_2). The mixture was quenched with degassed water, extracted with toluene (2 x 50 mL) and dried over anhydrous magnesium sulphate. The volatiles were removed *in vacuo* and to the residue was added dry acetonitrile (50 mL). The mixture was sonicated for 1 hour and the product obtained by filtration as a pale-

yellow solid that was determined to be of high purity by multinuclear NMR spectroscopic measurements. **Yield**: 1.18 g, 69 %.

m.p.: 254 – 256 °C (dec).

^{1}H NMR (400 MHz, C_6D_6): δ 1.04 – 1.20 (m, 10H, Cy–*H*), 1.25 – 1.58 (m, 16H, Cy–*H*), 1.60 – 1.75 (m, 6H, Cy–*H*), 1.70 (s, 6H, C(C*H*$_3$)$_2$), 1.77 – 1.97 (m, 8H, Cy–*H*), 2.04 (s, 6H, C*H*$_3$), 2.85 – 2.97 (m, 4H, Cy–*H*), 4.85 (s, 2H, DCHPN*H*), 6.56 (s, 2H, ACRAr–*H*), 6.96 (s, 2H, ACRAr–*H*), 7.20 – 7.24 (m, 6H, DCHPAr–*H*), 7.52 (s, 1H, ACRN*H*).

^{13}C{^{1}H} NMR (101 MHz, C_6D_6): 21.3 (*C*H$_3$), 26.6, 27.3, 27.7 (Cy–*C*), 32.4 (C(*C*H$_3$)$_2$), 33.9, 35.3 (Cy–*C*), 37.0 (*C*(CH$_3$)$_2$), 39.4 (Cy–*C*), 118.9, 121.0, 125.1, 125.4, 129.1, 129.7, 129.9, 133.8, 139.5, 142.5 (ACRAr–*C*, DCHPAr–*C*).

I.R. (solid, cm^{-1}): 3405 (w), 3362 (w), 2919 (s), 2846 (s), 1606 (m), 1498 (s), 1463 (w), 1444 (s), 1388 (w), 1359 (w), 1310 (w), 1284 (m), 1271 (m), 1247 (m), 1191 (w), 1176 (w), 1131 (w), 1094 (w), 1027 (m), 945 (w), 861 (m), 840 (m), 816 (w), 798 (m), 766 (s), 751 (s), 699 (s), 647 (m).

Note: Darkens under aerobic conditions and is thus stored under an inert atmosphere in a glovebox.

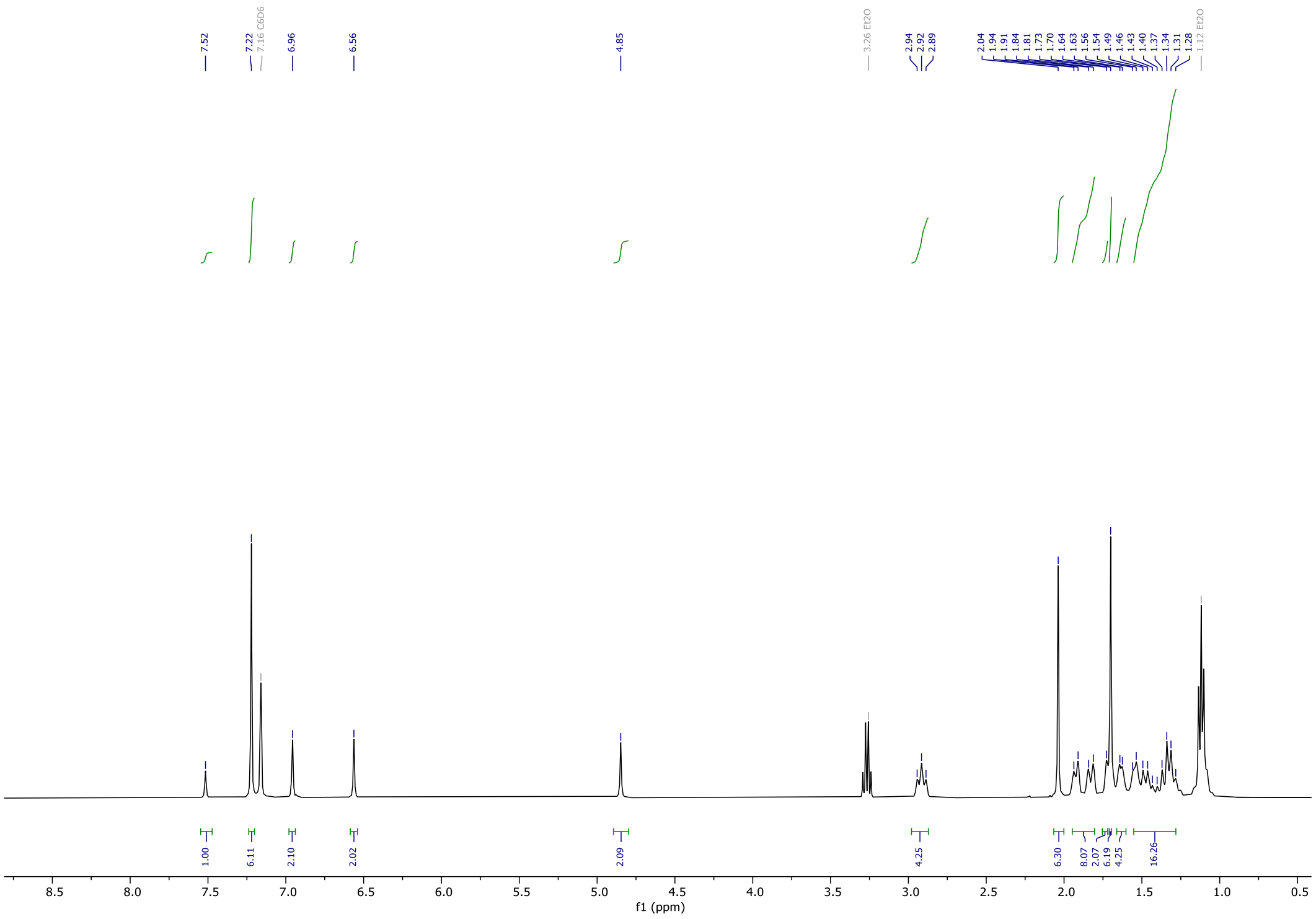

Figure S5: ^{1}H NMR spectrum (400 MHz, C_6D_6) of (NNN^{DCHP})H_3.

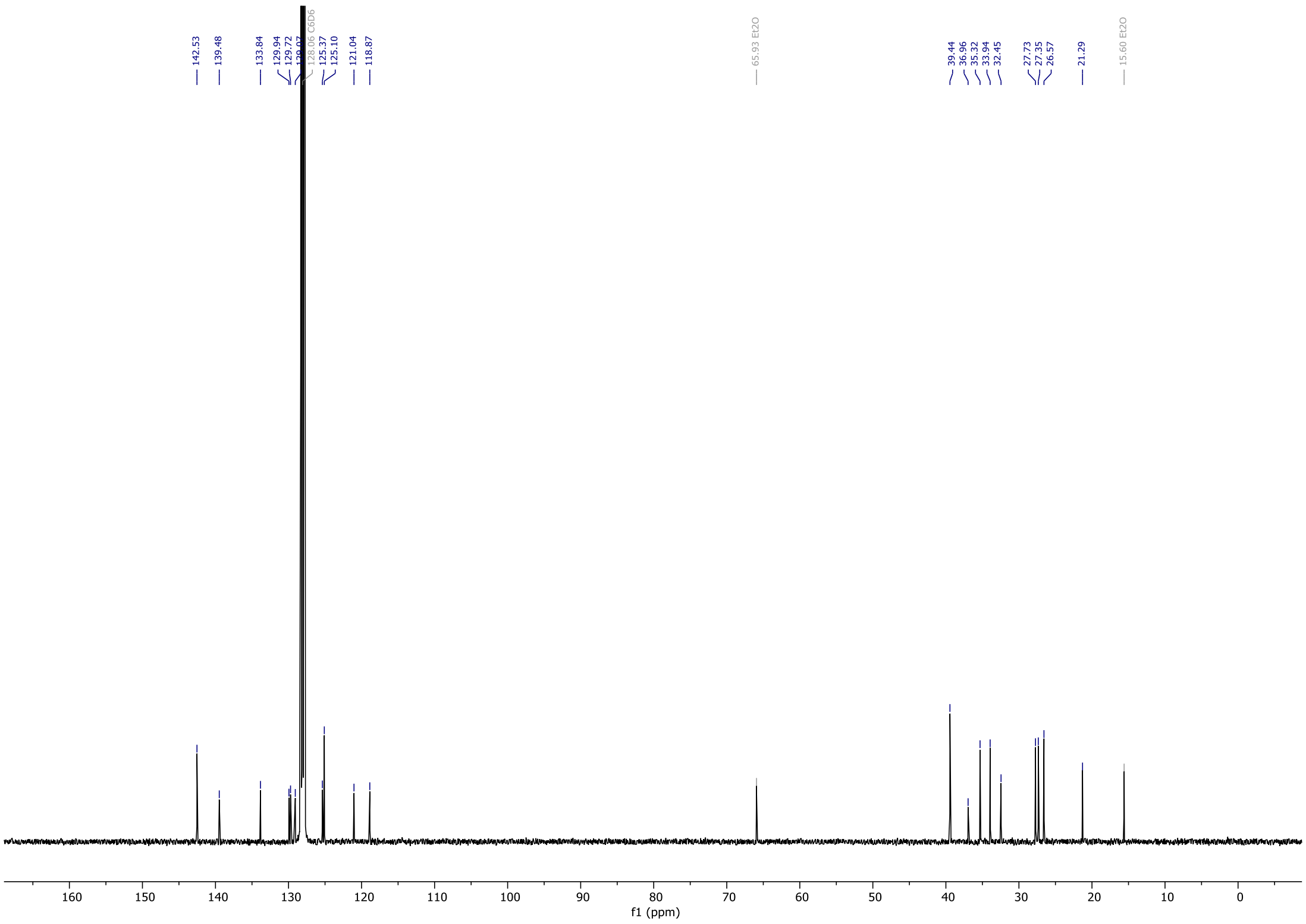

Figure S6: $^{13}C\{^{1}H\}$ NMR spectrum (101 MHz, C_6D_6) of $(NNN^{DCHP})H_3$.

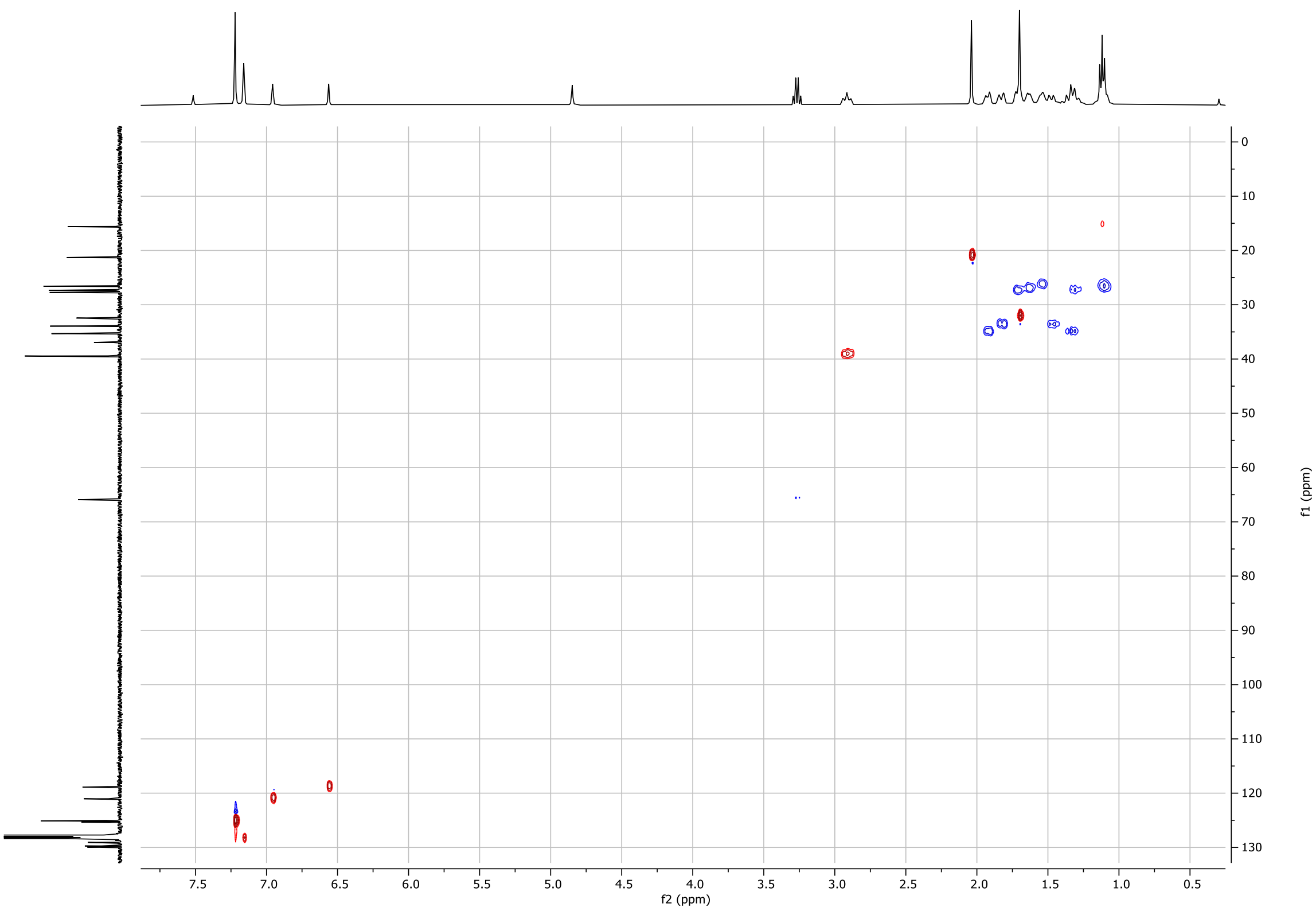

Figure S7: HSQC NMR spectrum (C_6D_6) of $(NNN^{DCHP})H_3$.

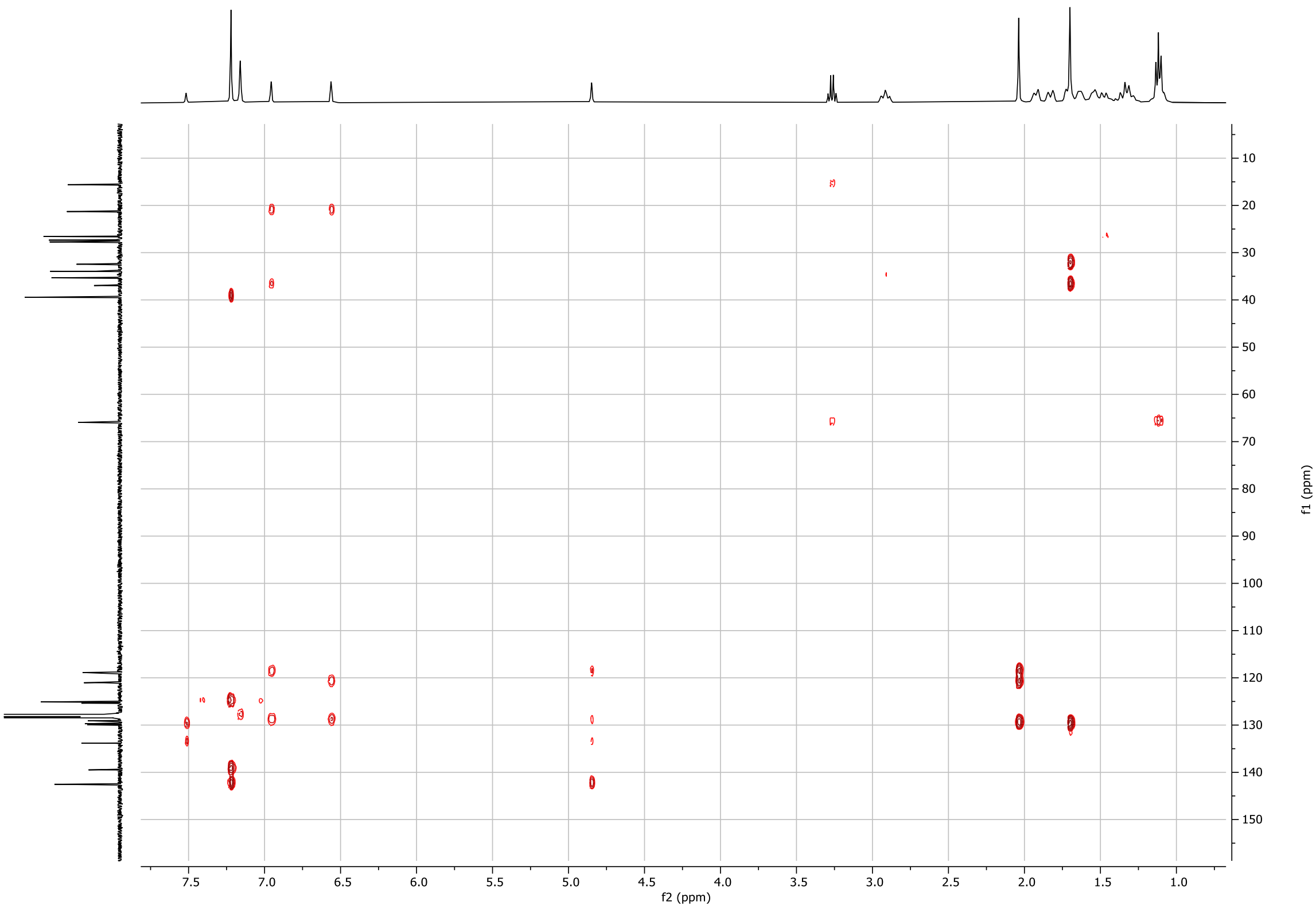

Figure S8: HMBC NMR spectrum (C_6D_6) of (NNN^{DCHP})H_3.

Preparation of [Al_2Me_3(NNN^{TCHP})] (1)

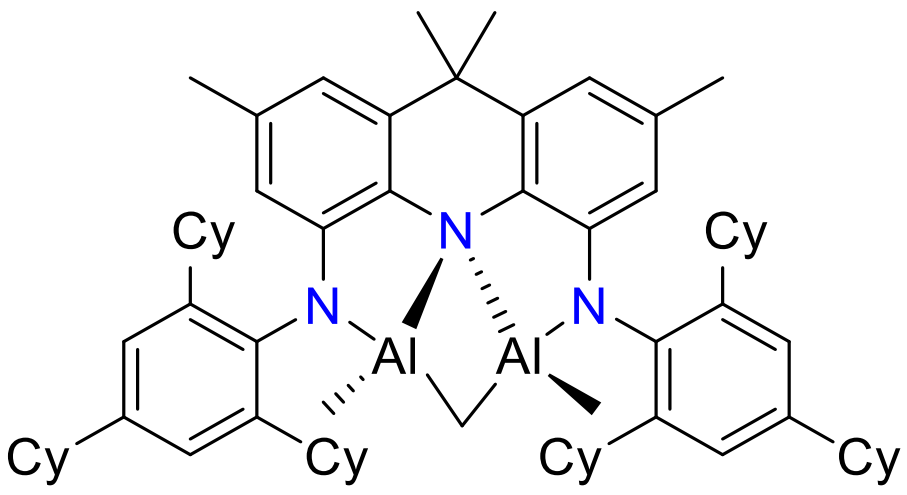

(NNN^{TCHP})H_3 (500 mg, 0.55 mmol) was charged to a J Young fitted Schlenk flask and benzene was added (~10 mL). Trimethylaluminium (0.55 mL, 1.09 mmol, 2M in toluene) was added to the reaction mixture at room temperature and allowed to stir for 30 minutes. The vessel was sealed (vented periodically to avoid over pressure) and heated at 80 °C for 18 hours to give a deep violet solution. The volatiles were removed *in vacuo* and hexane (~5 mL) was added. The mixture was allowed to sit at room temperature for 18 hours to give the title compound as an off-white microcrystalline solid. **Yield**: 254 mg, 46 %.

m.p.: > 300 °C (dec).

^{1}H NMR (600 MHz, C_6D_6): δ -0.28 (s, 6H, C$\underline{H}_3$), 0.54 (s, 3H, C$\underline{H}_3$), 1.19 – 1.51 (m, 24H, Cy–$\underline{H}$), 1.53 – 1.73 (m, 16H, Cy–$\underline{H}$), 1.75 (s, 6H, C(C$\underline{H}_3$)$_2$), 1.77 – 1.88 (m, 14H, Cy–$\underline{H}$), 1.92 (s, 6H, $_{ACR}$Ar–C$\underline{H}_3$), 2.02 – 2.10 (m, 4H, Cy–$\underline{H}$), 2.19 – 2.29 (m, 2H, Cy–$\underline{H}$), 2.58 – 2.68 (m, 4H, Cy–$\underline{H}$), 3.29 – 3.40 (m, 2H, Cy–$\underline{H}$), 5.87 (d, J = 1.8 Hz, 2H, $_{ACR}$Ar–$\underline{H}$), 6.53 (d, J = 1.8 Hz, 2H, $_{ACR}$Ar–$\underline{H}$), 7.30 (d, J = 2.2 Hz, 2H, $_{TCHP}$Ar–$\underline{H}$), 7.38 (d, J = 2.2 Hz, 2H, $_{TCHP}$Ar–$\underline{H}$).

^{13}C{^{1}H} NMR (151 MHz, C_6D_6): δ -9.8, -3.4 ($\underline{C}$H$_3$), 21.3 ($_{ACR}$Ar–$\underline{C}$H$_3$), 26.6, 26.7, 26.7, 27.1, 27.2, 27.4, 27.4, 27.8 (Cy–$\underline{C}$), 34.5 (C($\underline{C}$H$_3$)$_2$), 34.7, 34.7, 35.2, 36.8, 37.3, 37.5 (Cy–$\underline{C}$), 37.9 ($\underline{C}$(CH$_3$)$_2$), 40.0, 45.2 (Cy–$\underline{C}$), 110.3, 114.7, 124.1, 124.6, 125.5, 133.4, 133.7, 136.9, 145.7, 146.0, 147.3, 147.8 ($_{ACR}$Ar–$\underline{C}$, $_{TCHP}$Ar–$\underline{C}$).

I.R. (solid, cm^{-1}): 743 (w), 839 (m), 861 (m), 921 (w), 949 (w), 997 (w), 1031 (m), 1090 (w), 1134 (w), 1157 (w), 1196 (m), 1238 (w), 1293 (w), 1336 (w), 1447 (s), 1457 (w), 1497 (w), 1508 (w), 1560 (w), 1592 (w), 1592 (m), 1605 (m), 2850 (s), 2919 (s).

Anal. Calcd. for $C_{68}H_{95}Al_2N_3$ (1008.49): C, 80.99; H, 9.50; N, 4.17 %. Found: C, 79.62; H, 9.48; N, 3.57 %.

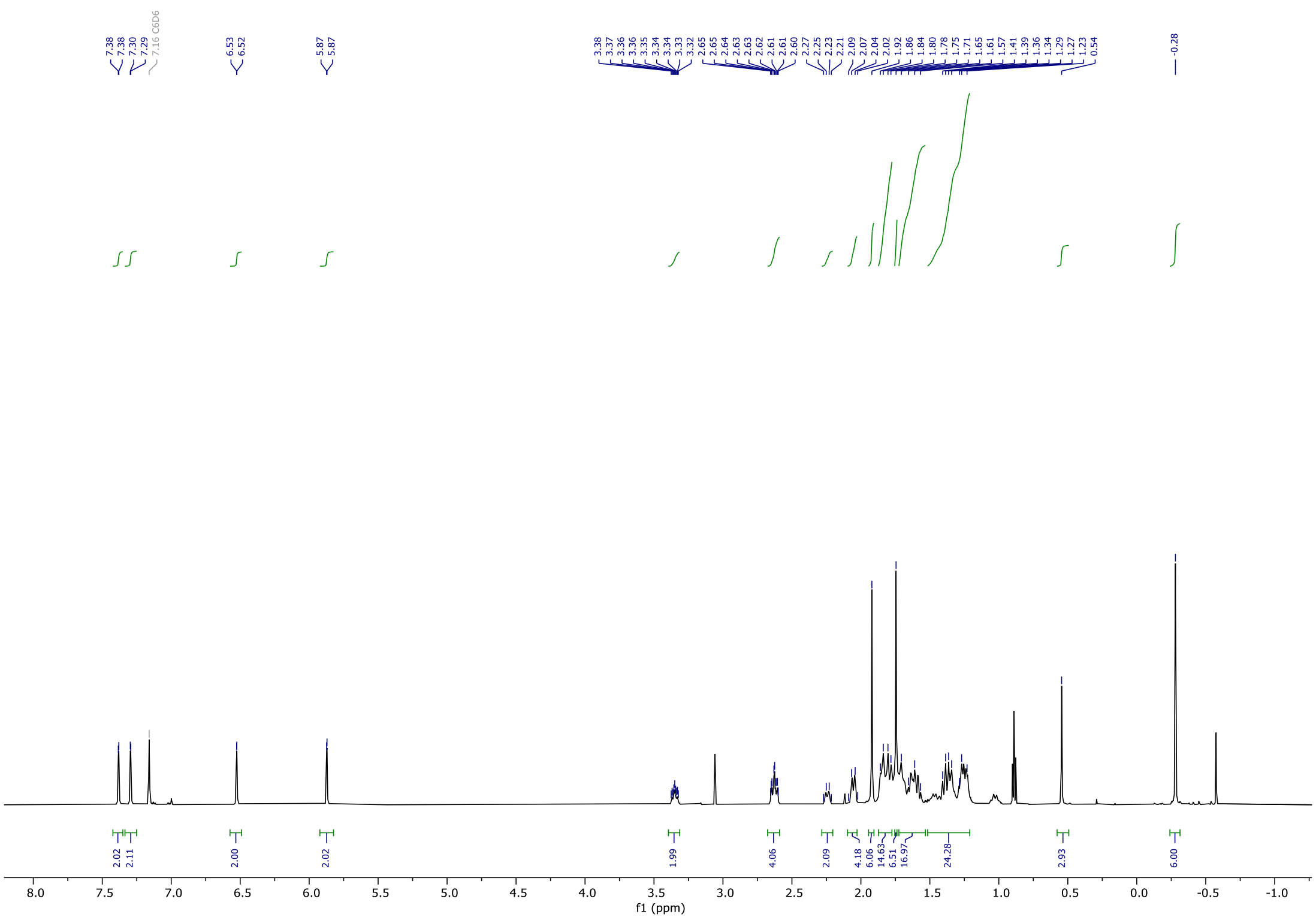

Figure S9: ^{1}H NMR spectrum (600 MHz, C_6D_6) of **1**.

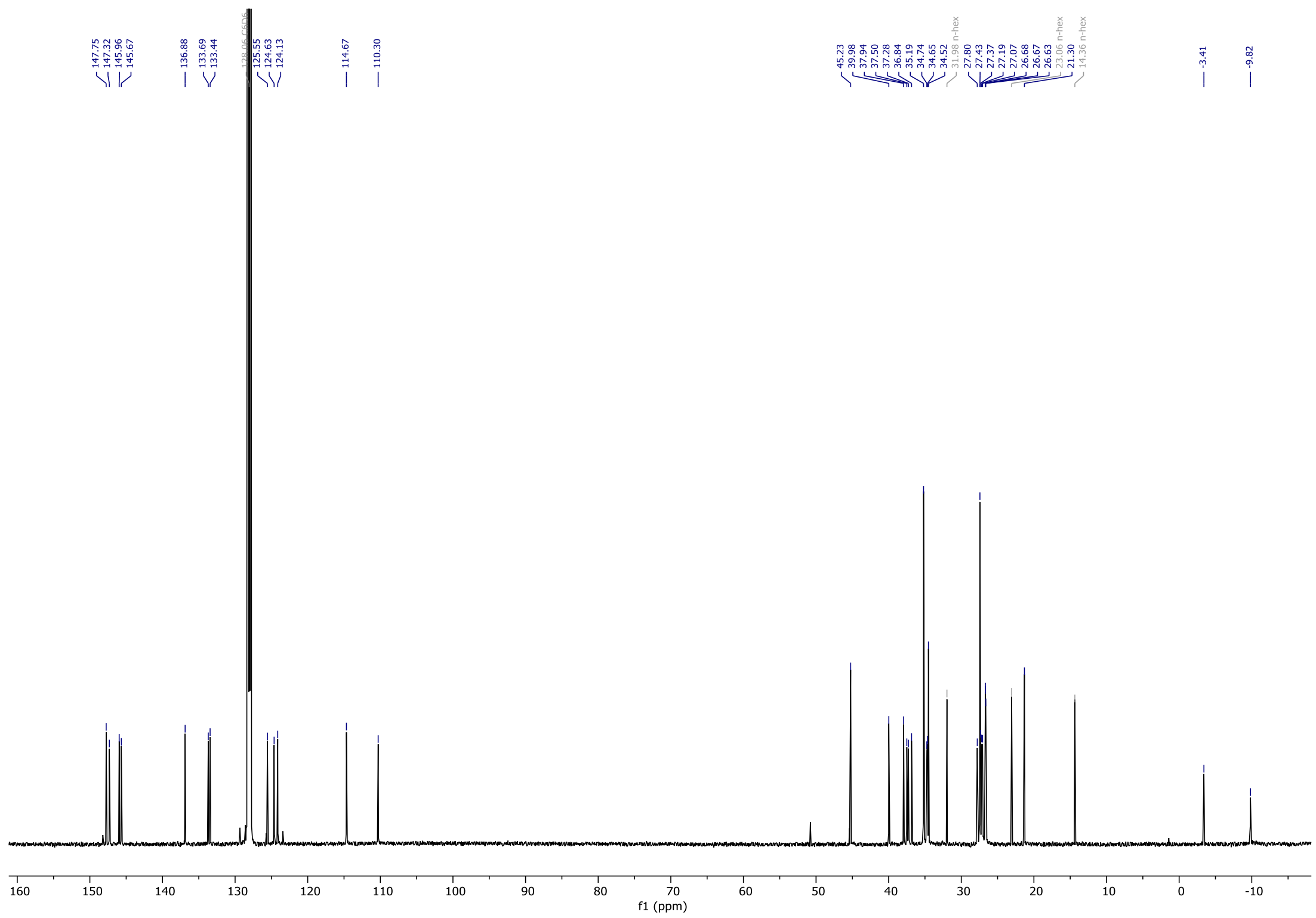

Figure S10: $^{13}C\{^1H\}$ NMR spectrum (151 MHz, C_6D_6) of **1**.

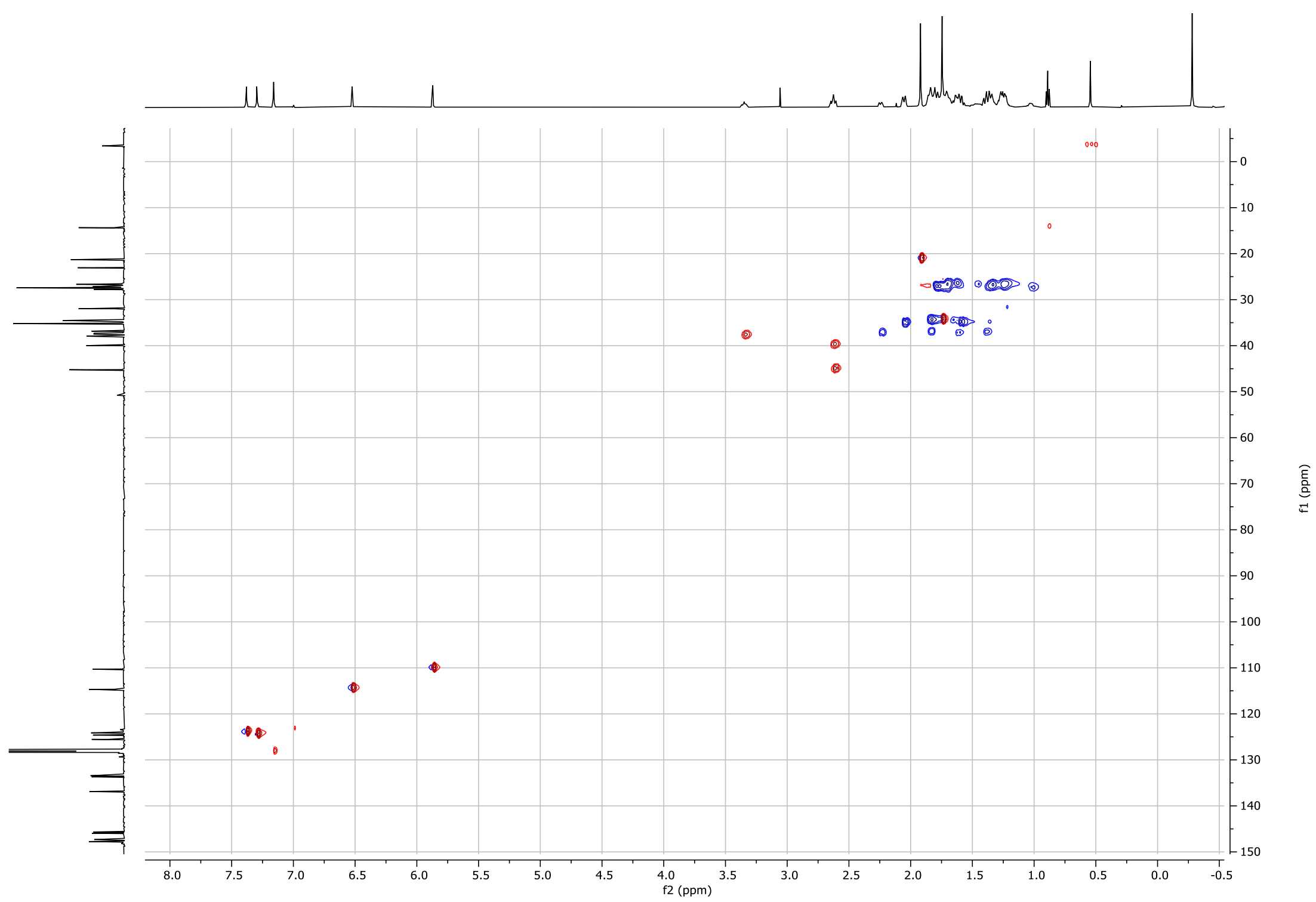

Figure S11: HSQC NMR spectrum (C_6D_6) of **1**.

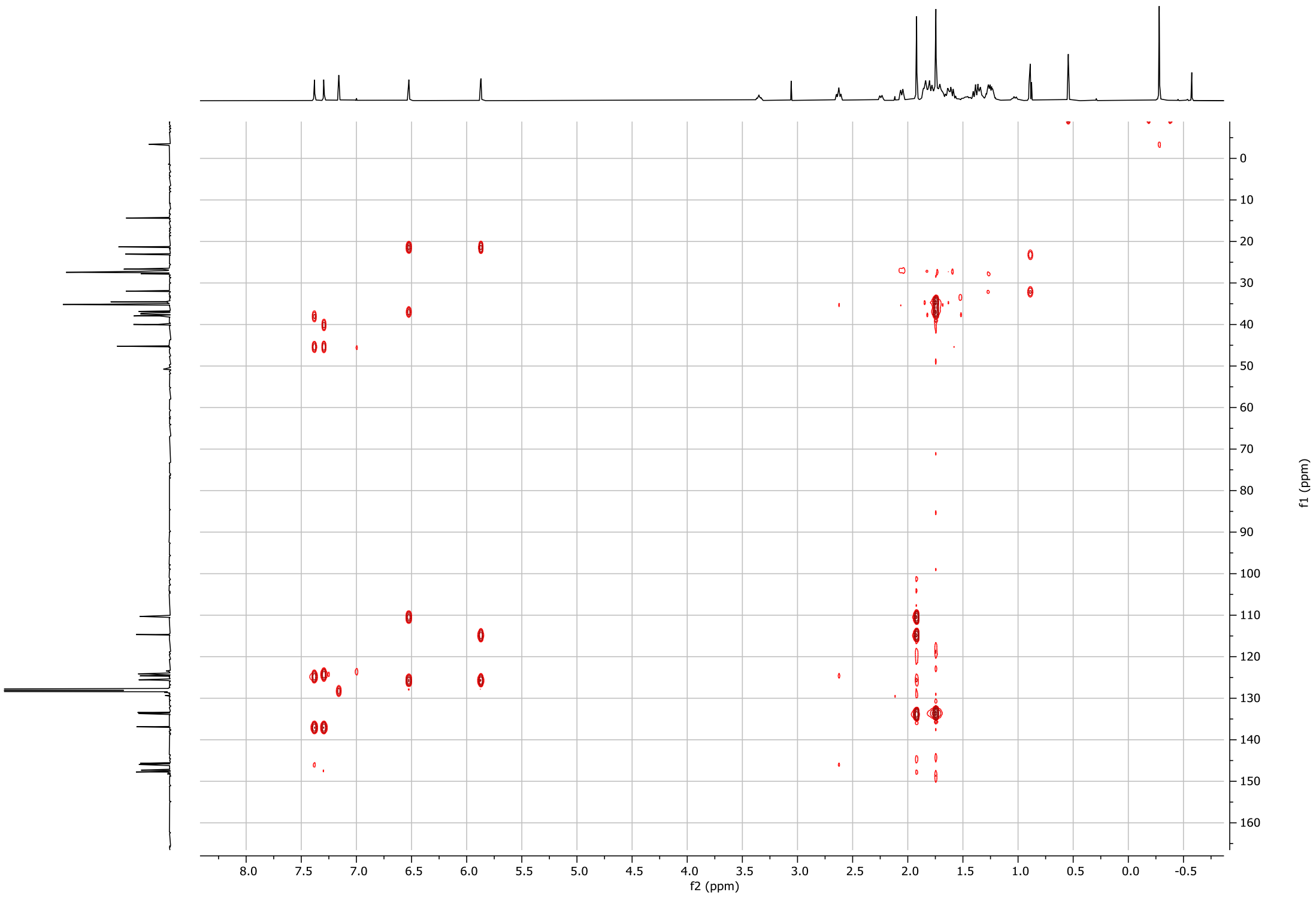

Figure S12: HMBC NMR spectrum (C_6D_6) of **1**.

Preparation of [Al(NNN^{TCHP})(I)$_2$] (2)

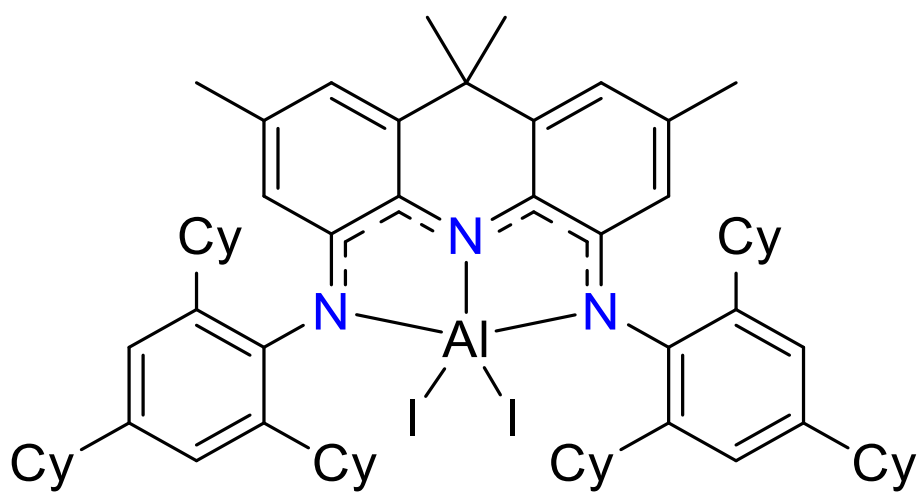

(NNN^{TCHP})H_3 (250 mg, 0.27 mmol) was charged to a J Young fitted Schlenk flask and benzene was added (~10 mL). Trimethylaluminium (0.27 mL, 0.55 mmol, 2M in toluene) was added to the reaction mixture at room temperature and allowed to stir for 30 minutes. The vessel was sealed (vented periodically to avoid over pressure) and heated at 80 °C for 18 hours to give a deep violet solution. The reaction mixture was cooled to room temperature and iodine (278 mg, 1.09 mmol) was added in one portion. The vessel was sealed and allowed to stir at room temperature for 72 hours to give a dark brown solution. The volatiles were removed *in vacuo* and pentane was added (~5 mL) resulting in the deposition of the title compound as a dark brown solid. The solid was collected by filtration and dried under high vacuum. Crystals were

isolated from a saturated pentane solution (supernatant) stored at –30 °C for 72 hours. **Yield**: 241 mg, 74 %.

m.p.: 216 – 218 °C (dec).

^{1}H NMR (400 MHz, C_6D_6): δ 1.19 (s, 6H, C(C*H*$_3$)$_2$), 1.22 – 1.38 (m, 10H, Cy–*H*), 1.42 (s, 6H, C*H*$_3$), 1.44 – 2.02 (m, 48H, Cy–*H*), 2.44 – 2.62 (m, 6H, Cy–*H*), 3.42 – 3.48 (m, 4H, Cy–*H*), 6.00 (s, 2H, ACRAr–*H*), 6.10 (s, 2H, ACRAr–*H*), 7.35 (s, 4H, TCHPAr–*H*).

^{13}C{^{1}H} NMR (101 MHz, C_6D_6): δ 22.4 (*C*H$_3$), 26.6, 26.9, 27.1, 27.4, 27.6 (Cy–*C*), 32.2 (C(*C*H$_3$)$_2$), 34.2, 35.0, 37.0 (Cy–*C*), 38.1 (*C*(CH$_3$)$_2$), 40.0, 44.9 (Cy–*C*), 118.0, 123.8, 128.6, 130.9, 139.4, 139.7, 143.7, 146.5, 148.0, 158.4 (ACRAr–*C*, TCHPAr–*C*).

I.R. (solid, cm^{-1}): 2919 (s), 2846 (s), 1605 (m), 1544 (w), 1498 (m), 1444 (m), 1418 (m), 1370 (w), 1273 (s), 1252 (s), 1159 (w), 1131 (w), 1073 (w), 1027 (m), 997 (m), 949 (w), 915 (m), 895 (m), 859 (m), 842 (m), 831 (m), 779 (w), 725 (w), 673 (m).

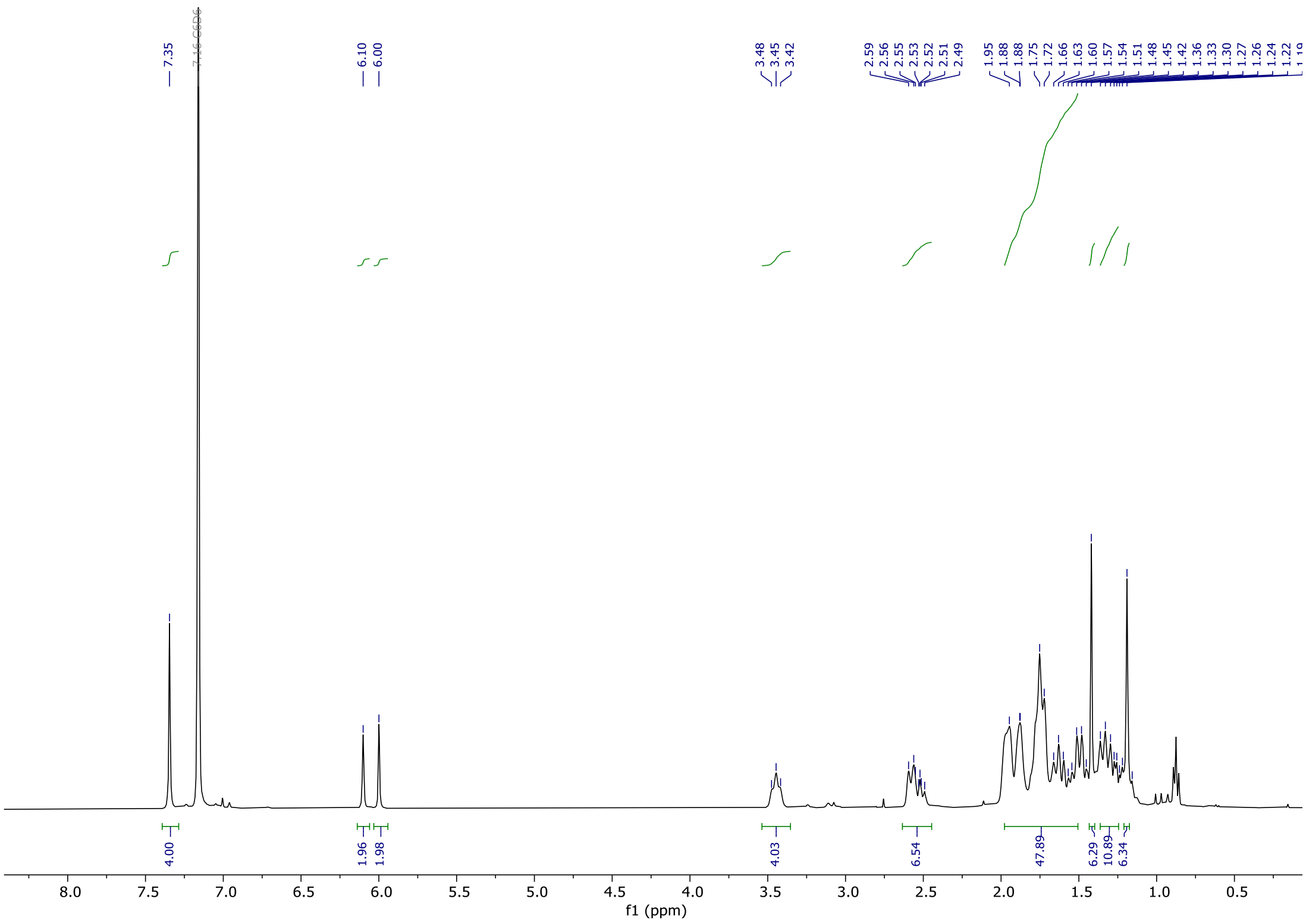

Figure S13: ^{1}H NMR spectrum (400 MHz, C_6D_6) of **2**.

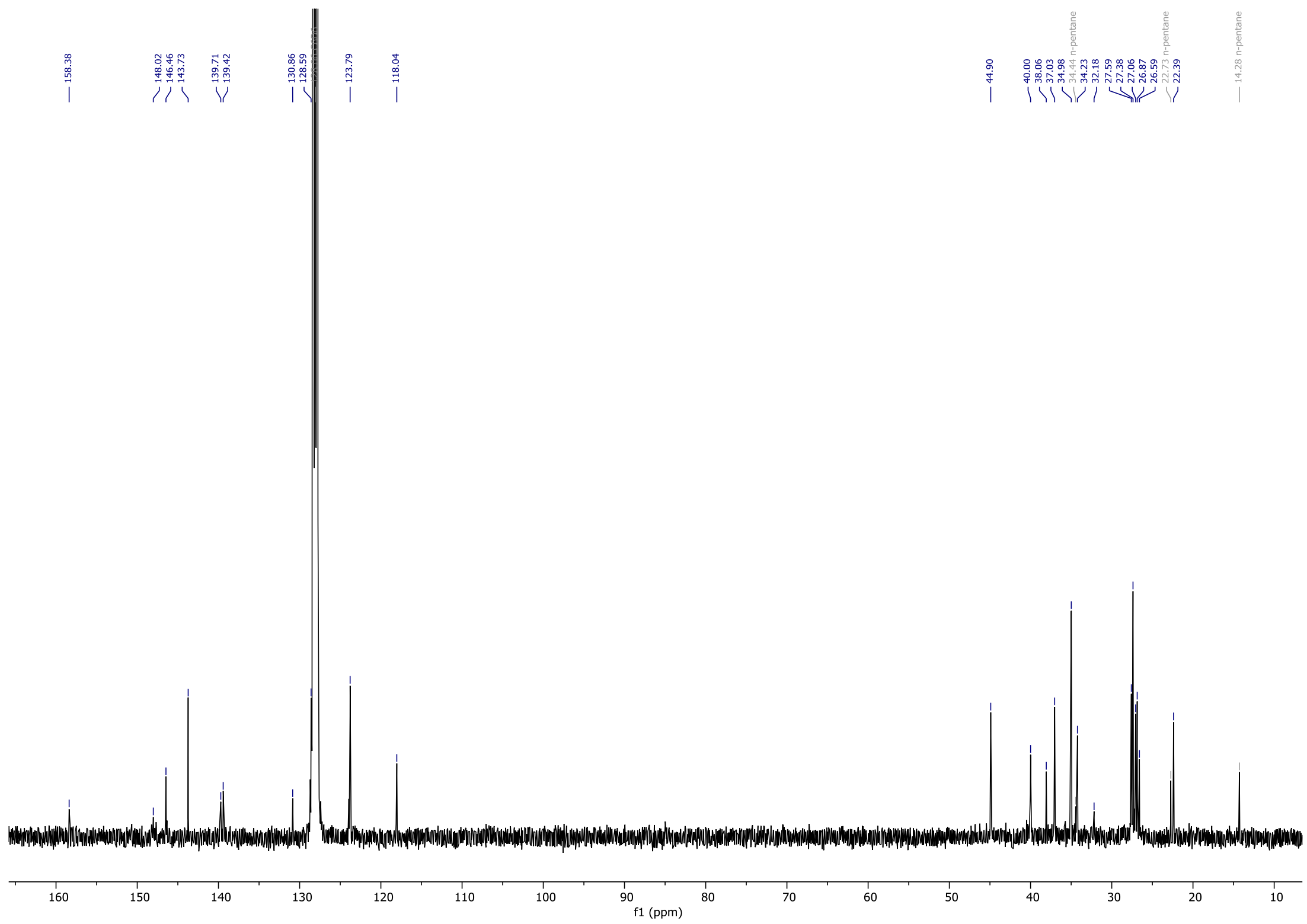

Figure S14: $^{13}C\{^{1}H\}$ NMR spectrum (101 MHz, C_6D_6) of **2**.

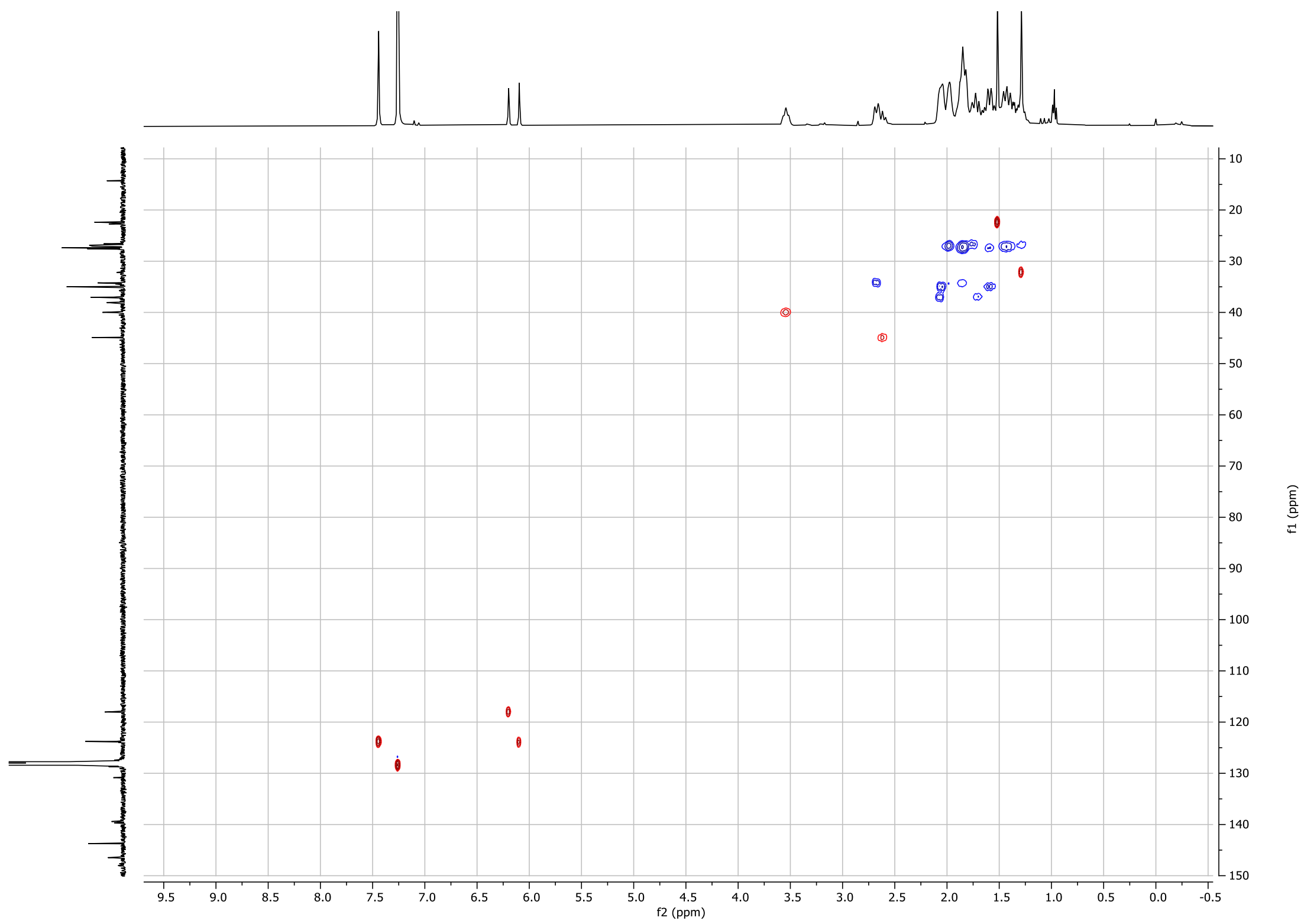

Figure S15: HSQC NMR spectrum (C_6D_6) of **2**.

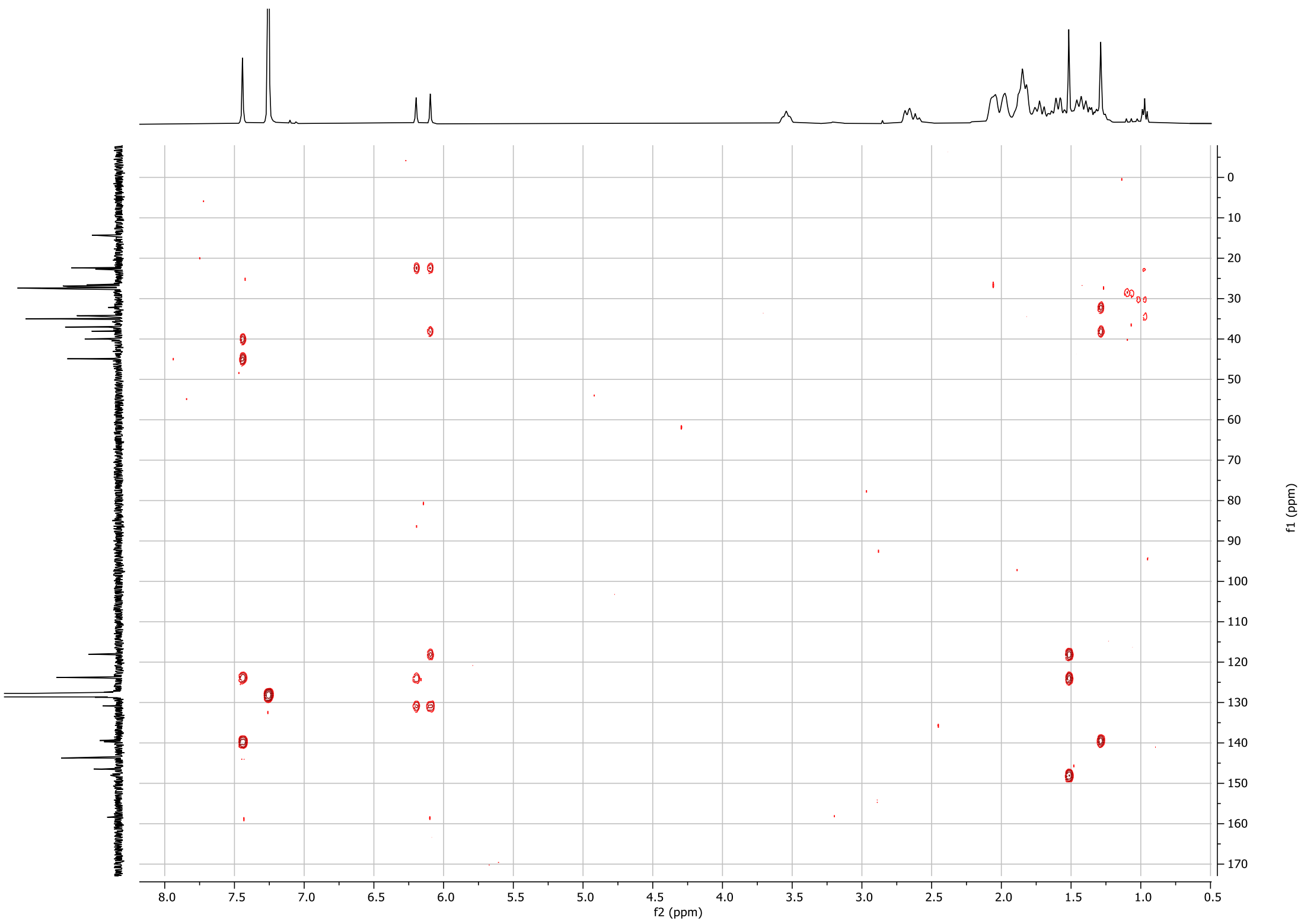

Figure S16: HMBC NMR spectrum (C_6D_6) of **2**.

Preparation of [K(η^2-C_6H_6)][Al(NNN^{TCHP})(I)] (3)

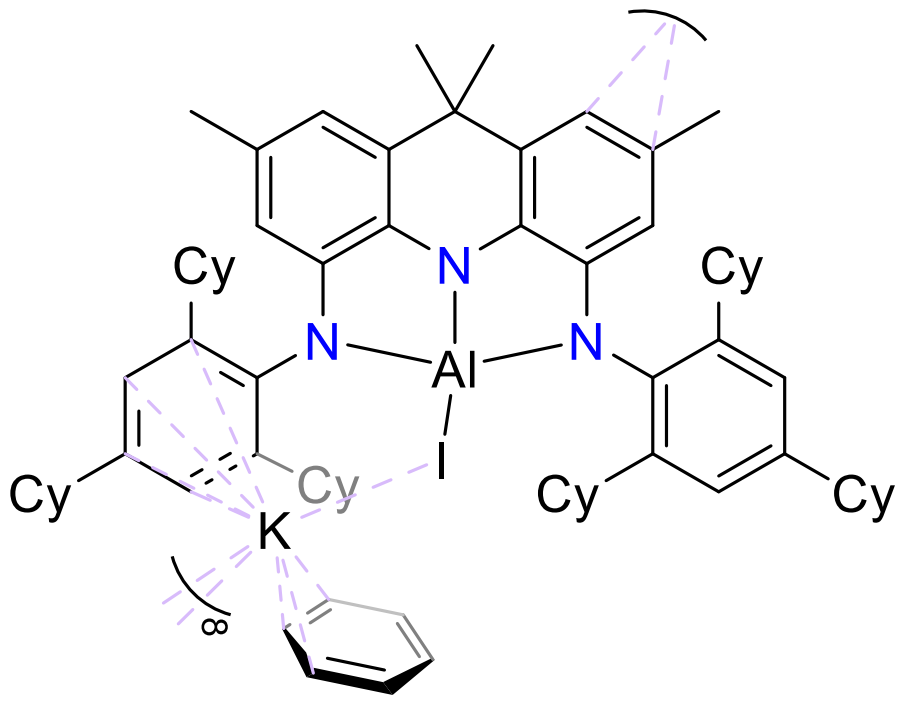

Al(NNN^{TCHP})(I)$_2$ (200 mg, 0.17 mmol) and 5% w/w K/KI (655 mg, 0.84 mmol) were charged to a Schlenk flask and benzene was added (~10 mL). The reaction mixture was allowed to stir at room temperature for 18 hours to give a yellow-red solution. The solution was filtered *via.* cannula and the volatiles reduced *in vacuo* (to ~1 mL). A small number of colourless crystals suitable for single crystal X-ray diffraction measurements were grown at room temperature over 72 hours. Insufficient material was obtained for further characterisation.

Preparation of [Al(NNN^{TCHP})(TMEDA)] (4)

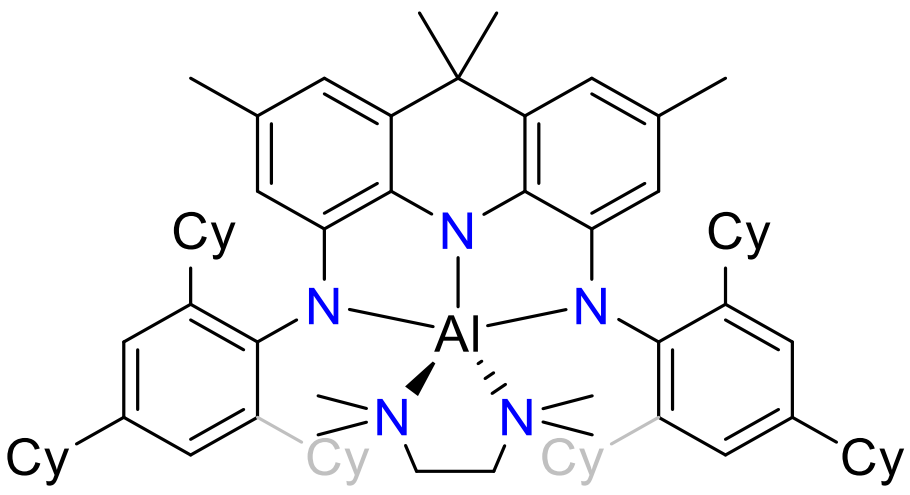

[Al(NNN^{TCHP})(I)$_2$] (33 mg, 0.028 mmol), KC_8 (20 mg, 0.15 mmol) and tetramethylethylenediamine (0.10 mL, 0.67 mmol) were charged to a Schlenk flask and benzene was added (~1 mL). The reaction mixture was allowed to stir at room temperature for 18 hours to give a yellow solution. The solution was filtered *via.* cannula and the volatiles removed *in vacuo* to give an oily residue. The residue was suspended in hexane to give a colourless solid, the supernatant decanted, and the solid dried under high vacuum. Colourless crystals could be obtained from the storage of a saturated benzene solution of **4** at room temperature for 72 hours. **Yield**: 19 mg, 64 %.

m.p.: 250 – 256 °C (dec).

^{1}H NMR (400 MHz, C_6D_6): δ 1.31 – 1.48 (m, 12H, Cy–*H*), 1.56 (br s, 12H, TMEDA–C*H*$_3$), 1.60 – 1.82 (m, 26H, Cy–*H*), 1.83 (s, 3H, C(C*H*$_3$)$_2$), 1.87 – 2.05 (m, 20H, Cy–*H*), 2.12 (s, 3H, C(C*H*$_3$)$_2$), 2.24 (s, 6H, C*H*$_3$), 2.37 (br s, 4H, TMEDA–C*H*$_2$), 2.40 – 2.45 (m, 2H, Cy–*H*), 2.51 – 2.59 (m, 2H, Cy–*H*), 2.75 – 2.82 (m, 2H, Cy–*H*), 3.16 – 3.27 (m, 2H, Cy–*H*), 6.08 (s, 2H, $_{\text{ACR}}$Ar–*H*), 6.79 (s, 2H, $_{\text{ACR}}$Ar–*H*), 7.20 (s, 2H, $_{\text{TCHP}}$Ar–*H*), 7.25 (s, 2H, $_{\text{TCHP}}$Ar–*H*).

^{13}C{^{1}H} NMR (101 MHz, C_6D_6): δ 22.5 (*C*H$_3$), 24.0 (C(*C*H$_3$)$_2$), 26.0, 26.2, 26.7, 27.0, 27.0, 27.4, 27.4, 27.6, 28.1 (Cy–*C*), 30.2 (C(*C*H$_3$)$_2$), 34.9, 35.2, 35.3, 35.4, 37.5 (Cy–*C*), 37.8 (*C*(CH$_3$)$_2$), 37.9, 38.0, 39.7, 44.7 (Cy–*C*), 46.3 (TMEDA–*C*H$_3$), 57.1 (TMEDA–*C*H$_2$), 112.4, 115.3, 123.5, 124.4, 126.5, 129.0, 136.9, 143.3, 145.6, 147.5, 147.8, 149.2 ($_{\text{ACR}}$Ar–*C*, $_{\text{TCHP}}$Ar–*C*).

I.R. (solid, cm^{-1}): 2921 (s), 2848 (s), 1588 (w), 1561 (w), 1473 (s), 1446 (s), 1346 (m), 1300 (w), 1261 (s), 1227 (w), 1200 (w), 1150 (w), 1100 (m), 1016 (s), 952 (w), 896 (w), 858 (w), 800 (s), 680 (w).

Anal. Calcd. for $C_{71}H_{102}AlN_5$•($C_6H_{16}N_2$) (1168.82): C, 79.13; H, 10.18; N, 8.39 %. Found: C, 78.25; H, 10.11; N, 6.99 %.

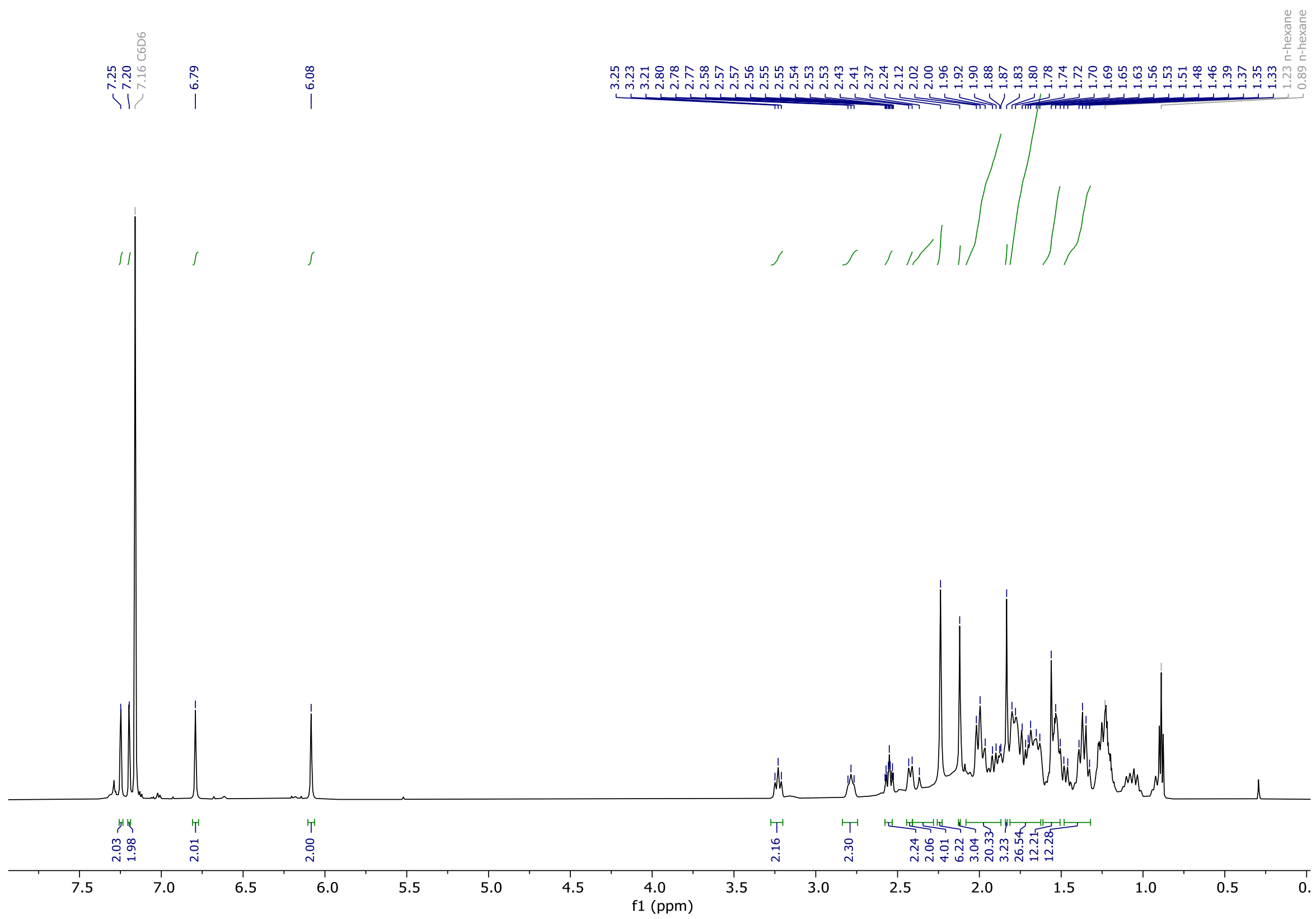

Figure S17: ^{1}H NMR spectrum (400 MHz, C_6D_6) of **4**.

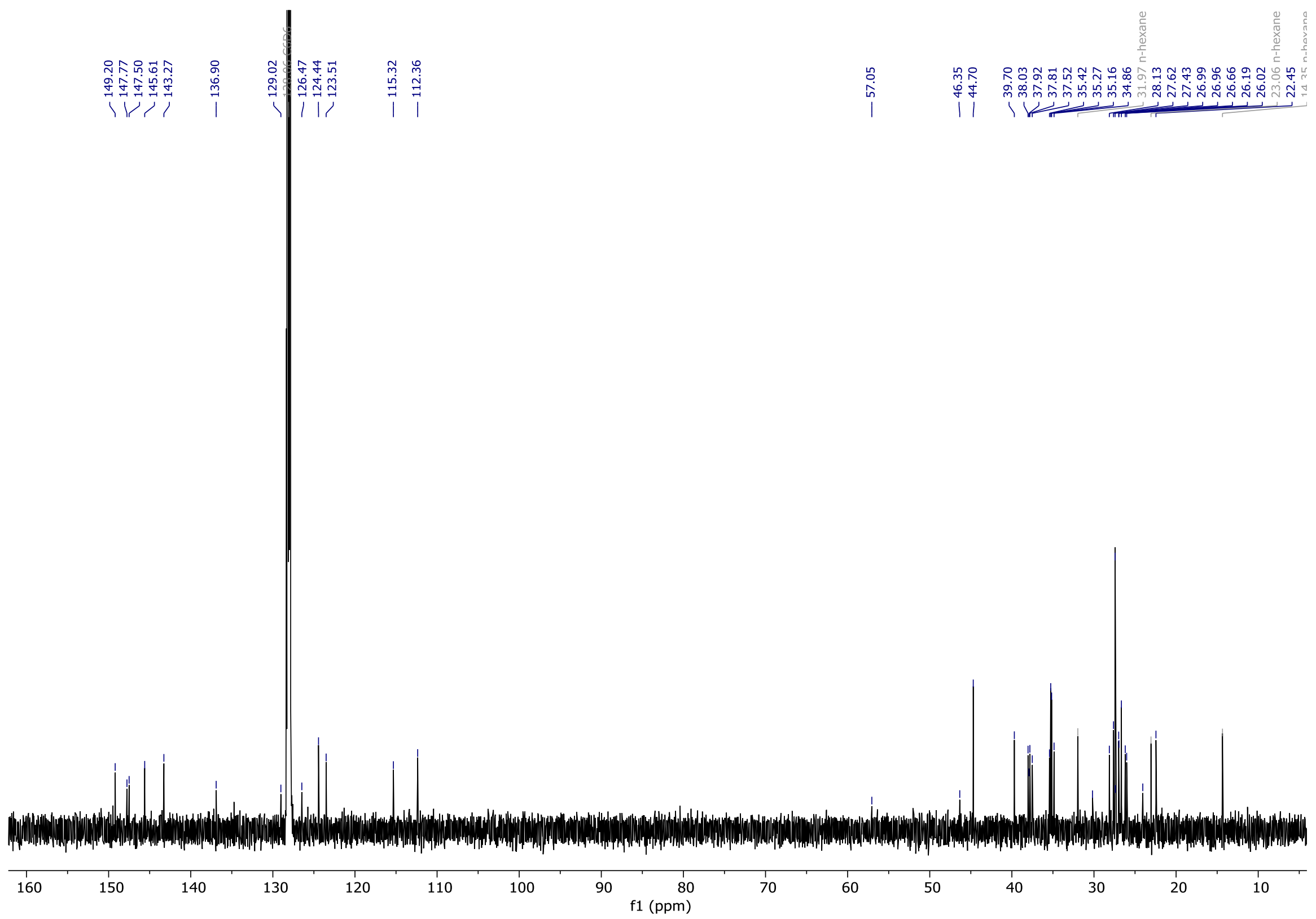

Figure S18: $^{13}C\{^{1}H\}$ NMR spectrum (101 MHz, C_6D_6) of **4**.

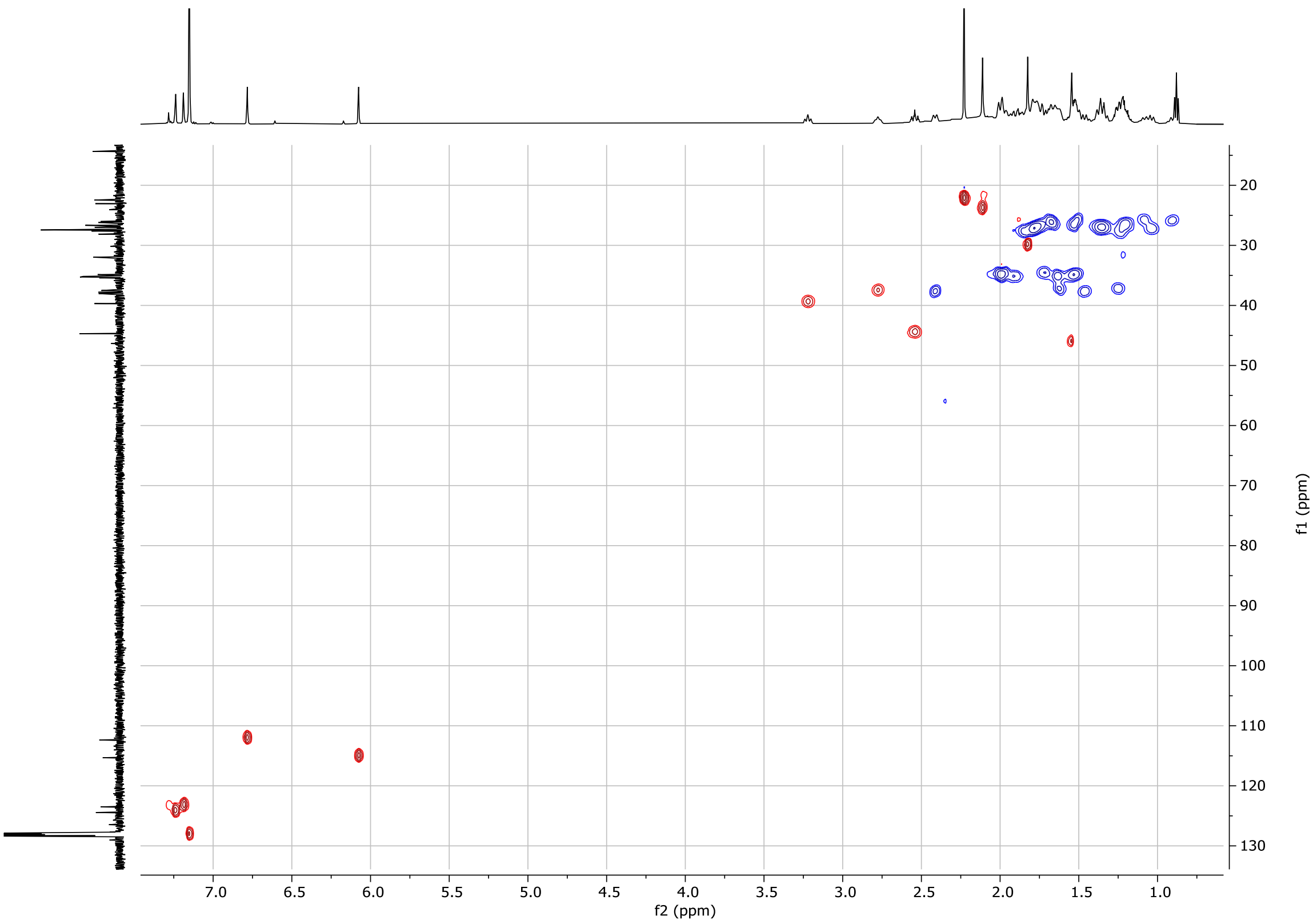

Figure S19: HSQC NMR spectrum (C_6D_6) of **4**.

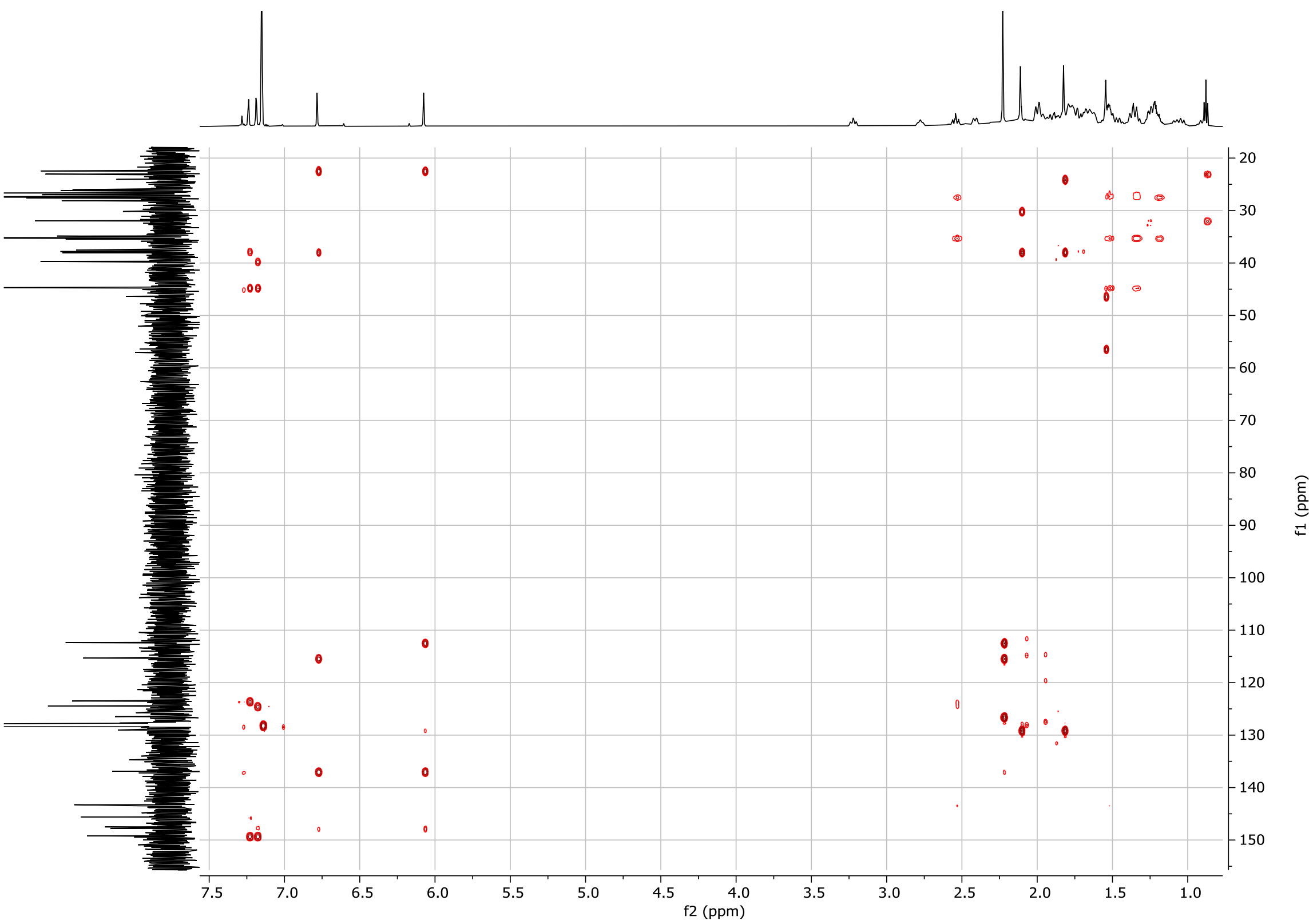

Figure S20: HMBC NMR spectrum (C_6D_6) of **4**.

Preparation of $[Na(C_6H_6)][Al(NNN^{TCHP})(H)]$ (5)

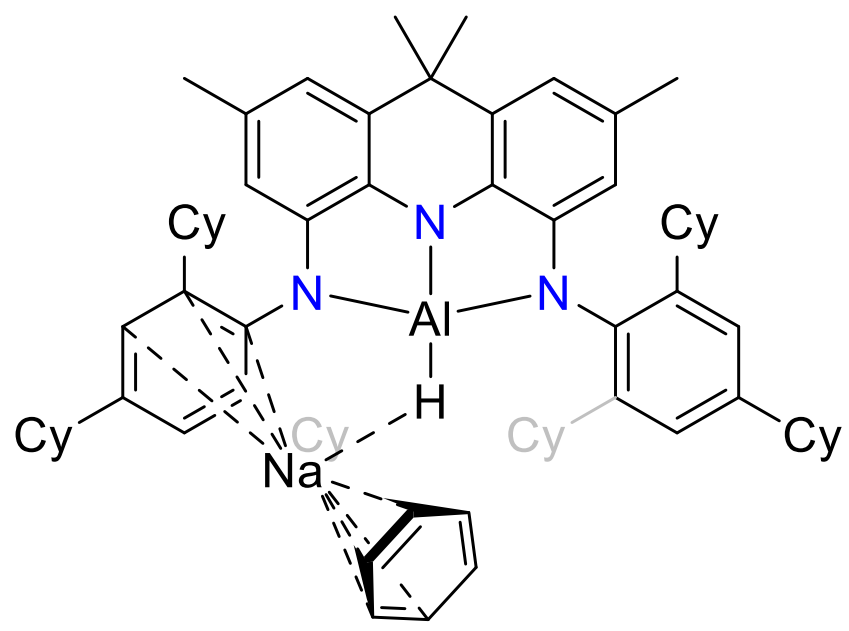

A sodium mirror (100 mg, 4.35 mmol) was prepared in a flame-dried Schlenk flask. $[Al(NNN^{TCHP})(I)_2]$ (350 mg, 0.29 mmol) was charged to the Schlenk flask and benzene was added (~10 mL). The reaction mixture was allowed to stir at room temperature for 18 hours to give an orange solution. The solution was filtered *via.* cannula and the volatiles reduced *in vacuo* (to ~1 mL). Colourless crystals form upon storage of the mother liquor at room temperature for 18 hours. **Yield**: 93 mg, 31 %.

m.p.: 238 – 240 °C (dec).

^{1}H NMR (400 MHz, C_6D_6:THF-D_8, 1:10): δ 1.18 – 1.39 (m, 14H, Cy–$\underline{H}$), 1.45 – 1.71 (m, 22H, Cy–$\underline{H}$), 1.73 – 1.88 (m, 16H, Cy–$\underline{H}$), 1.89 – 1.96 (m, 4H , Cy–$\underline{H}$), 2.00 (s, 6H, C(C$\underline{H}_3$)$_2$), 2.14 – 2.20 (m, 4H, Cy–$\underline{H}$), 2.26 (s, 6H, C$\underline{H}_3$), 2.47 – 2.55 (m, 2H, Cy–$\underline{H}$), 2.94 – 3.12 (m, 4H, Cy–$\underline{H}$), 6.05 (s, 2H, $_{ACR}$Ar–$\underline{H}$), 6.74 (s, 2H, $_{ACR}$Ar–$\underline{H}$), 7.13 (s, 4H, $_{TCHP}$Ar–$\underline{H}$), 7.17 (s, 6H, $C_6\underline{H}_6$).

^{13}C{^{1}H} NMR (101 MHz, C_6D_6:THF-D_8, 1:10): δ 22.7 ($\underline{C}$H$_3$), 26.7, 26.8, 27.2, 27.4, 27.5 (Cy–$\underline{C}$), 34.2 (C($\underline{C}$H$_3$)$_2$), 35.4 (Cy–$\underline{C}$), 38.0 ($\underline{C}$(CH$_3$)$_2$), 38.2, 45.0 (Cy–$\underline{C}$), 112.3, 113.8, 123.2, 124.9, 125.2, 128.6, 130.0, 143.0, 145.6, 146.6, 146.7 ($_{TCHP}$Ar–$\underline{C}$, $_{ACR}$Ar–$\underline{C}$, $\underline{C}_6$H$_6$).

Note: The title compound is poorly soluble in aliphatic and arene solvents. Satisfactory NMR spectroscopic data could only be obtained in the presence of THF–D_8. However, we suspect that ethereal donors such as THF react with the title compound to give "$[Al(NNN^{TCHP})(L)_n]$" (where L = THF in this case). Interpretation of the NMR spectra of **5** in the presence of THF-D_8 should be treated with caution.

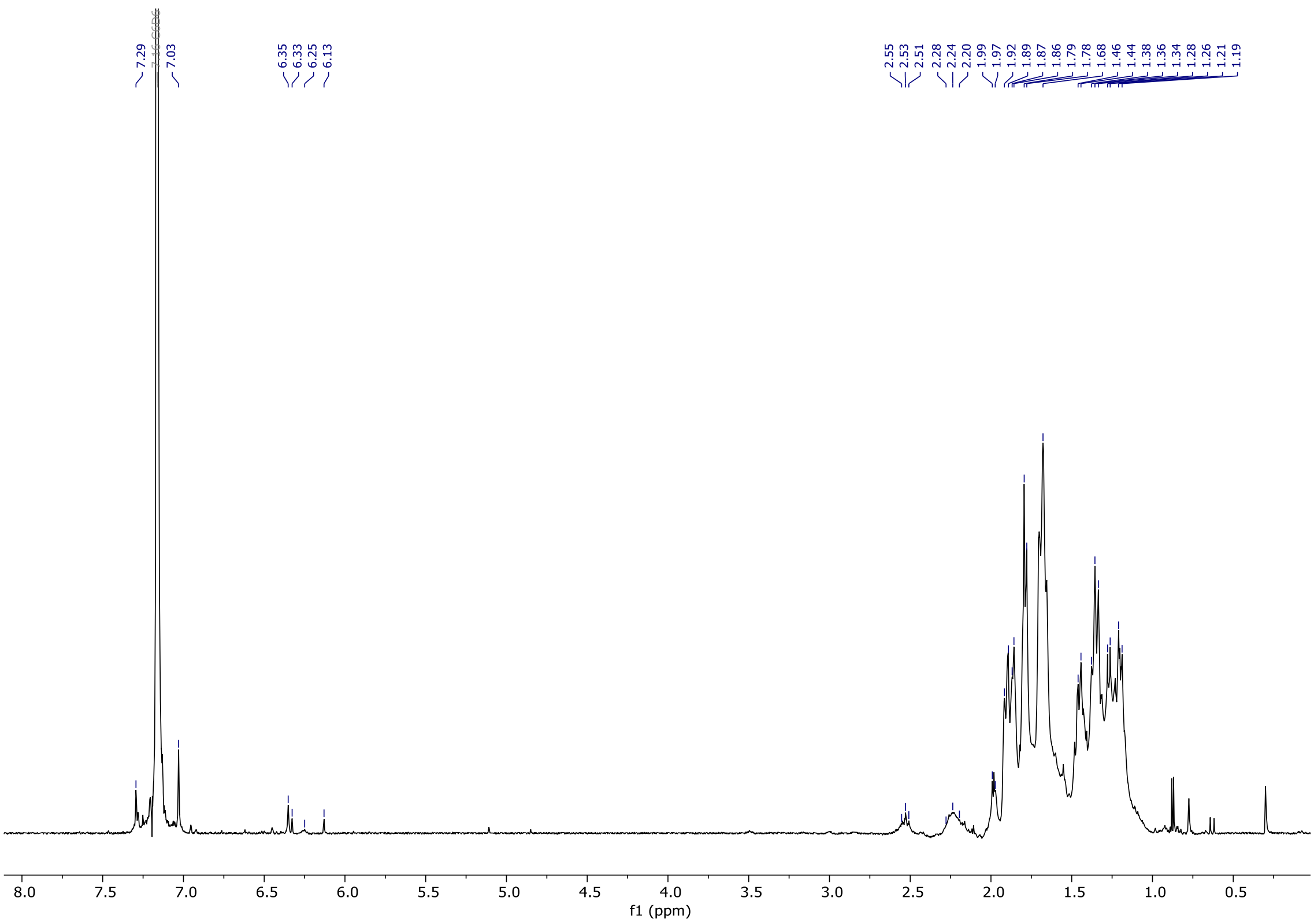

Figure S21: ^{1}H NMR spectrum (400 MHz, C_6D_6) of **5** (poorly soluble).

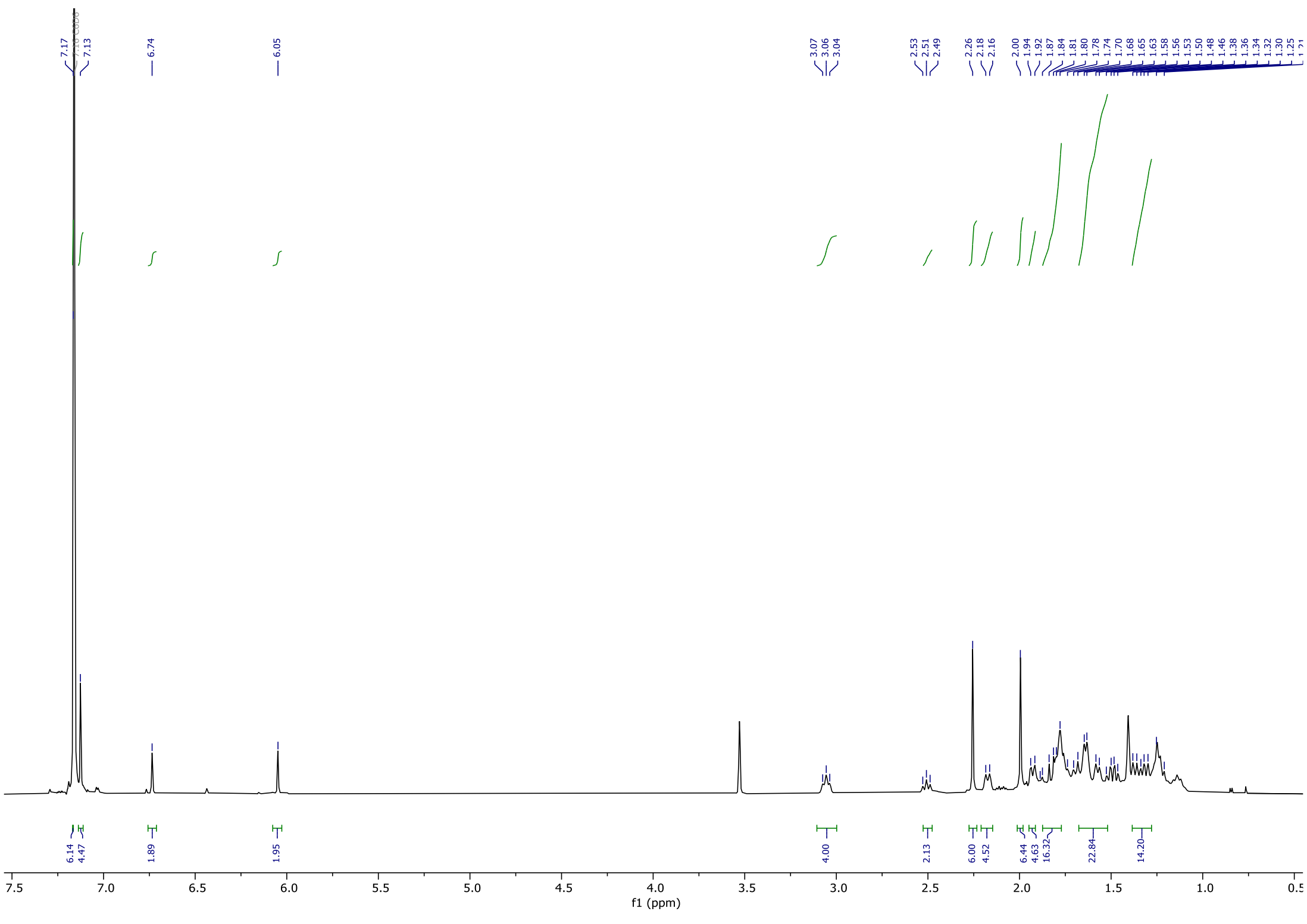

Figure S22: ^{1}H NMR spectrum (400 MHz, C_6D_6:THF-D_8, 1:10) of **5**.

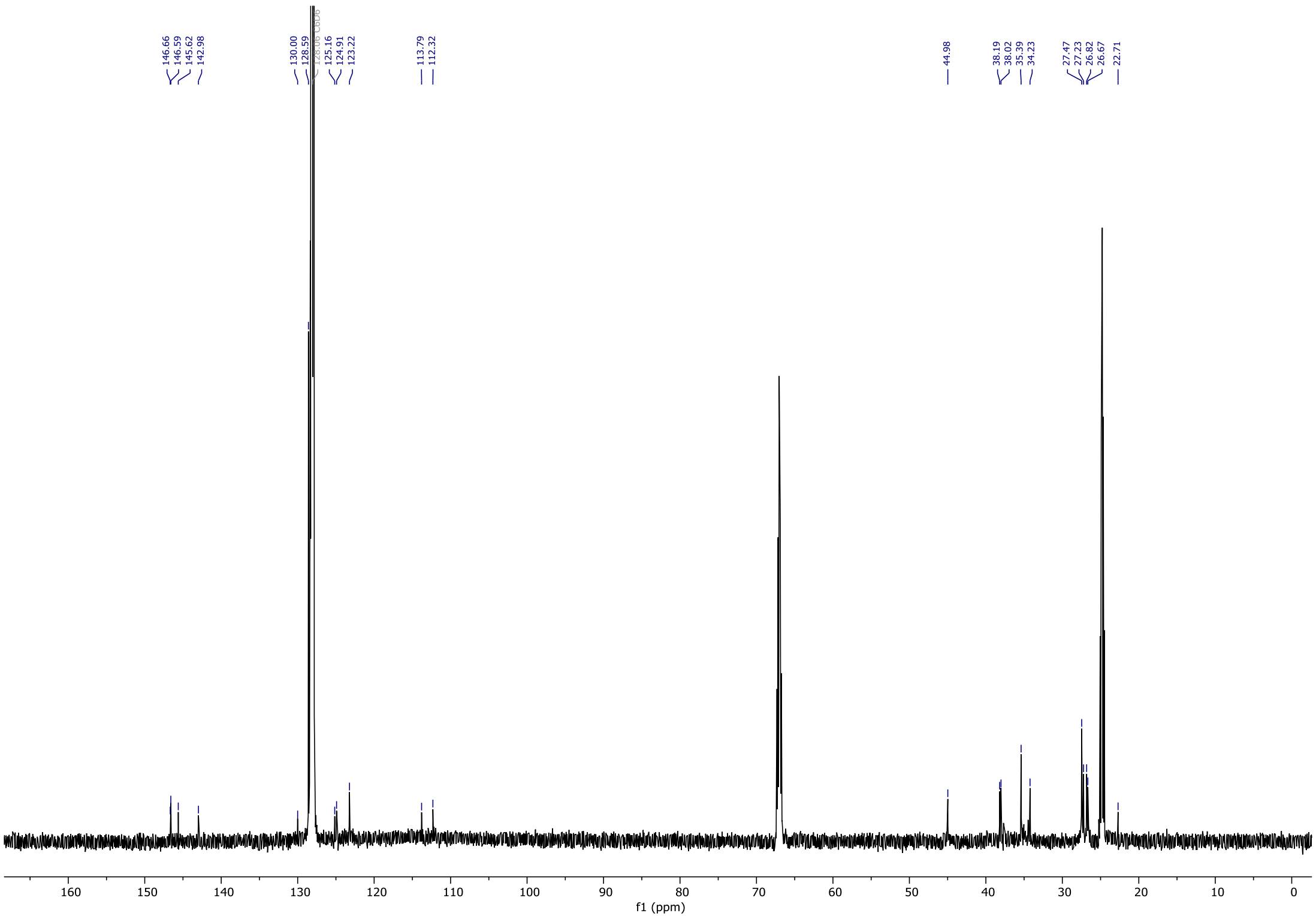

Figure S23: $^{13}C\{^{1}H\}$ NMR spectrum (101 MHz, C_6D_6:THF-D_8, 1:10) of **5**.

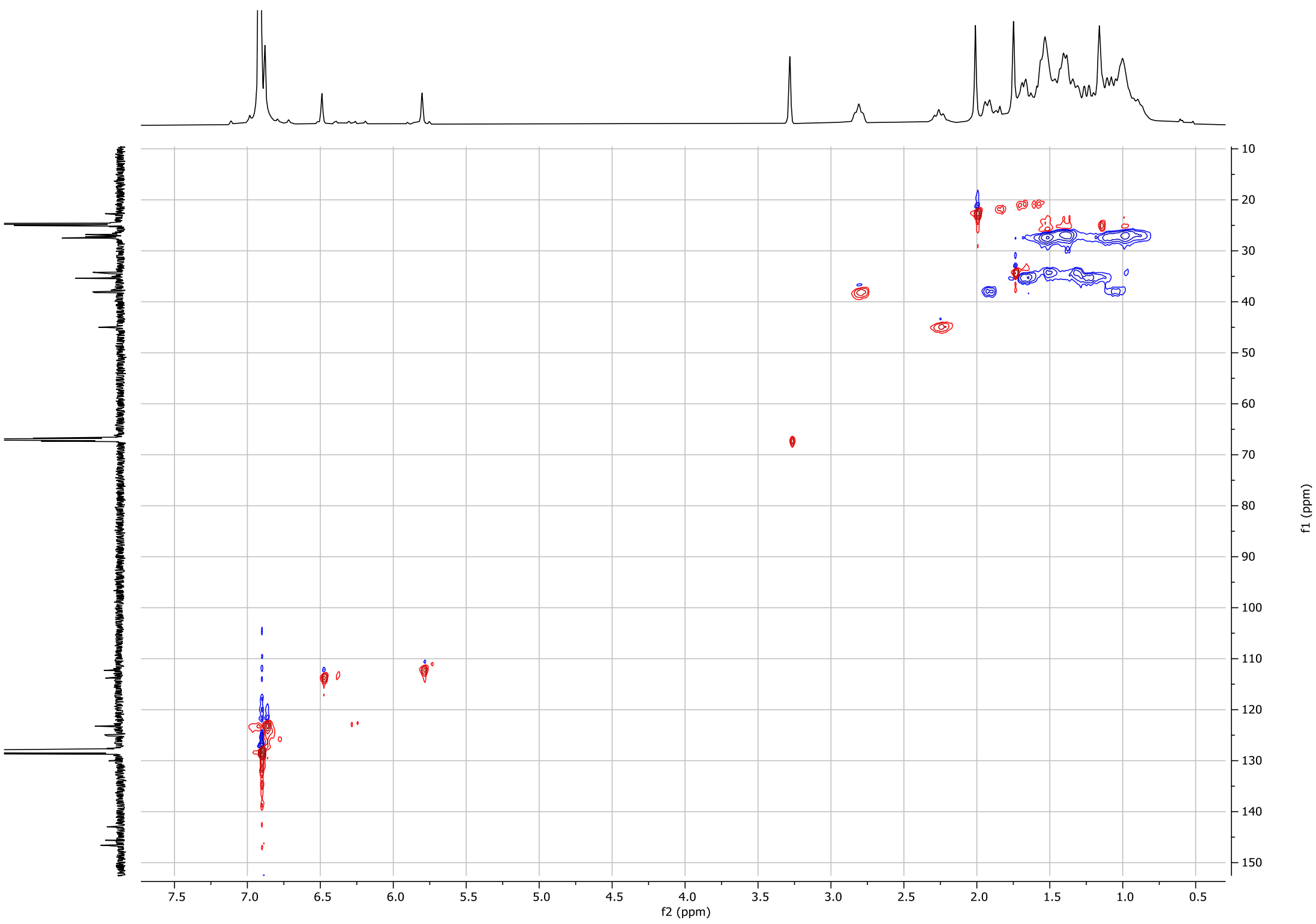

Figure S24: HSQC NMR spectrum (C_6D_6:THF-D_8, 1:10) of **5**.

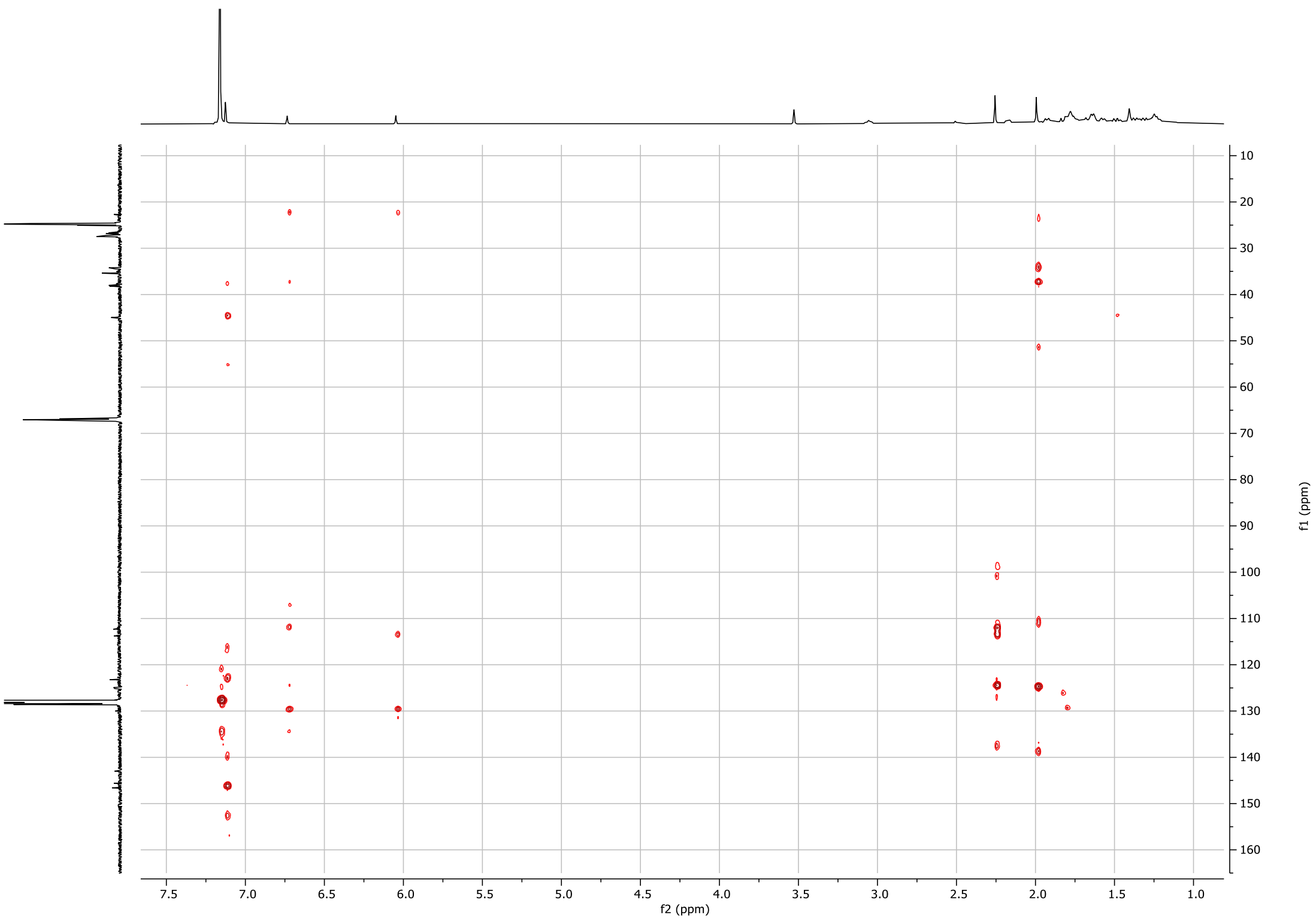

Figure S25: HMBC NMR spectrum (C_6D_6:THF-D_8, 1:10) of **5**.

Preparation of [Al(NNN^{TCHP})(2.2.2-cryptand)] (6)

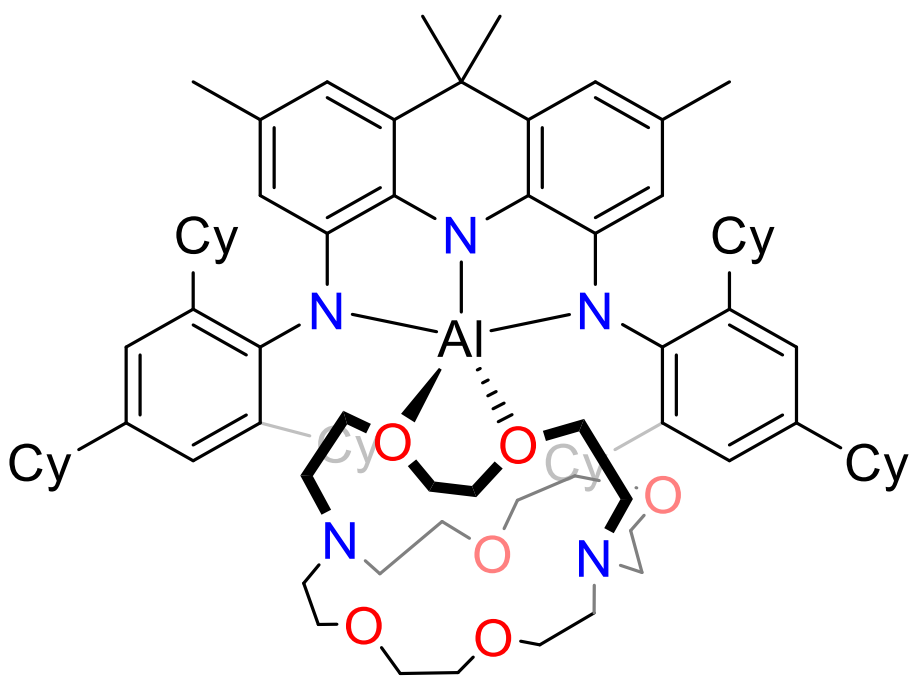

[Na(C_6H_6)][Al(NNN^{TCHP})(H)] (15 mg, 0.14 mmol) and 2.2.2-cryptand (6 mg, 0.14 mmol) were charged to J Young fitted NMR tube and benzene-D_6 (~0.6 mL) was added. The resulting red solution (containing a precipitate) was filtered through celite and concentrated to *ca.* 0.1 mL and stored at room temperature for 72 hours to give a small number of colourless crystals suitable for single crystal X-ray diffraction measurements.

Note: NMR spectra of the title complex, as part of the reaction mixture, were collected *in situ* due to the low isolated yield. Assignments were made tentatively by analysis of the 2D NMR spectra.

^{1}H NMR (400 MHz, C_6D_6): δ 1.32 – 1.49 (m, 14H, Cy–*H*), 1.52 – 1.84 (m, 30H, Cy–*H*), 1.85 – 1.96 (m, 6H, Cy–*H*), 1.96 – 2.06 (m, 18H, crypt–CH_2, C(CH_3)$_2$), 2.07 – 2.18 (m, 6H, Cy–*H*), 2.21 (s, 6H, CH_3), 2.57 – 2.69 (m, 6H, Cy–*H*), 2.88 – 3.00 (m, 24H, crypt–CH_2), 3.31 – 3.44 (m, 4H, Cy–*H*), 6.23 (s, 2H, $_{ACR}$Ar–*H*), 6.68 (s, 2H, $_{ACR}$Ar–*H*), 7.35 (s, 4H, $_{TCHP}$Ar–*H*).

^{13}C{^{1}H} NMR (101 MHz, C_6D_6): δ 22.7 (*C*H$_3$), 26.5, 26.9, 27.3, 27.5 (Cy–*C*), 27.7 (C(*C*H$_3$)$_2$), 28.0, 28.1, 35.0, 36.4 (Cy–*C*), 38.2 (*C*(CH$_3$)$_2$), 39.7, 45.2 (Cy–*C*), 53.0, 67.6, 68.4 (crypt–*C*H$_2$), 111.3, 112.6, 122.9, 126.1, 129.7, 132.7, 137.7, 138.1, 142.5, 144.7, 145.3, 145.7 ($_{TCHP}$Ar–*C*, $_{ACR}$Ar–*C*).

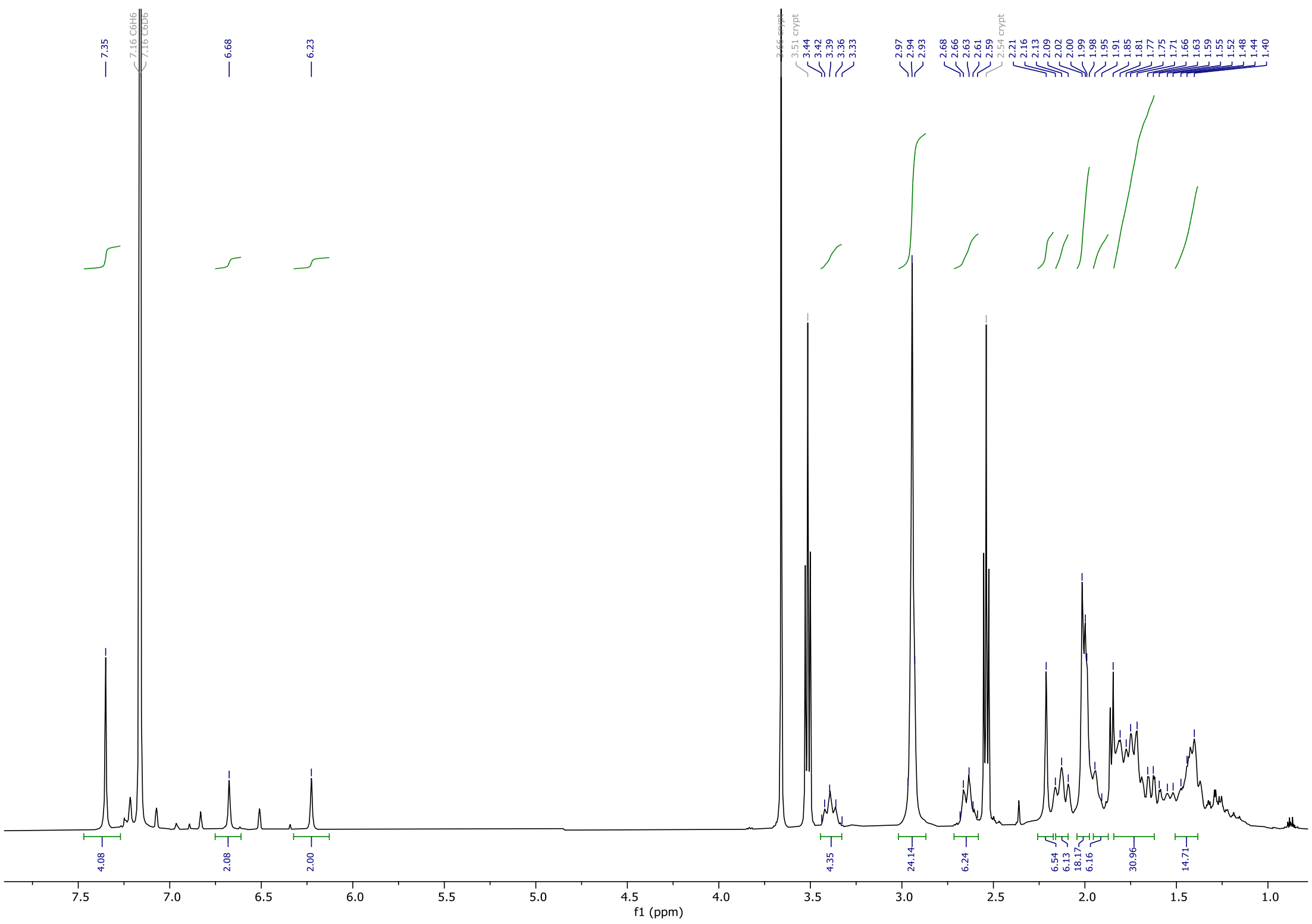

Figure S26: ^{1}H NMR spectrum (400 MHz, C_6D_6) of **6**.

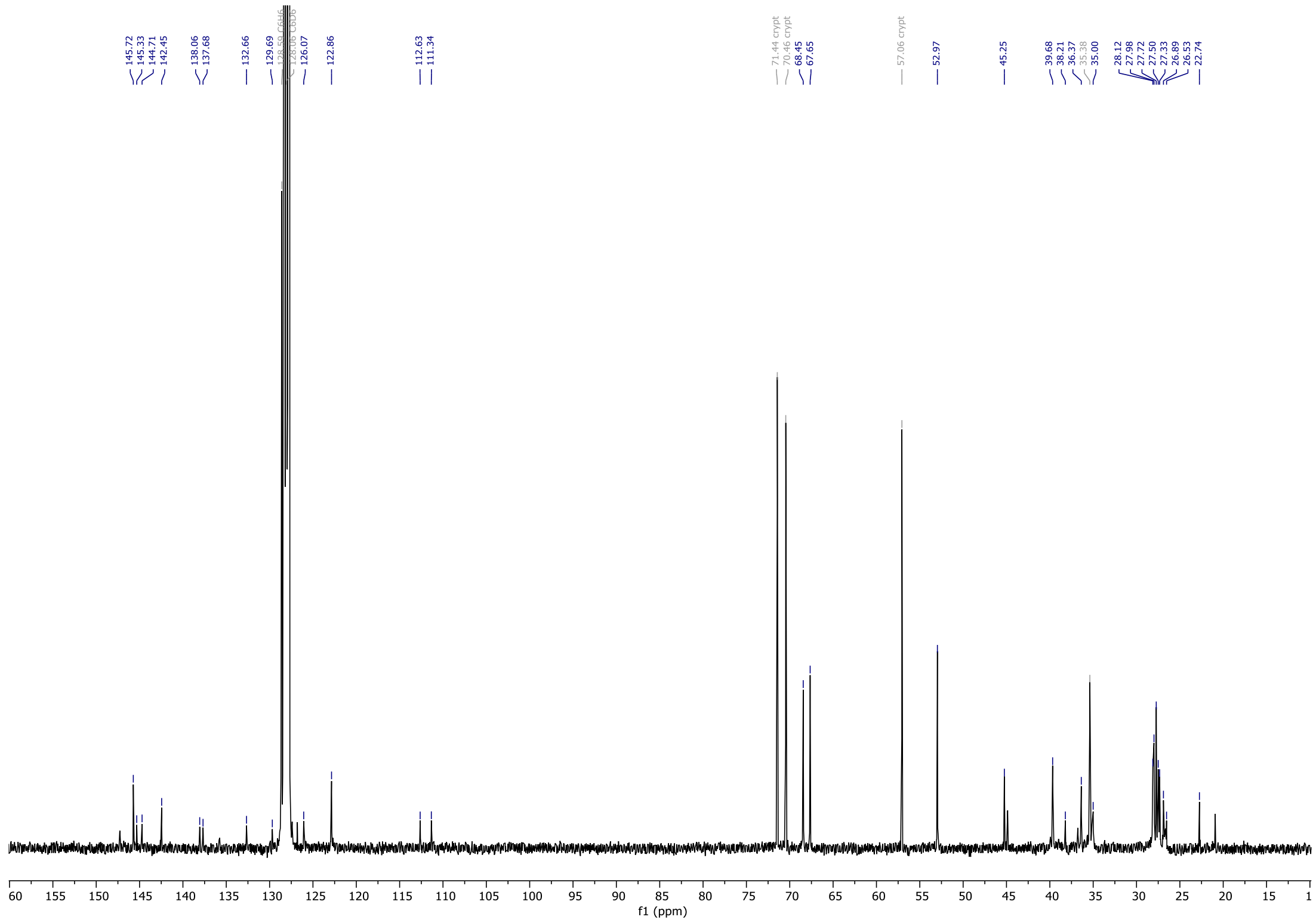

Figure S27: $^{13}C\{^{1}H\}$ NMR spectrum (101 MHz, C_6D_6) of **6**.

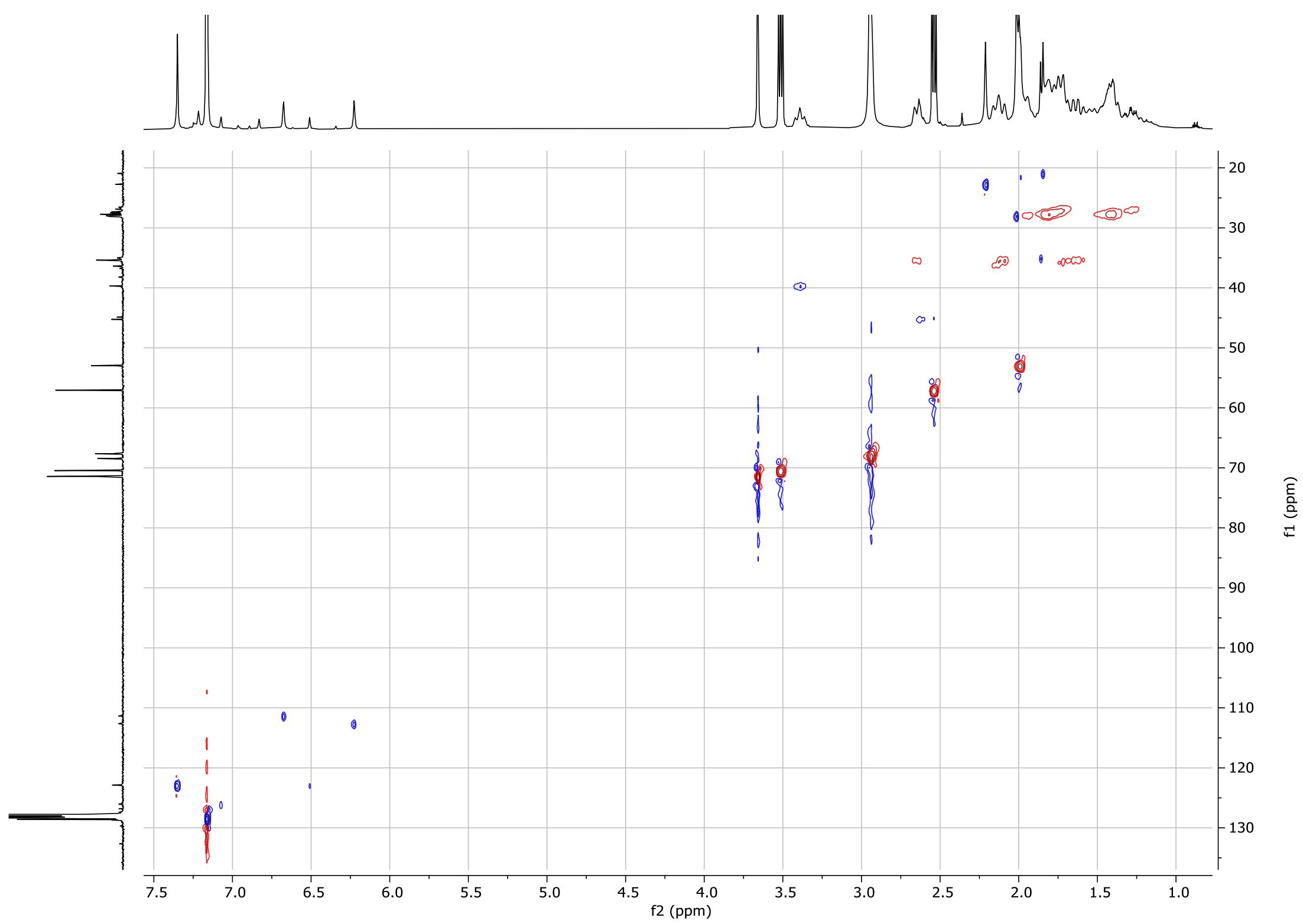

Figure S28: HSQC NMR spectrum (C_6D_6) of **6**.

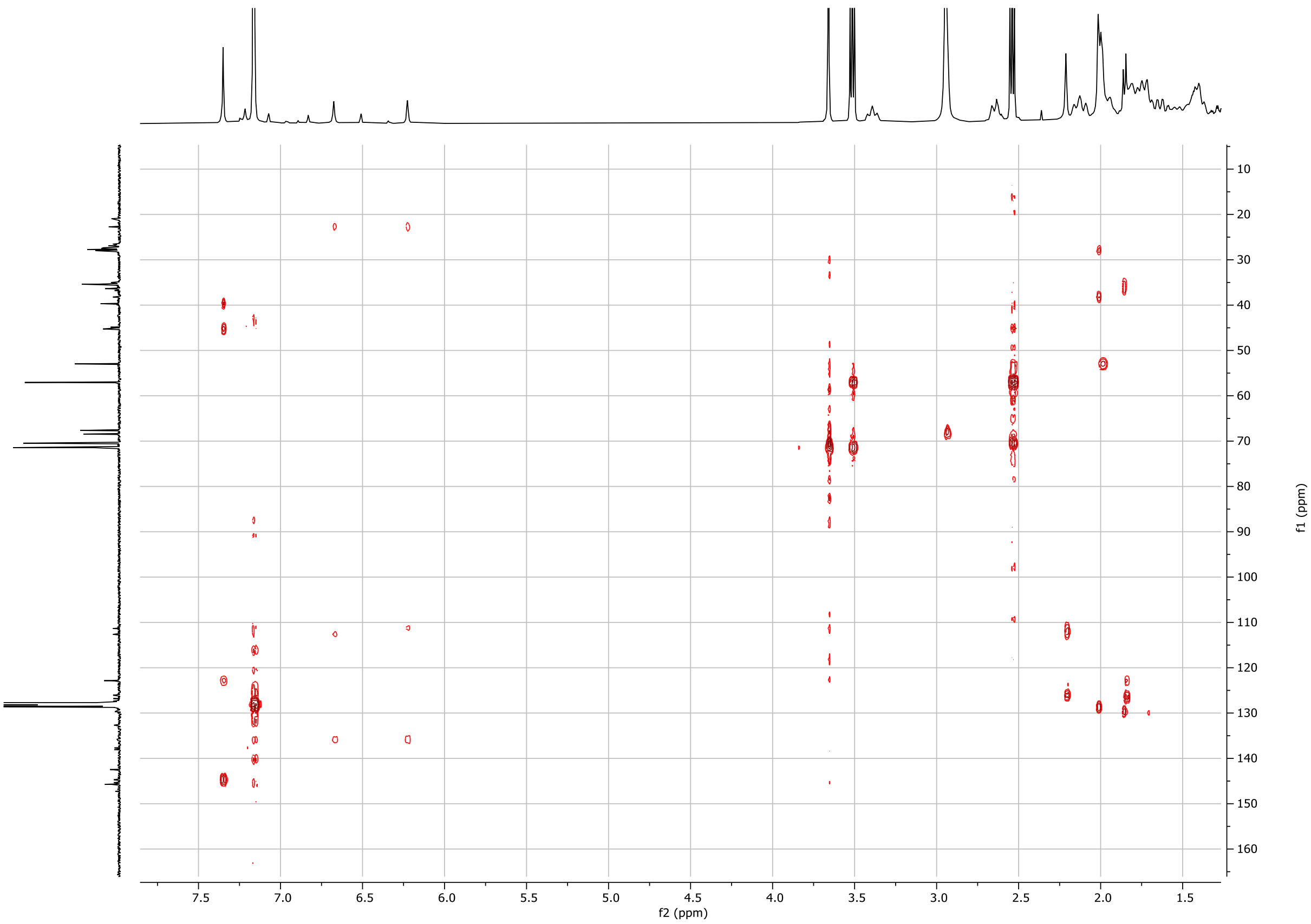

Figure S29: HMBC NMR spectrum (C_6D_6) of **6**.

2. Crystallographic Details

X-ray Crystallographic Details

Crystals suitable for X-ray structural determination were mounted in silicone oil. Crystallographic measurements were made using a Rigaku Xtalab Synergy Dualflex using a graphite monochromator with Cu Kα (1.54180 Å) or Mo Kα (0.71073 Å), or the MX1/MX2 beamlines of the Australian Synchrotron (λ = 0.71090 Å).[S4],[S5] The software package Blu-Ice[S6] was used for synchrotron data acquisition, while the program XDS[S7] was employed for synchrotron data reduction. All structures were solved by direct methods and refined on F^2 by full matrix least squares (SHELX-18)[S8] using all unique data. Hydrogen atoms were included in calculated positions (riding model). Crystal data, details of data collections, and refinements for all structures can be found in their CIF files and are summarized in Table S1.

Table S1: Crystallographic Data for Isolated Compounds

Compound	$(NNN^{TCHP})H_3$		$(NNN^{DCHP})H_3$		**1**	
Empirical formula	$C_{77}H_{101}N_3$		$C_{53}H_{69}N_3$		$C_{74}H_{105}Al_2N_3$	
Formula weight	1068.6		748.11		1090.56	
Temperature	100(2) K		122.98(14) K		123.01(10) K	
Crystal system	Monoclinic		Monoclinic		Monoclinic	
Space group	C2/*m*		C2/*c*		$P2_1/n$	
Unit cell dimensions	a = 23.859(5) Å	α = 90°	a = 22.6362(6) Å	α = 90°	a = 11.00990(10) Å	α = 90°
	b = 24.448(5) Å	β = 101.72(3)°	b = 10.0549(3) Å	β = 102.509(3)°	b = 31.5023(2) Å	β = 91.4170(10)°
	c = 11.234(2) Å	γ = 90°	c = 19.3610(5) Å	γ = 90°	c = 21.27710(10) Å	γ = 90°
Volume	6416(2) Å^3		4302.0(2) Å^3		7377.43(9) Å^3	
Z	4		4		4	
Density (calculated)	1.106 Mg/m^3		1.155 Mg/m^3		0.982 Mg/m^3	
Absorption coefficient	0.063 mm^{-1}		0.495 mm^{-1}		0.633 mm^{-1}	
F(000)	2336		1632		2384	
Crystal size	0.120 x 0.080 x 0.040 mm^3		0.190 x 0.090 x 0.070 mm^3		0.490 x 0.110 x 0.100 mm^3	
Theta range for data collection	1.743 to 26.374°		4.001 to 80.287°		3.492 to 80.342°	
Index ranges	-29<=*h*<=29, -29<=*k*<=30, -13<=*l*<=13		-28<=*h*<=28, -12<=*k*<=9, -23<=*l*<=24		-14<=*h*<=13, -40<=*k*<=33, -25<=*l*<=27	
Reflections collected	56501		23142		78699	
Independent reflections	6567 [R(int) = 0.0838]		4605 [R(int) = 0.0590]		15759 [R(int) = 0.0423]	
Completeness to theta = 67.684 °	99.40%		100.00%		99.80%	
Absorption correction	Semi-empirical from equivalents		Gaussian		Gaussian	
Max. and min. transmission	Value not reported by XDS		1.000 and 0.733		1.000 and 0.402	
Data / restraints / parameters	6567 / 274 / 457		4605 / 0 / 262		15759 / 546 / 891	
Goodness-of-fit on F^2	1.049		1.065		1.056	
Final R indices [I > 2sigma(I)]	R_1 = 0.0554, wR_2 = 0.1350		R_1 = 0.0620, wR_2 = 0.1714		R_1 = 0.0611, wR_2 = 0.1793	
R indices (all data)	R_1 = 0.0740, wR_2 = 0.1488		R_1 = 0.0752, wR_2 = 0.1852		R_1 = 0.0666, wR_2 = 0.1855	
Largest diff. peak and hole	0.163 and -0.276 e.Å^{-3}		0.724 and -0.321 e.Å^{-3}		0.867 and -0.351 e.Å^{-3}	
CCDC Number	2549939		2549934		2549941	

Table S1 (contd.): Crystallographic Data for Isolated Compounds

Compound	2		3		4	
Empirical formula	$C_{65} H_{86} Al I_2 N_3$		$C_{107} H_{128} Al I K N_3$		$C_{83} H_{114} Al N_5$	
Formula weight	1190.14		1649.1		1208.77	
Temperature	100(2) K		123.00(10) K		100(2) K	
Crystal system	Monoclinic		Monoclinic		Triclinic	
Space group	P2$_1$/*c*		P2$_1$/*c*		P-1	
Unit cell dimensions	a = 24.400(5) Å	α = 90°	a = 18.3712(4) Å	α = 90°	a = 11.440(2) Å	α = 85.25(3)°
	b = 11.740(2) Å	β = 110.39(3)°	b = 23.9899(3) Å	β = 95.112(2)°	b = 17.000(3) Å	β = 77.22(3)°
	c = 21.910(4) Å	γ = 90°	c = 20.7124(3) Å	γ = 90°	c = 18.830(4) Å	γ = 88.26(3)°
Volume	5883(2) Å^3		9092.1(3) Å^3		3558.8(13) Å^3	
Z	4		4		2	
Density (calculated)	1.344 Mg/m^3		1.205 Mg/m^3		1.128 Mg/m^3	
Absorption coefficient	1.125 mm^{-1}		3.631 mm^{-1}		0.076 mm^{-1}	
F(000)	2464		3504		1320	
Crystal size	0.120 x 0.060 x 0.020 mm3		0.210 x 0.100 x 0.090 mm3		0.080 x 0.040 x 0.010 mm^3	
Theta range for data collection	0.890 to 26.373°		3.591 to 80.445°		1.112 to 26.373°	
Index ranges	-30<=*h*<=30, -14<=*k*<=14, -27<=*l*<=27		-22<=*h*<=23, -30<=*k*<=15, -26<=*l*<=25		-14<=*h*<=14, -21<=*k*<=21, -23<=*l*<=23	
Reflections collected	140835		78504		47543	
Independent reflections	11987 [R(int) = 0.1836]		19401 [R(int) = 0.0629]		14068 [R(int) = 0.0635]	
Completeness to theta = 67.684 °	99.90%		99.70%		97.30%	
Absorption correction	Semi-empirical from equivalents		Gaussian		Semi-empirical from equivalents	
Max. and min. transmission	Value not reported by XDS		1.000 and 0.463		Value not reported by XDS	
Data / restraints / parameters	11987 / 0 / 644		19401 / 700 / 1132		14068 / 0 / 702	
Goodness-of-fit on F^2	1.017		1.071		0.933	
Final R indices [I > 2sigma(I)]	R_1 = 0.0466, wR_2 = 0.1204		R_1 = 0.0670, wR_2 = 0.1657		R_1 = 0.0621, wR_2 = 0.1613	
R indices (all data)	R_1 = 0.0560, wR_2 = 0.1270		R_1 = 0.0880, wR_2 = 0.1795		R_1 = 0.0912, wR_2 = 0.1853	
Largest diff. peak and hole	1.587 and -0.874 e.Å^{-3}		2.151 and -0.792 e.Å^{-3}		0.350 and -0.396 e.Å^{-3}	
CCDC Number	2549936		2549937		2549940	

Table S1 (contd.): Crystallographic Data for Isolated Compounds

Compound	**5**		**6**	
Empirical formula	$C_{107} H_{129} Al N_3 Na$		$C_{95} H_{134} Al N_5 O_6$	
Formula weight	1507.09		1469.04	
Temperature	100(2) K		123(2) K	
Crystal system	Monoclinic		Triclinic	
Space group	P2$_1$/*m*		P-1	
Unit cell dimensions	a = 10.360(2) Å	α = 90°	a = 12.8122(3) Å	α = 80.6330(10)°
	b = 23.800(5) Å	β = 95.64(3)°	b = 13.4065(2) Å	β = 76.412(2)°
	c = 18.180(4) Å	γ = 90°	c = 25.1764(5) Å	γ = 87.9960(10)°
Volume	4460.9(16) $Å^3$		4147.36(15) $Å^3$	
Z	2		2	
Density (calculated)	1.122 Mg/m^3		1.176 Mg/m^3	
Absorption coefficient	0.077 mm^{-1}		0.653 mm^{-1}	
F(000)	1632		1600	
Crystal size	0.120 x 0.030 x 0.020 mm^3		0.152 x 0.130 x 0.056 mm^3	
Theta range for data collection	1.125 to 26.369°		3.545 to 79.863°	
Index ranges	-12<=*h*<=12, -29<=*k*<=29, -22<=*l*<=22		-16<=*h*<=16, -12<=*k*<=17, -32<=*l*<=32	
Reflections collected	82228		60262	
Independent reflections	9199 [R(int) = 0.0791]		17465 [R(int) = 0.0561]	
Completeness to theta = 67.684 °	99.90%		99.90%	
Absorption correction	Semi-empirical from equivalents		Gaussian	
Max. and min. transmission	Value not reported by XDS		1.000 and 0.589	
Data / restraints / parameters	9199 / 1641 / 939		17465 / 36 / 968	
Goodness-of-fit on F^2	1.022		1.029	
Final R indices [I > 2sigma(I)]	R_1 = 0.0842, wR_2 = 0.2313		R_1 = 0.0626, wR_2 = 0.1529	
R indices (all data)	R_1 = 0.1527, wR_2 = 0.2853		R_1 = 0.0879, wR_2 = 0.1668	
Largest diff. peak and hole	0.945 and -0.156 e.$Å^{-3}$		0.397 and -0.337 e.$Å^{-3}$	
CCDC Number	2549938		2549935	

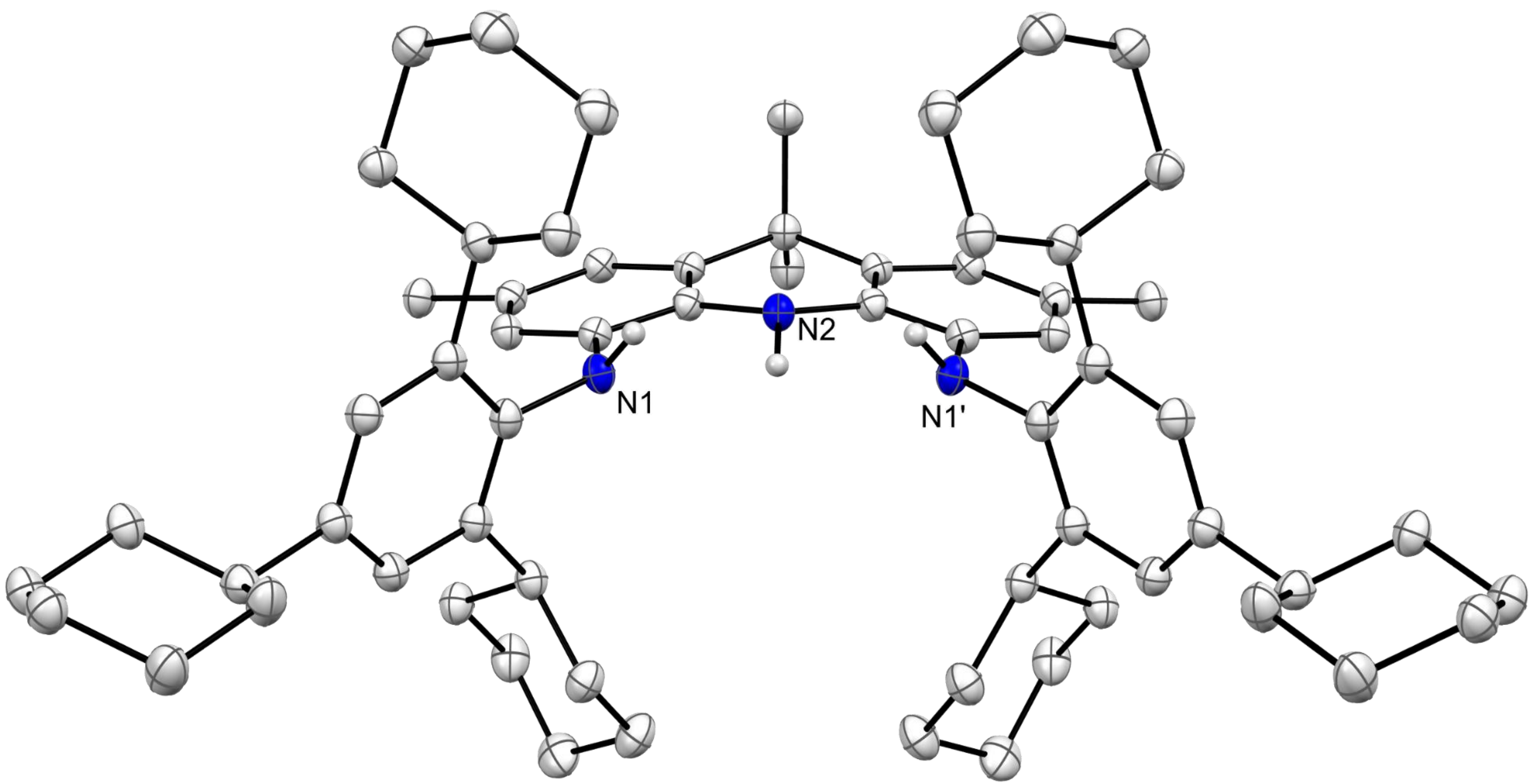

Figure S30: Molecular structure of $(NNN^{TCHP})H_3$. Thermal ellipsoids shown at 30% probability; hydrogen atoms, except amine protons, omitted; Cyclohexyl groups shown as wireframe for clarity.

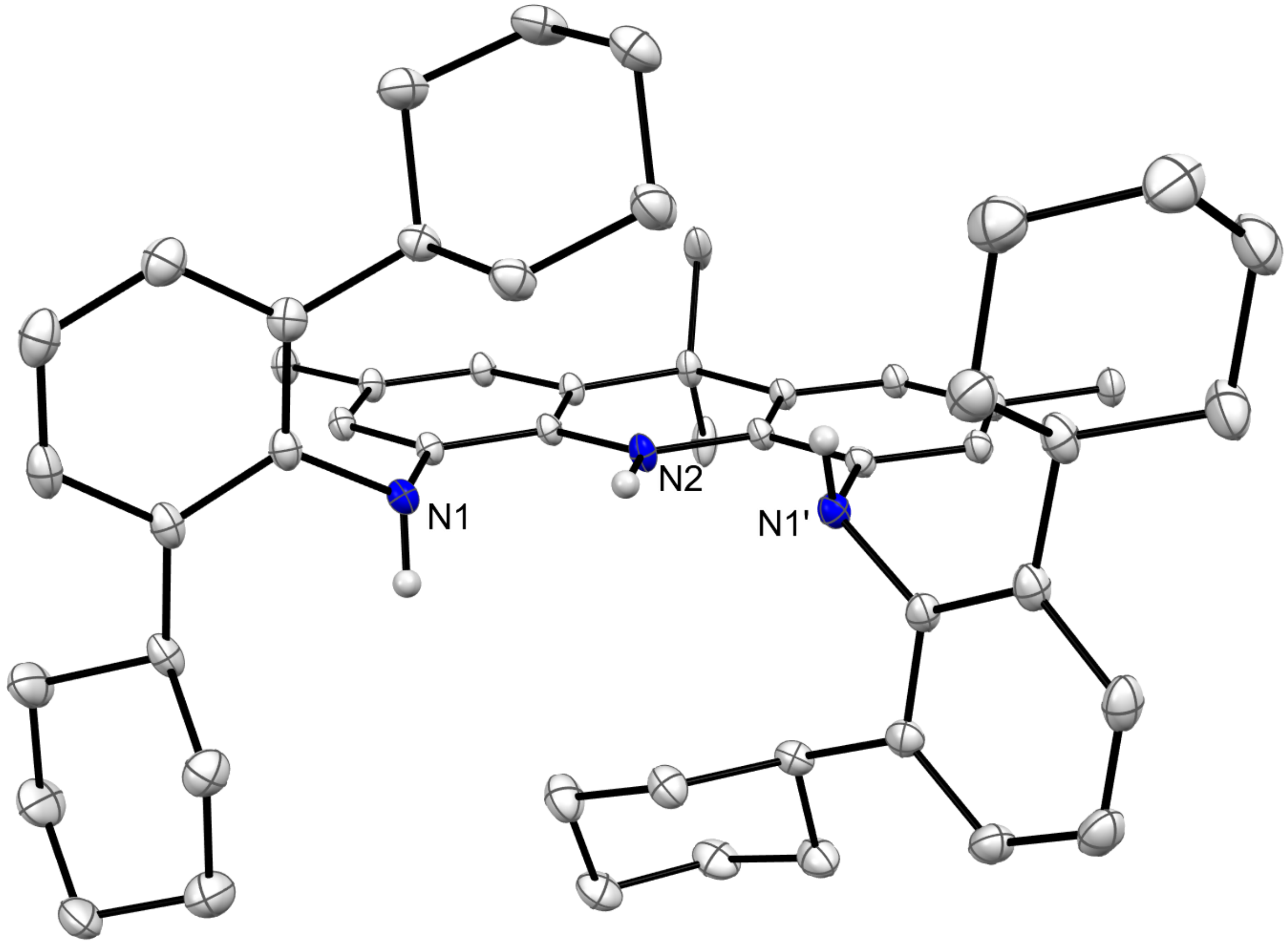

Figure S31: Molecular structure of $(NNN^{DCHP})H_3$. Thermal ellipsoids shown at 30% probability; hydrogen atoms, except amine protons, omitted; Cyclohexyl groups shown as wireframe for clarity.

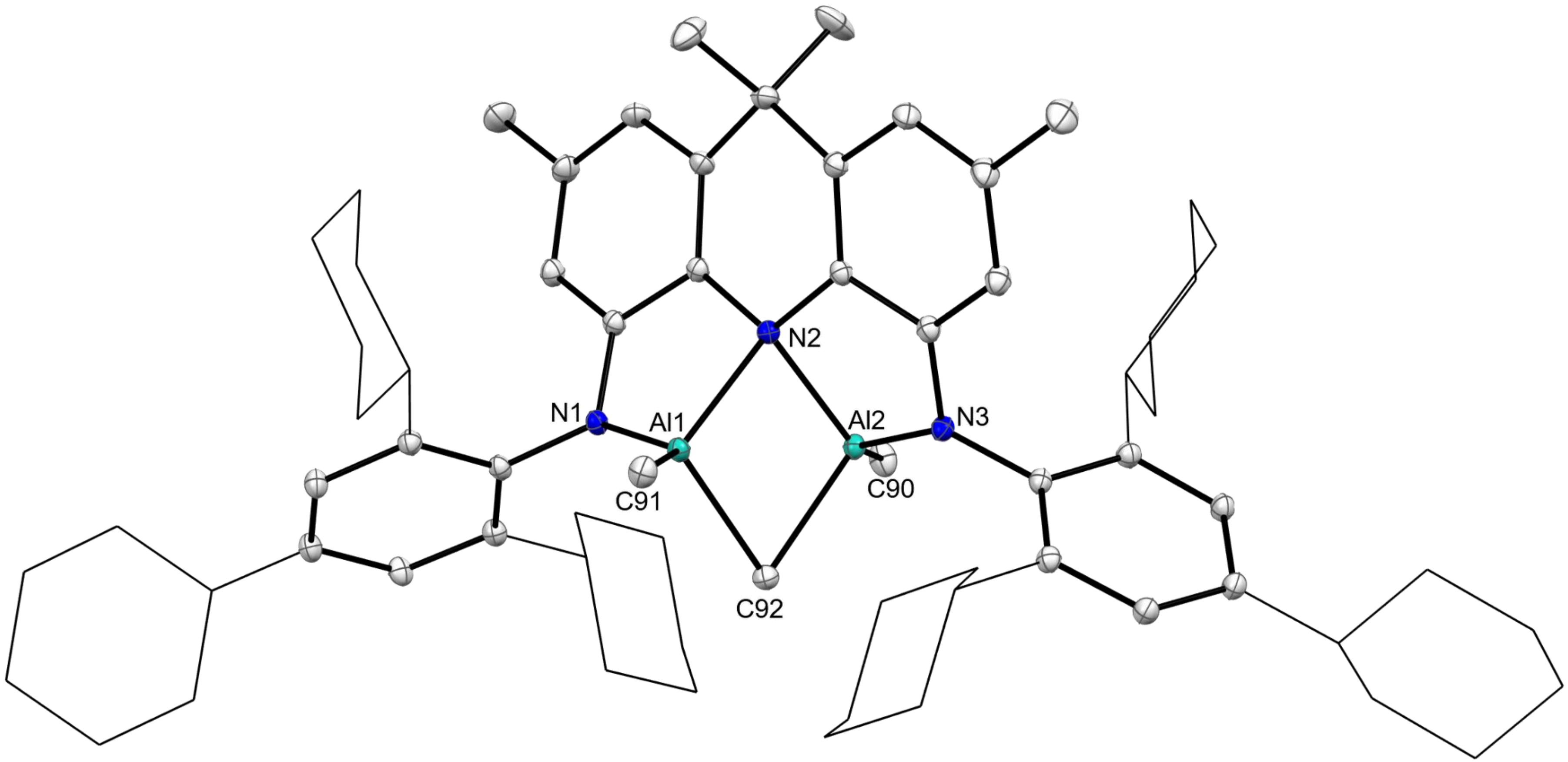

Figure S32: Molecular structure of **1**. Thermal ellipsoids shown at 30% probability; hydrogen atoms omitted; Cyclohexyl groups shown as wireframe for clarity.

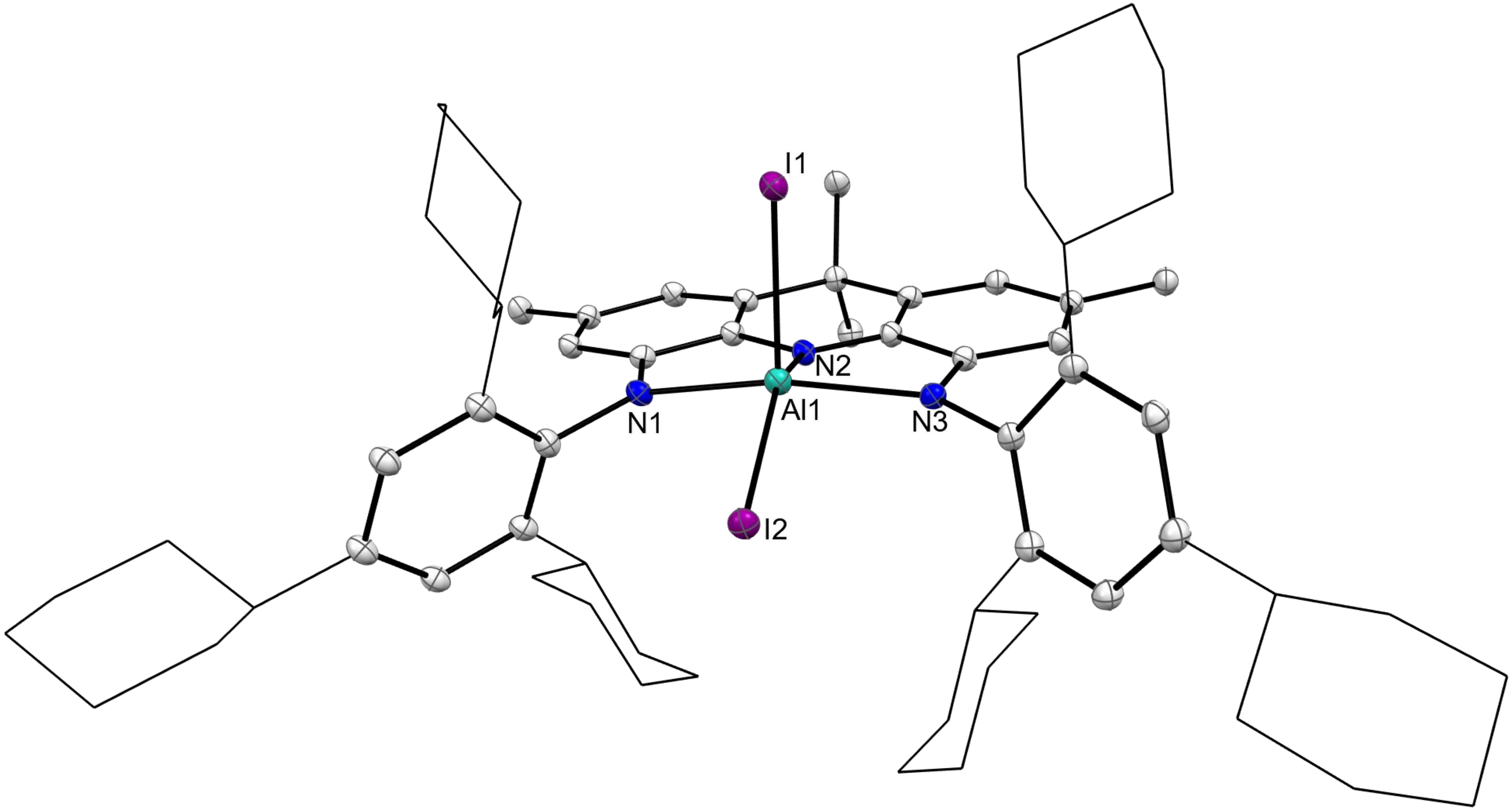

Figure S33: Molecular structure of **2**. Thermal ellipsoids shown at 30% probability; hydrogen atoms omitted; Cyclohexyl groups shown as wireframe for clarity.

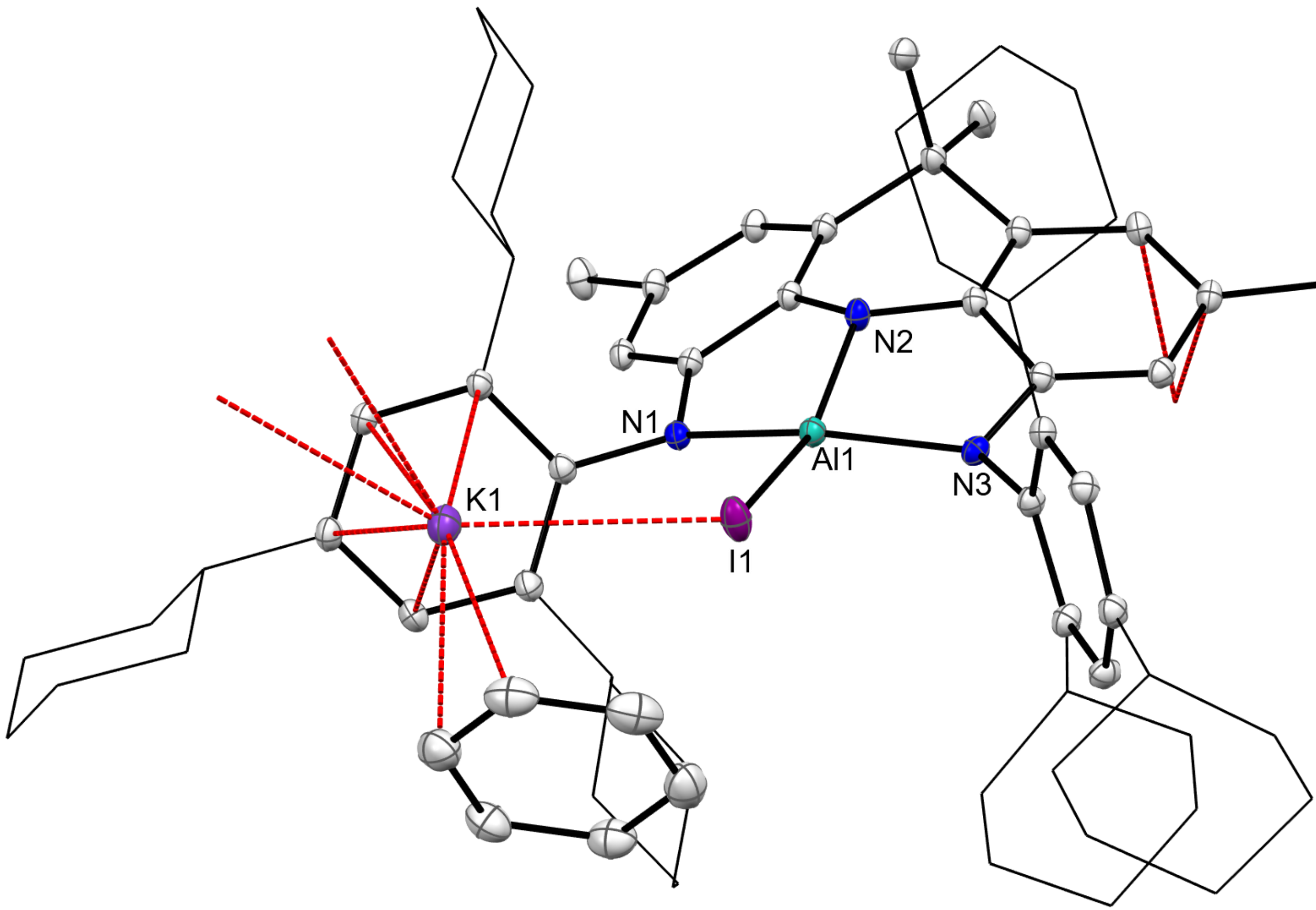

Figure S34: Molecular structure of **3**. Thermal ellipsoids shown at 30% probability; hydrogen atoms omitted; Cyclohexyl groups shown as wireframe for clarity.

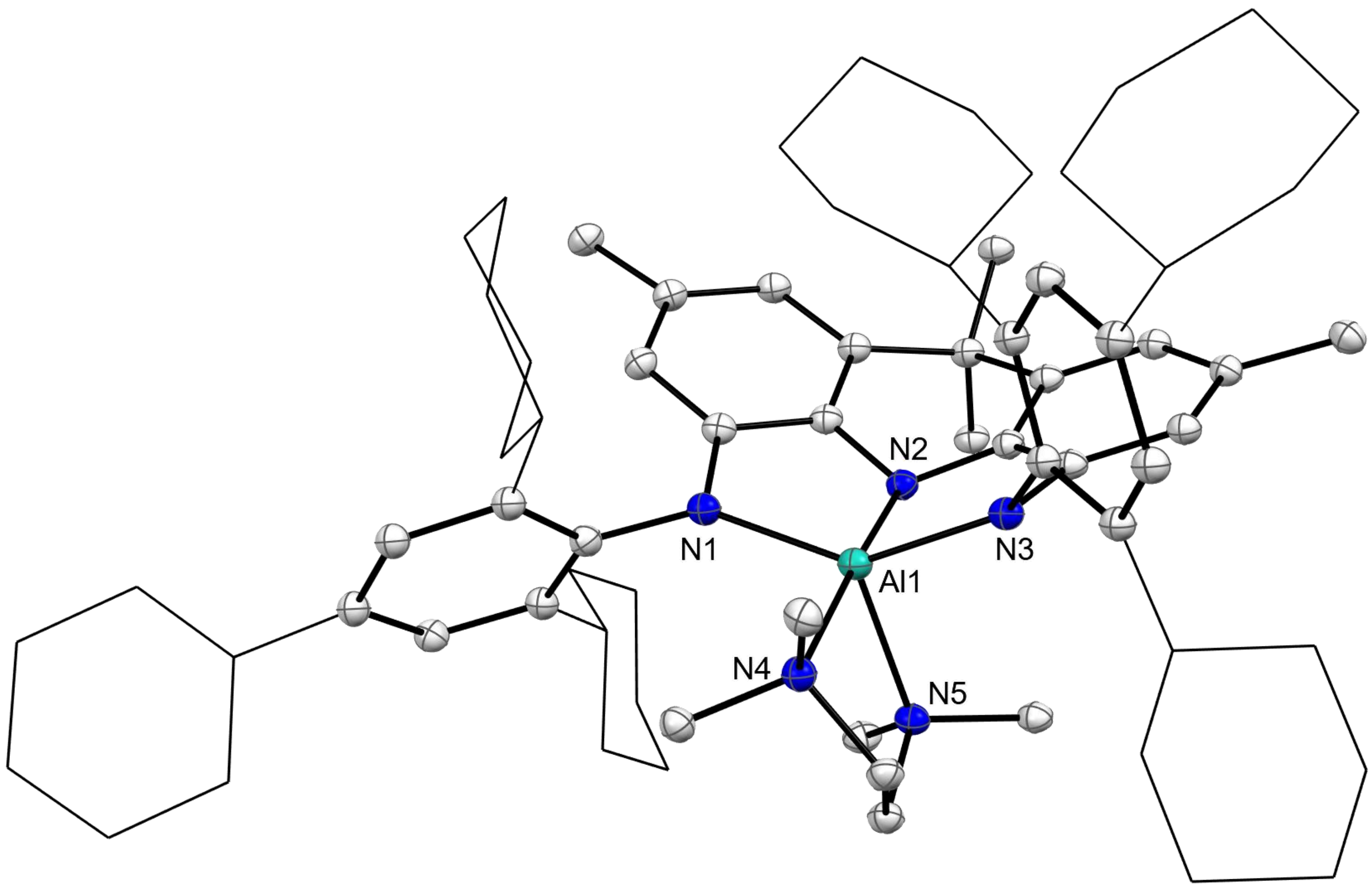

Figure S35: Molecular structure of **4**. Thermal ellipsoids shown at 30% probability; hydrogen atoms omitted; Cyclohexyl groups shown as wireframe for clarity.

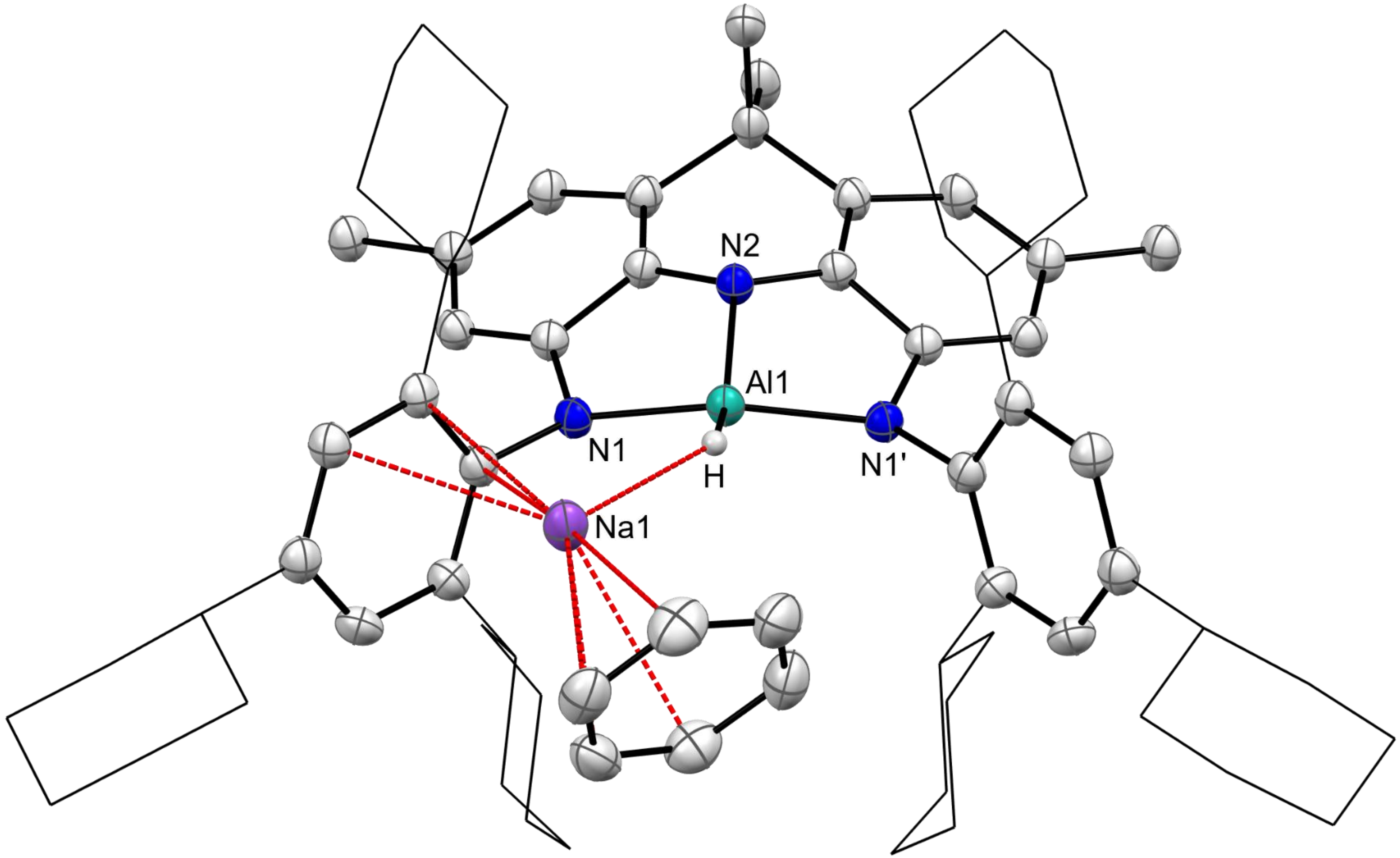

Figure S36: Molecular structure of **5**. Thermal ellipsoids shown at 30% probability; hydrogen atoms, except the hydride, omitted; Cyclohexyl groups shown as wireframe for clarity.

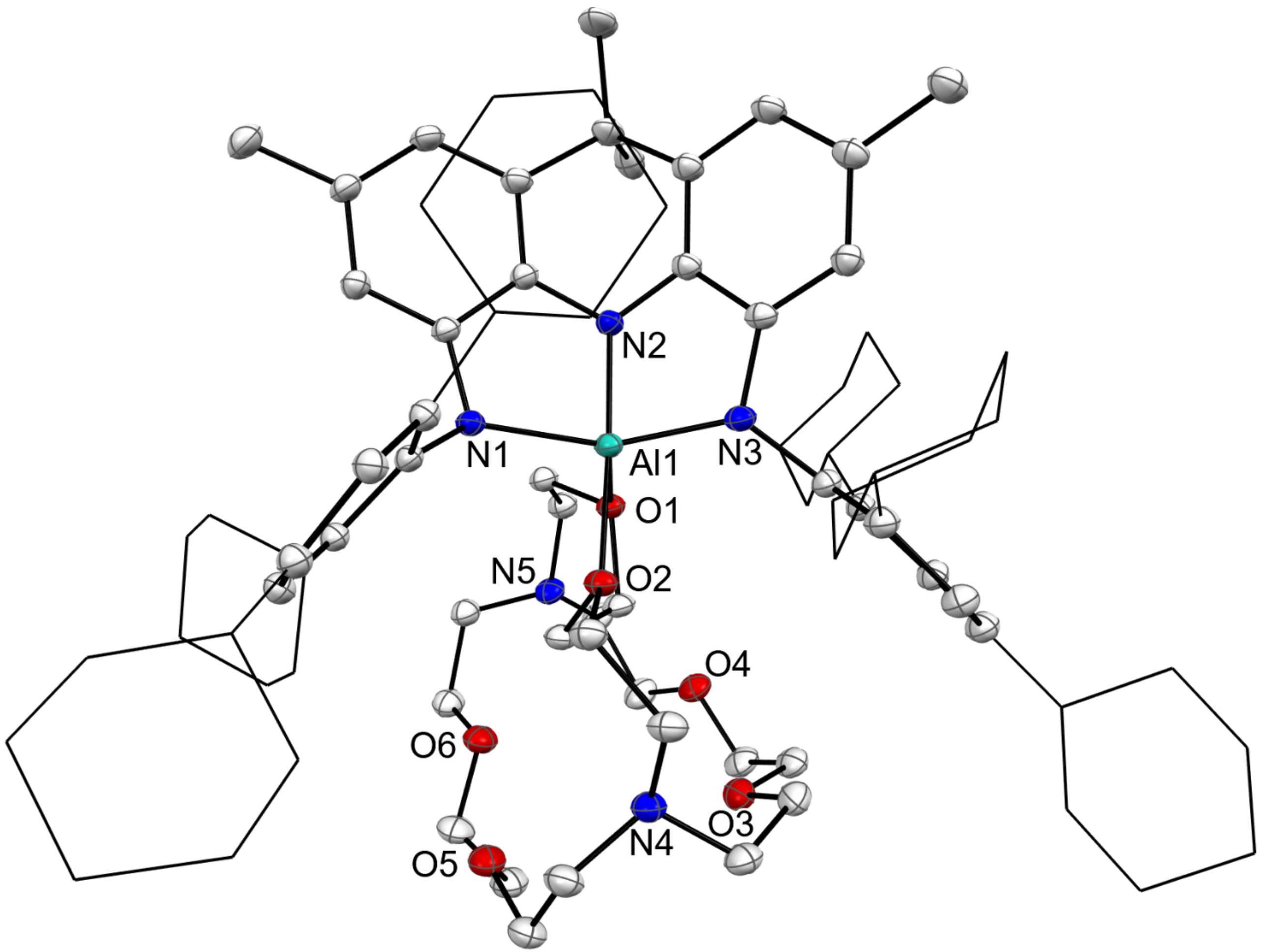

Figure S37: Molecular structure of **6**. Thermal ellipsoids shown at 30% probability; hydrogen atoms omitted; Cyclohexyl groups shown as wireframe for clarity.

3. Computational Details

Density Functional Theory (DFT) calculations were run using Gaussian 09 (Revision D.01).[S9] Geometry optimisations were performed without symmetry constraints using the single crystal X-ray diffraction data for **5** as the input geometry. Frequency calculations were performed on all stationary points to confirm their nature as minima (no imaginary frequency). The DFT calculations were carried out using the BP86 functional[S10] along with the Def2SVP basis set[S11] for all atoms. Dispersion corrections were included in our calculations by employing the D3 version of Grimme's dispersion with Becke-Johnson damping (keyword: empiricaldispersion=gd3bj).[S12] This level of theory showed good agreement with the solid-state data and has been used recently in a related study by Su, Wang, and co-workers.[S13] Computed IR stretching frequencies were scaled[S14] using the scaling factor benchmarked by Krossing and co-workers (BP86-D3(BJ)/def2-SVP, scaling factor of 0.9947).[S15] Atoms-In-Molecules Analysis were performed using the Multiwfn software package.[S16]

Optimized Geometry and Natural Bonding Orbital Calculations on 5

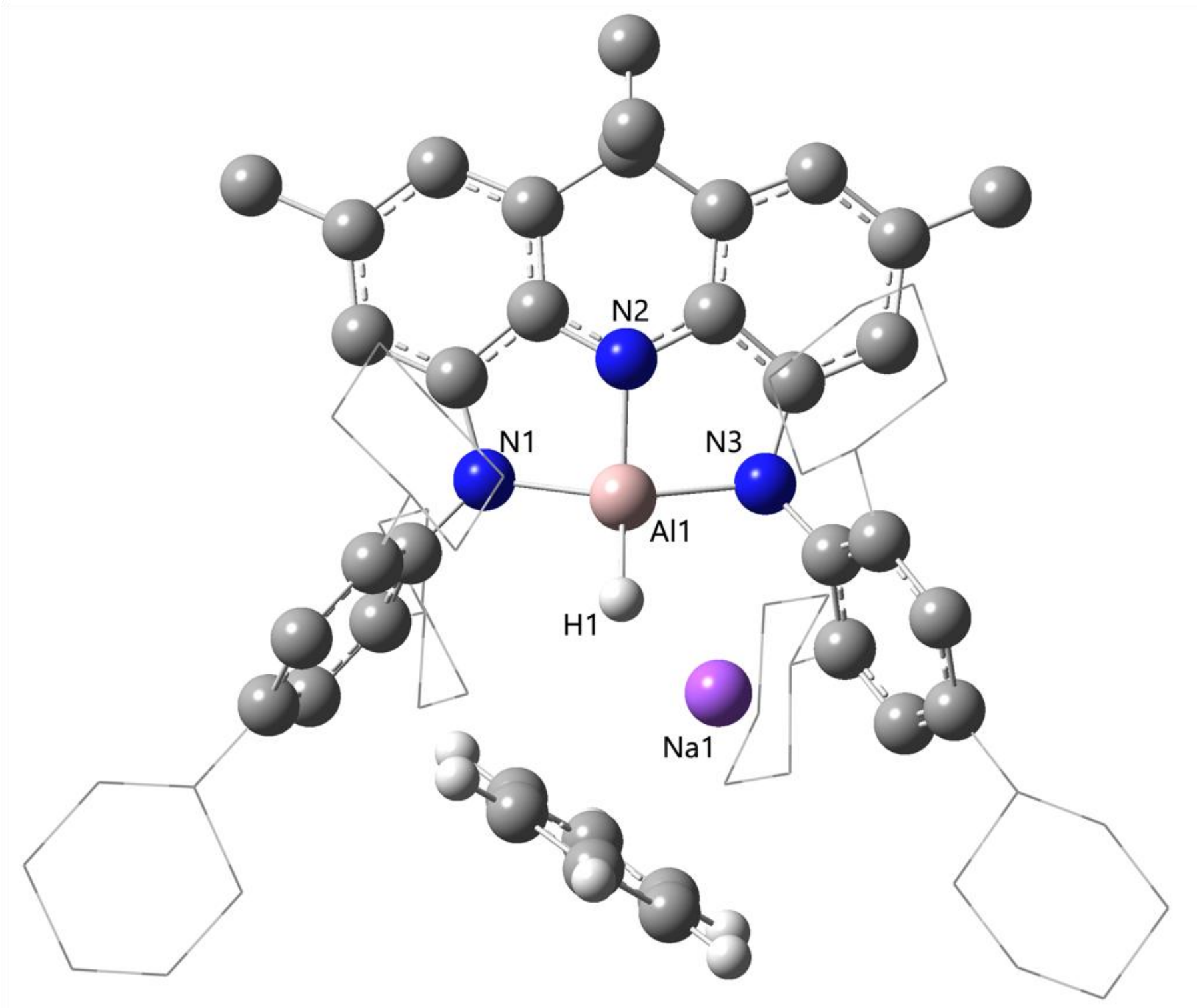

Figure S38: DFT optimized structure of **5**, with key atoms labelled. Most hydrogen atoms removed and cyclohexyl groups shown in wireframe for clarity.

Table S2: Select bond length (Å) and Natural Bonding Orbital (NBO) analysis data for **5**. Natural Population Analysis (NPA) charge and Wiberg Bond Index (WBI) data reported.

Bond	Bond length (Å)	NPA Charge		WBI	
Al1–N1	1.914	Al1	+1.643	Al1–N1	0.414
Al1–N2	1.852	H1	-0.515	Al1–N2	0.403
Al1–N3	1.950	Na1	+0.916	Al1–N3	0.371
Al1–H1	1.622	N1	-0.852	Al1–H1	0.650
Al1–Na1	3.454	N2	-0.809	Al1–Na1	0.011
Na1–H1	2.051	N3	-0.934	Na1–H1	0.023

Computed Infrared Spectra of 5

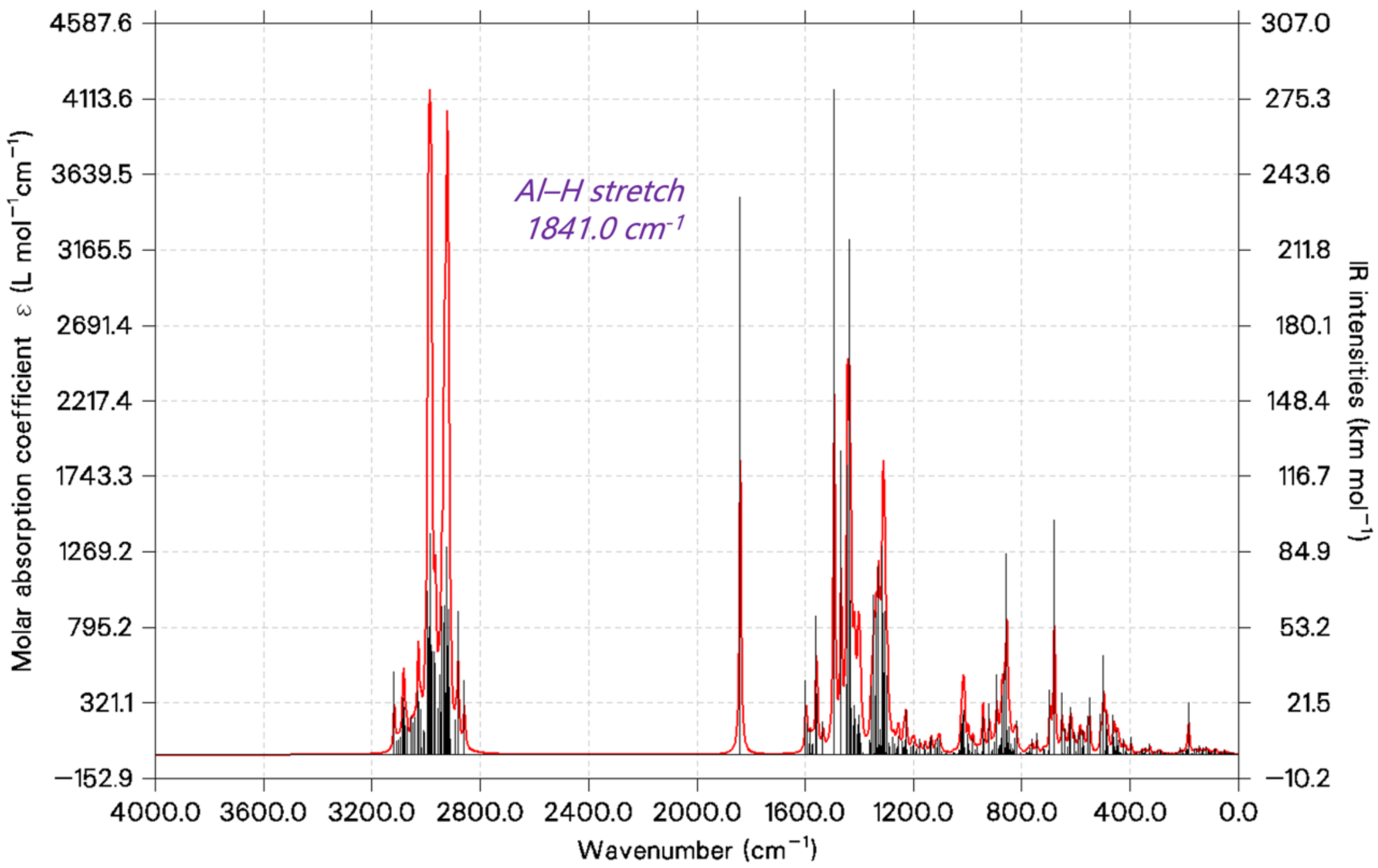

Figure S39: Computed IR spectra from the DFT frequency calculation of **5**, corrected for the level of theory (BP86-D3(BJ)/def2-SVP) using a scaling factor of 0.9947.

Select Frontier Molecular Orbitals of 5

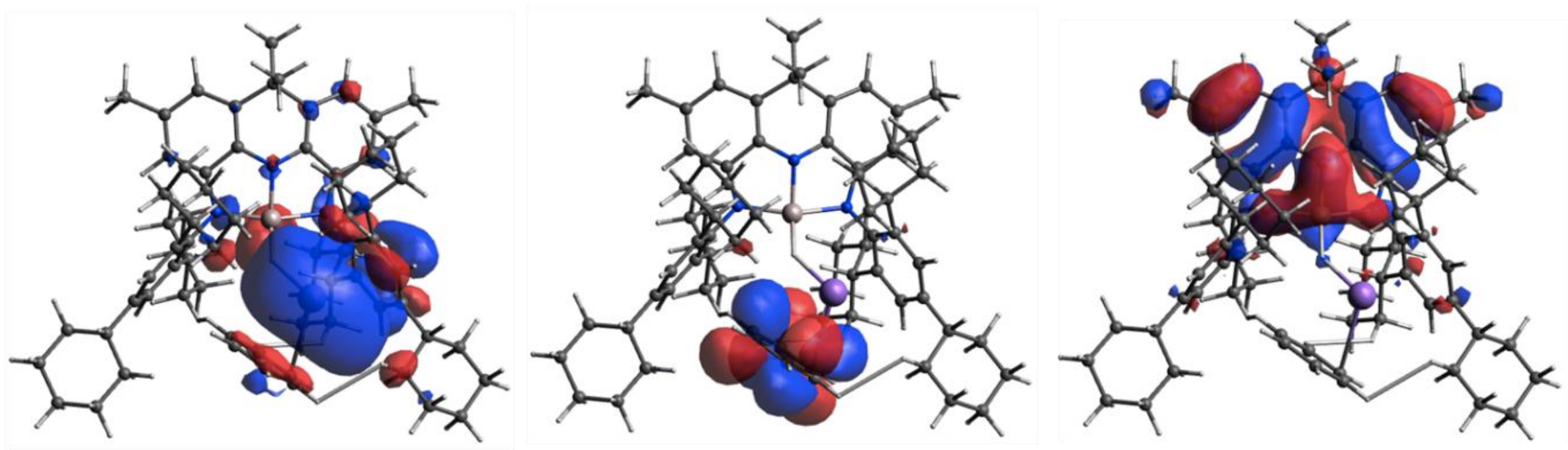

Figure S40: Select frontier molecular orbitals of **5**.

Atoms-In-Molecules (AIM) Analysis

Table S3: Select data from the Atoms-In-Molecules (AIM) analysis on **5.**

Bond	Density of all electrons ρ(r)	Laplacian of electron density ∇ 2ρ(r)	Lagrangian kinetic energy G(r)	Potential energy density V(r)	Energy density H(r)	Ellipticity of electron density ε
Al1–H1	0.064	0.252	0.015	-0.042	-0.027	0.008
Na1–H1	0.019	0.085	0.006	-0.007	-0.0008	0.025
Al1–N1	0.068	0.430	0.030	-0.047	-0.017	0.105
Al1–N2	0.079	0.542	0.039	-0.058	-0.020	0.098
Al1–N3	0.063	0.377	0.027	-0.043	-0.016	0.077

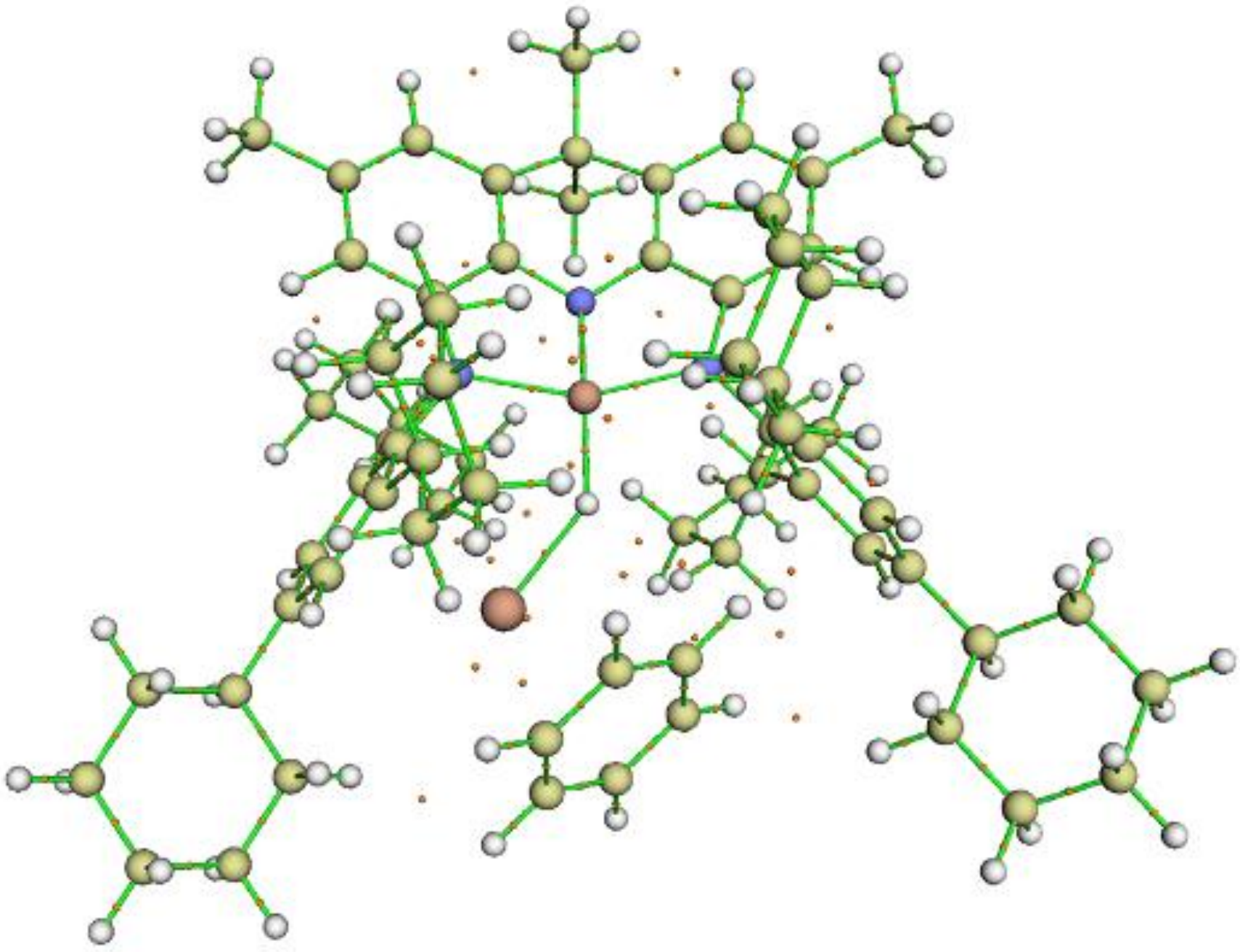

Figure S41: QTAIM molecular graph for **5**. Bonds are shown as green lines with bond critical points shown as orange dots. Atom colour scheme: aluminium = nude; sodium = nude; hydrogen = white; nitrogen = blue; carbon = yellow.

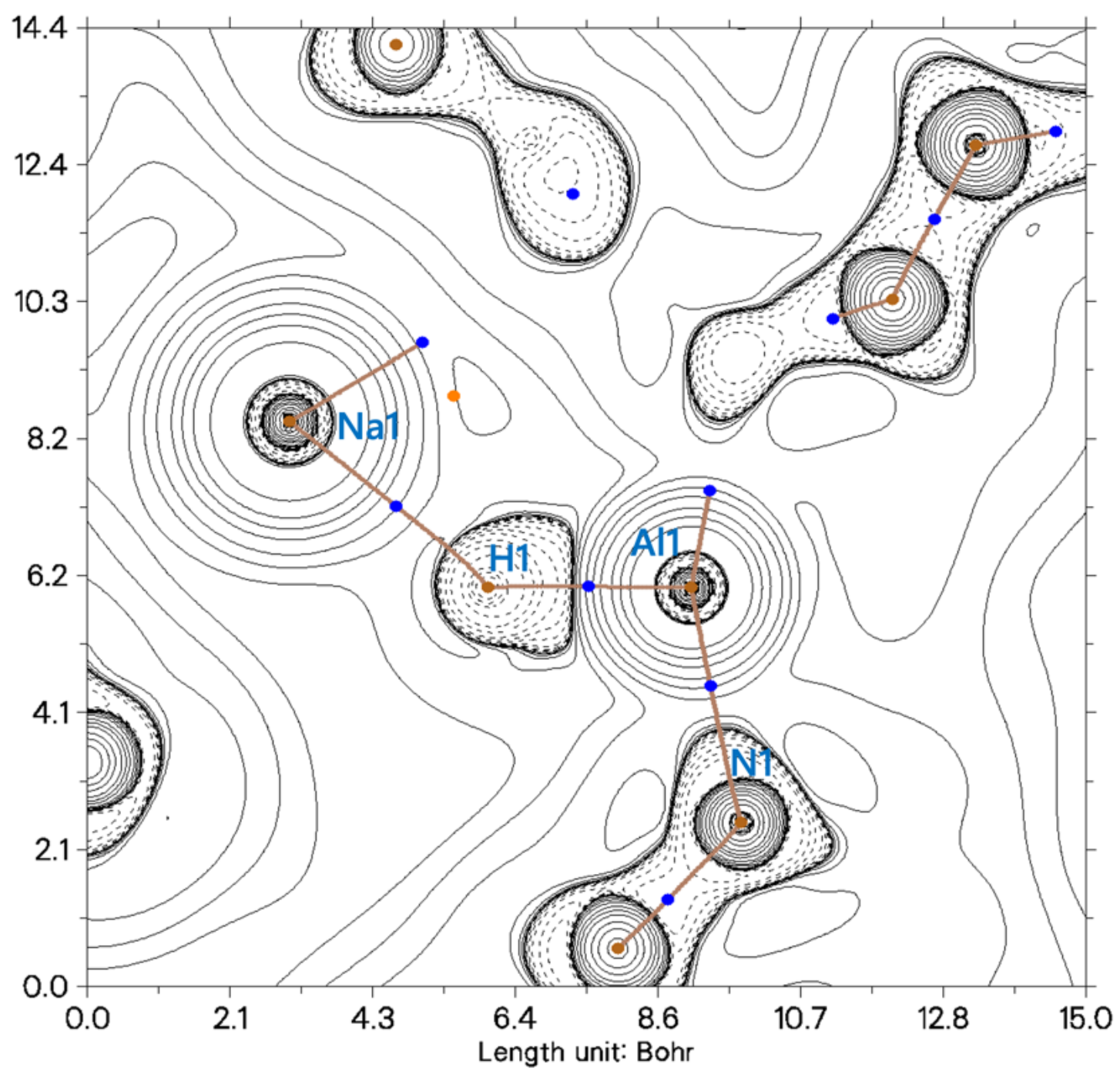

Figure S42: Laplacian of rho contour plot for **5**, showing the interaction between the Al, H, and Na atoms. Bond and ring critical points are shown as blue and orange dots, respectively.

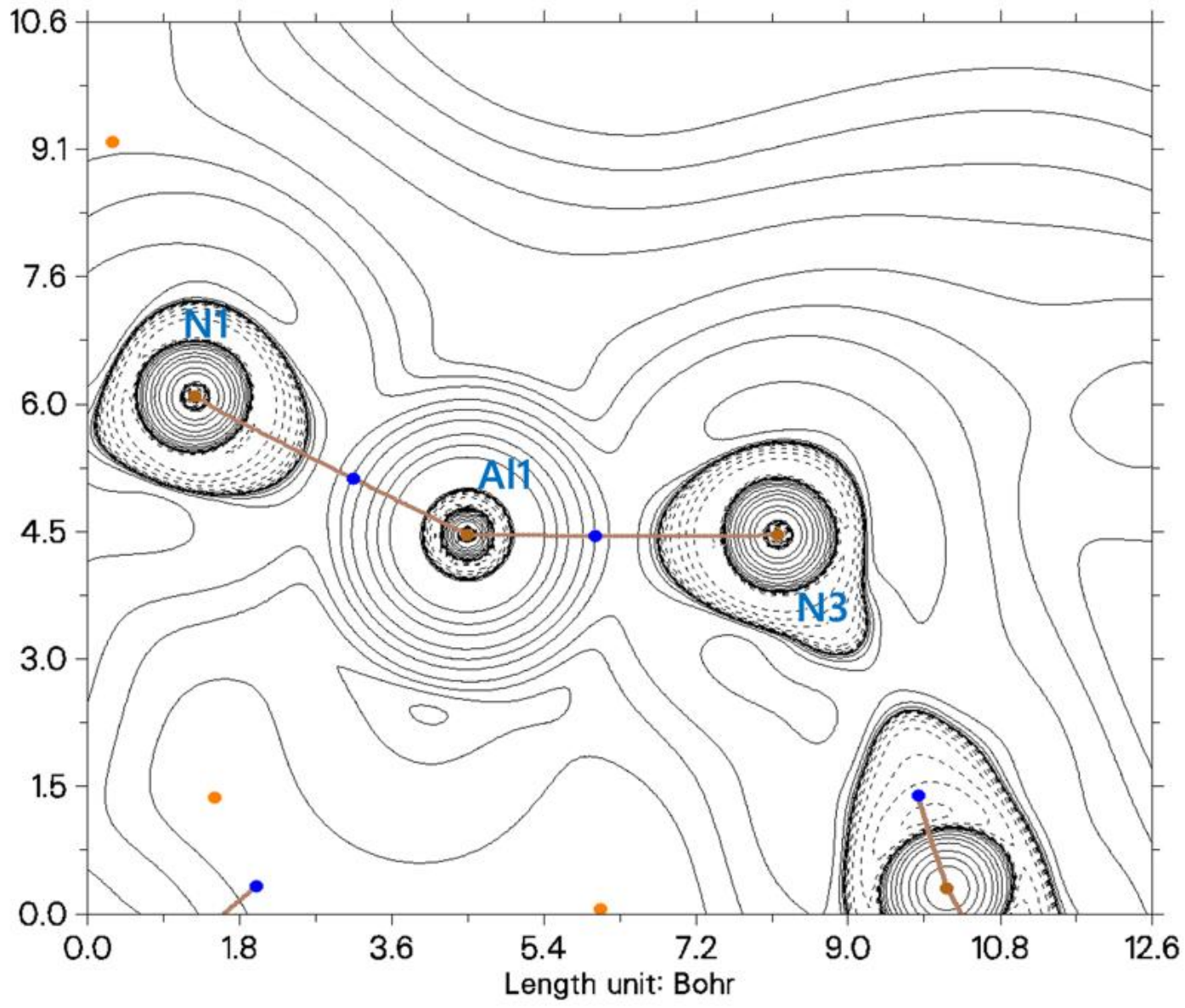

Figure S43: Laplacian of rho contour plot for **5**, showing the interaction between the Al and N atoms. Bond and ring critical points are shown as blue and orange dots, respectively.

Cartesian Coordinates

5.log

SCF (BP86-D3(BJ)) = -3328.97370664

E(SCF)+ZPE(0 K)= -3327.527212

H(298 K)= -3327.451633

G(298 K)= -3327.642534

Lowest Frequency = 6.2419cm^{-1}

Al	6.637638	5.866319	5.974080
N	6.989900	7.740765	5.818493
N	7.985856	5.883810	7.244096
C	8.611527	7.099441	7.434608
C	9.743964	9.624642	7.631578
C	8.036343	8.158369	6.669011
C	9.784021	7.225900	8.194154
C	8.594199	9.442144	6.818721
H	8.156083	10.300289	6.283922
C	5.063424	8.631501	7.743497
H	5.836155	7.835466	7.789443
C	6.021388	8.677238	5.386935
C	5.044063	9.140594	6.315683
C	10.345610	8.519549	8.278968
H	11.258195	8.689920	8.871163
C	3.990400	10.480112	4.541725
C	10.274899	5.954604	8.935633
C	5.956306	9.082189	4.022360
C	4.055678	10.041347	5.876762
H	3.301270	10.406885	6.594579
C	5.496149	9.699130	8.771822
H	6.452379	10.155074	8.442840
H	4.735980	10.514317	8.802674
C	10.316684	11.015796	7.801741
H	10.379769	11.555317	6.832659
H	11.335087	10.986336	8.239637
H	9.686302	11.640104	8.474099
C	3.444018	12.726198	3.460205
H	4.067560	12.466550	2.574519
H	4.124965	13.225444	4.181971
C	5.654123	9.070669	10.164266
H	6.492327	8.338004	10.125661

H	5.946294	9.842168	10.908648
C	4.368384	8.355144	10.611938
H	4.520359	7.860707	11.595702
H	3.563154	9.111280	10.763097
C	2.893504	11.424886	4.086259
H	2.311706	11.716968	4.991628
C	4.944504	9.979500	3.630543
H	4.889261	10.295921	2.577908
C	1.904083	10.738954	3.113539
H	1.486877	9.825562	3.589179
H	2.467109	10.390310	2.217538
C	2.316082	13.674672	3.026124
H	1.759164	14.016784	3.929025
H	2.741328	14.588692	2.558451
C	3.744682	7.960611	8.178677
H	2.917196	8.707365	8.191946
H	3.464674	7.189034	7.427903
C	3.899758	7.326888	9.568880
H	2.949122	6.848353	9.891925
H	4.656009	6.512789	9.500535
C	0.778332	11.687547	2.675187
H	0.103791	11.175791	1.955066
H	0.151860	11.943089	3.561027
C	1.337128	12.982423	2.063787
H	0.509725	13.670865	1.787284
H	1.870373	12.737215	1.116180
C	6.980025	8.539708	3.036566
H	7.133960	7.465229	3.295400
C	6.548436	8.603448	1.560152
H	6.384225	9.664590	1.264076
H	5.570436	8.089484	1.424078
C	7.612816	7.996913	0.632269
H	7.718014	6.913966	0.861960
H	7.281178	8.060557	-0.426661
C	8.358551	9.221734	3.218802
H	8.681109	9.134047	4.274272
H	8.239969	10.309641	3.007256
C	9.419878	8.619960	2.287790
H	9.594313	7.558789	2.580610
H	10.390331	9.142594	2.427194
C	8.979519	8.673027	0.817478
H	9.738806	8.194633	0.161774
H	8.909593	9.737110	0.493278

H	5.030152	5.806755	5.762850
N	7.118762	3.985589	5.789346
C	8.740758	4.737663	7.357131
C	10.145468	2.340071	7.387780
C	8.256146	3.655458	6.564798
C	9.930414	4.692951	8.101804
C	8.965074	2.442589	6.605705
H	8.615833	1.579070	6.015771
C	5.848588	2.715080	8.081748
H	6.648144	3.478115	8.035376
C	6.115182	3.035655	5.556103
C	5.461189	2.378347	6.653915
C	10.625336	3.463402	8.103836
H	11.558166	3.357373	8.679844
C	3.880017	1.300451	5.088841
C	5.607867	2.848794	4.225769
C	4.379003	1.510885	6.392564
H	3.886264	1.000344	7.238393
C	6.446566	1.526050	8.862554
H	7.276465	1.087900	8.271478
H	5.675943	0.729794	8.987881
C	10.878321	1.017218	7.457180
H	10.997298	0.559561	6.451859
H	11.889581	1.134708	7.896472
H	10.331462	0.276307	8.082908
C	3.001025	-0.751597	3.854557
H	3.320102	-0.313182	2.881565
H	3.867120	-1.336241	4.230335
C	6.954960	1.990245	10.234809
H	7.793725	2.705154	10.075404
H	7.374625	1.132729	10.803057
C	5.840060	2.674111	11.042821
H	6.232556	3.042531	12.014673
H	5.052388	1.923616	11.286252
C	2.686417	0.394938	4.842735
H	2.418490	-0.073442	5.818025
C	4.503785	1.993673	4.025295
H	4.117215	1.856235	3.004039
C	1.447253	1.189073	4.365458
H	1.205339	1.978487	5.112310
H	1.707609	1.720496	3.421327
C	1.783221	-1.659608	3.624699
H	1.528189	-2.177670	4.577720

H	2.032905	-2.457968	2.893723
C	4.701865	3.374568	8.880985
H	3.849691	2.665874	9.000580
H	4.314796	4.259292	8.319397
C	5.201923	3.833532	10.259917
H	4.372405	4.289983	10.840894
H	5.960715	4.634835	10.109331
C	0.230240	0.282666	4.125258
H	-0.628193	0.881321	3.750928
H	-0.093959	-0.154878	5.097291
C	0.564399	-0.854734	3.146539
H	-0.315078	-1.520064	3.012936
H	0.784003	-0.420353	2.143935
C	6.255258	3.610072	3.079512
H	6.310084	4.681366	3.397115
C	5.469565	3.578980	1.758151
H	5.438556	2.534050	1.372631
H	4.414417	3.879427	1.928686
C	6.120610	4.487094	0.705998
H	6.069502	5.541689	1.061244
H	5.547486	4.451101	-0.245295
C	7.717845	3.169078	2.827379
H	8.283121	3.194946	3.779197
H	7.707587	2.111258	2.478068
C	8.387036	4.068215	1.778064
H	8.466361	5.100224	2.192272
H	9.428736	3.731163	1.591434
C	7.590339	4.106502	0.464130
H	8.057337	4.812453	-0.255883
H	7.628412	3.101160	-0.014640
C	2.905132	7.112306	4.080787
H	3.628508	7.939023	4.111850
C	1.862401	7.052939	5.026205
H	1.777044	7.848388	5.781596
C	0.943170	5.982996	5.002501
H	0.123857	5.941714	5.738258
C	1.061932	4.973087	4.021989
H	0.338074	4.144022	3.990462
C	2.101710	5.038601	3.069442
H	2.193164	4.251867	2.304133
C	3.021654	6.106460	3.102271
H	3.842652	6.152905	2.370082
Na	3.505912	4.435550	5.703696

C	9.467192	5.855405	10.263000
H	9.757235	4.947100	10.832803
H	9.643981	6.751016	10.894942
H	8.380512	5.795168	10.047101
C	11.776424	6.030527	9.253297
H	12.110805	5.131509	9.809629
H	12.379059	6.111572	8.326066
H	12.001886	6.907330	9.893330

4. References

[S1] Liu, H.; Wang, N.-D.; Du, D.-M.; Direct Experimental Evidence for the Priority of Flexible Ligand Skeleton in Asymmetric Friedel-Crafts Alkylation of Indole with Nitroalkenes. *Lett. Org. Chem.* **2010**, *7*, 114–120.

[S2] Richardson, G. M.; Evans, M. J.; Rajeshkumar, T.; McCone, J. A. J.; Cameron, S. A.; Maron, L.; Jones, C.; Anker, M. D.; Synthesis and Reactivity of Discrete Europium(II) Hydride Complexes. *Chem. Eur. J.* **2024**, *30*, e202400681.

[S3] Savka, R.; Plenio, H.; Metal Complexes of Very Bulky N,N′-Diarylimidazolylidene N-Heterocyclic Carbene (NHC) Ligands with 2,4,6-Cycloalkyl Substituents. *Eur. J. Inorg. Chem.* **2014**, *2014*, 6246–6253.

[S4] Cowieson, N. P.; Aragao, D.; Clift, M.; Ericsson, D. J.; Gee, C.; Harrop, S. J.; Mudie, N.; Panjikar, S.; Price, J. R.; Riboldi-Tunnicliffe, A.; Williamson, R.; Caradoc-Davies, T.; MX1: a bending-magnet crystallography beamline serving both chemical and macromolecular crystallography communities at the Australian Synchrotron. *J. Synchrotron Rad.* **2015**, *22*, 187–190.

[S5] Aragão, D.; Aishima, J.; Cherukuvada, H.; Clarken, R.; Clift, M.; Cowieson, N. P.; Ericsson, D. J.; Gee, C. L.; Macedo, S.; Mudie, N.; Panjikar, S.; Price, J. R.; Riboldi-Tunnicliffe, A.; Rostan, R.; Williamson, R.; Caradoc-Davies, T. T.; MX2: a high-flux undulator microfocus beamline serving both the chemical and macromolecular crystallography communities at the Australian Synchrotron. *J. Synchrotron Rad.* **2018**, *25*, 885–891.

[S6] McPhillips, T. M.; McPhillips, S. E.; Chiu, H.-J.; Cohen, A. E.; Deacon, A. M.; Ellis, P. J.; Garman, E.; Gonzalez, A.; Sauter, N. K.; Phizackerley, R. P.; Soltis, S. M.; Kuhn, P.; *Blu-Ice* and the *Distributed Control System*: software for data acquisition and instrument control at macromolecular crystallography beamlines. *J. Synchrotron Rad.* **2002**, *9*, 401–406.

[S7] Kabsch, W.; *J. Appl. Crystallogr.* **1993**, *26*, 795–800.

[S8] Sheldrick, G. M.; SHELX-18, **2018**, University of Göttingen.

[S9] Frisch, M. J.; Trucks, G. W.; Schlegel, H. B.; Scuseria, G. E.; Robb, M. A.; Cheeseman, J. R.; Scalmani, G.; Barone, V.; Mennucci, B.; Petersson, G. A.; Nakatsuji, H.; Caricato,

M.; Li, X.; Hratchian, H. P.; Izmaylov, A. F.; Bloino, J.; Zheng, G.; Sonnenberg, J. L.; Hada, M.; Ehara, M.; Toyota, K.; Fukuda, R.; Hasegawa, J.; Ishida, M.; Nakajima, T.; Honda, Y.; Kitao, O.; Nakai, H.; Vreven, T.; Montgomery, J. A., Jr.; Peralta, J. E.; Ogliaro, F.; Bearpark, M.; Heyd, J. J.; Brothers, E.; Kudin, K. N.; Staroverov, V. N.; Keith, T.; Kobayashi, R.; Normand, J.; Raghavachari, K.; Rendell, A.; Burant, J. C.; Iyengar, S. S.; Tomasi, J.; Cossi, M.; Rega, N.; Millam, J. M.; Klene, M.; Knox, J. E.; Cross, J. B.; Bakken, V.; Adamo, C.; Jaramillo, J.; Gomperts, R.; Stratmann, R. E.; Yazyev, O.; Austin, A. J.; Cammi, R.; Pomelli, C.; Ochterski, J. W.; Martin, R. L.; Morokuma, K.; Zakrzewski, V. G.; Voth, G. A.; Salvador, P.; Dannenberg, J. J.; Dapprich, S.; Daniels, A. D.; Farkas, O.; Foresman, J. B.; Ortiz, J. V.; Cioslowski, J.; Fox, D. J.; Gaussian 09, Revision D.01; Gaussian, Inc.: Wallingford, CT, **2013**.

[S10] Becke, A. D.; Density-functional exchange-energy approximation with correct asymptotic behavior. *Phys Rev A*. **1988**, *38*, 3098–3100.

[S11] (a) Weigend, F.; Ahlrichs, R.; Balanced basis sets of split valence, triple zeta valence and quadruple zeta valence quality for H to Rn: Design and assessment of accuracy. *Phys. Chem. Chem. Phys*., **2005**, *7*, 3297-305; (b) Weigend, F.; Accurate Coulomb-fitting basis sets for H to Rn. *Phys. Chem. Chem. Phys.*, **2006**, *8*, 1057–1065.

[S12] (a) Grimme, S.; Ehrlich, S.; Goerigk, L.; Effect of the damping function in dispersion corrected density functional theory. *J. Comput. Chem*., **2011**, *32*, 1456−1465; (b) Grimme, S.; Antony, J.; Ehrlich, S.; Krieg, H.; A consistent and accurate ab initio parametrization of density functional dispersion correction (DFT-D) for the 94 elements H-Pu. *J. Chem. Phys*. **2010**, *132*, 154104–154119.

[S13] Wang, W.; Bao, M.; Dai, Y.; Liu, X.; Liu, C.; Liu, C.; Su, Y.; Wang, X.; Isolable Pincer-type Dianionic Dialane(6). *Organometallics*, **2022**, *41*, 680–685.

[S14] Merrick, J. P.; Moran, D.; Radom, L.; An Evaluation of Harmonic Vibrational Frequency Scale Factors. *J. Phys. Chem. A*, **2007**, *111*, 116883–11700.

[S15] Unkrig, W.; Zhe, F.; Tamim, R.; Oesten, F.; Kratzert, D.; Krossing, I.; Cationic Niobium-Sandwich and Piano-Stool Complexes. *Chem. Eur, J.*, **2021**, *27*, 758–765.

[S16] Lu, T.; Chen, F.; Multiwfn: A multifunctional wavefunction analyzer. *J. Comput. Chem*. **2012**, *33*, 580–592.
